# Neuroprotective Metabolites from Vietnamese Marine Derived Fungi of *Aspergillus* and *Penicillium* Genera

**DOI:** 10.3390/md18120608

**Published:** 2020-11-30

**Authors:** Elena V. Girich, Anton N. Yurchenko, Olga F. Smetanina, Phan Thi Hoai Trinh, Ngo Thi Duy Ngoc, Mikhail V. Pivkin, Roman S. Popov, Evgeny A. Pislyagin, Ekaterina S. Menchinskaya, Ekaterina A. Chingizova, Shamil S. Afiyatullov, Ekaterina A. Yurchenko

**Affiliations:** 1 G.B. Elyakov Pacific Institute of Bioorganic Chemistry, Far Eastern Branch of Russian Academy of Sciences, 690950 Vladivostok, Russia; ev.ivanets@yandex.ru (E.V.G.); smetof@rambler.ru (O.F.S.); oid27@mail.ru (M.V.P.); prs_90@mail.ru (R.S.P.); pislyagin@hotmail.com (E.A.P.); ekaterinamenchinskaya@gmail.com (E.S.M.); martyyas@mail.ru (E.A.C.); afiyat@piboc.dvo.ru (S.S.A.); dminae@mail.ru (E.A.Y.); 2 Department of Marine Biotechnology, Nhatrang Institute of Technology Research and Application, Vietnam Academy of Science and Technology, Nha Trang 650000, Vietnam; phanhoaitrinh@nitra.vast.vn (P.T.H.T.); ngoduyngoc@nitra.vast.vn (N.T.D.N.)

**Keywords:** *Aspergillus terreus*, *Aspergillus flocculosus*, *Penicillium* sp., marine-derived fungi, South China Sea, secondary metabolites, asterriquinones, polyketides, neuroprotective activity, reactive oxygen species

## Abstract

Low molecular weight secondary metabolites of marine fungi *Aspergillus flocculosus*, *Aspergillus terreus* and *Penicillium* sp. from Van Phong and Nha Trang Bays (Vietnam) were studied and a number of polyketides, bis-indole quinones and terpenoids were isolated. The structures of the isolated compounds were determined by 1D and 2D NMR and HR-ESI-MS techniques. Stereochemistry of some compounds was established based on ECD data. A chemical structure of asterriquinone F (**6**) was thoroughly described for the first time. Anthraquinone (**13**) was firstly obtained from a natural source. Neuroprotective influences of the isolated compounds against 6-OHDA, paraquat and rotenone toxicity were investigated. 4-Hydroxyscytalone (**1**), 4-hydroxy-6-dehydroxyscytalone (**2**) and demethylcitreoviranol (**3**) have shown significant increasing of paraquat- and rotenone-treated Neuro-2a cell viability and anti-ROS activity.

## 1. Introduction

Marine fungi have to adapt to difficult environments and therefore they have a unique secondary metabolism that differs from terrestrial strains [1,2]. For instance, entomopathogenic fungus *Beauveria (Isaria) felina* usually produces a big variety of insecticide depsipeptides [3] but its marine strain was a source of rare oxygenated isochromenes and unique cytotoxic α-unsubstituted pyran polyketides [4]. Moreover, marine areas differ in a number of characteristics (salinity, depth, content of nutrients and organic compounds in the water), and therefore, fungi isolated from different seas can also produce different substances [5].

Van Phong and Nha Trang Bays are located in the central part of the Vietnamese coastline of the South China Sea [6]. Water in these sea areas is warm, and the temperature ranges from 23 °C in January up to 28 °C in May–June due to being closely connected with a monsoon climate. Salinity of coastal waters is close to the ocean normal one, ranging from 32‰ to 34‰. The living conditions of these bays are ideal for most marine organisms in the tropics [7,8]. Moreover, the coral reefs of these bays are unique due to connection with the open sea and wave action, thereby leading to the formation of reefs of inner bays and straits, and reefs of open capes and islands [9]. In particular, the coral reefs in Nha Trang and Van Phong Bays are considered as high diverse ecosystems with many valuable marine micro- and macroorganisms producing various bioactive compounds [8,10].

The marine sediment-derived strain *Beauveria (Isaria) felina* from Van Phong Bay was reported as a source of highly oxygenated chromene derivatives oxirapentyns with cytotoxic activity against a number of cancer cell lines [11] and unique prostate cancer-toxic polyketide isariketide A [12]. Moreover, coincubation of *B. felina* with *Aspergillus sulphureus* KMM 4640 resulted in isolation of oxirapentyn L [12] and cytotoxic diorcinol J [13].

The strain *Aspergillus terreus* from a sample of Zoantharia coral collected on the shores of Van Phong Bay was found as a source of questin and terrein [14]. It was the first report about terrein-induced enhancement of a heat shock protein 70 kDa (Hsp70) expression.

Marine fungus *Aspergillus niveoglaucus* from the sediment sample collected from Nha Trang Bay was reported as a source of polyketides niveoglaucin A and flavoglaucin and a number of echinulin-related compounds that demonstrated antioxidant and neuroprotective effects in different in vitro Parkinson’s disease models [15,16].

Marine fungus *Penicillium* sp. KMM 4672 isolated from brown alga *Padina* sp. (Van Phong Bay) was reported as a source of melatonin analogue 6-hydroxy-*N*-acetyl-β-oxotryptamine, 3-methylorsellinic acid and 8-methoxy-3,5-dimethylisochroman-6-ol, which effectively protected Neuro-2a cells against 6-hydroxydopamine (6-OHDA)-induced neuronal death [17]. 

The strain *Aspergillus flocculosus* from a sediment sample (Nha Trang Bay) produced drimane derivative 6β,9α,14-trihydroxycinnamolide and 9α,14-dihydroxy-6β-p-nitrobenzoylcinnamolide with high cytotoxic activity against mouse Neuro-2a and human 22Rv1 and MCF-7 line cells [18]. Moreover, diketopiperazine mactanamide from this strain has shown influence on osteoclast differentiation [19] and the fungistatic effect [20] and radical scavenging [21], antioxidant and neuroprotective activities in 6-OHDA-treated cell model of Parkinson’s disease [17].

Thus, there are a few examples of reports about marine fungi from Van Phong and Nha Trang Bays (similar to other parts of Vietnamese coast) as producers of bioactive metabolites including neuroprotective compounds.

To upgrade our knowledge in this field, we continue to investigate the secondary metabolites of *Penicillium* sp. KMM 4672 and *A. flocculosus* and a new strain of *A. terreus* isolated from leaves of an unidentified mangrove tree. Neuroprotective effects of isolated compounds were studied in different toxin-induced cell models of Parkinson’s disease.

## 2. Results

### 2.1. Isolation and Identification of Compounds

The thoroughly chromatographic separation of medium-polarity fractions of *Penicillium* sp. KMM 4672 with silica gel, sephadex LH-20 and subsequent reversed-phase HPLC to afford **1**–**4**. The structure of 4-hydroxy-3,6-dimethyl-2-pyrone (**4**) was earlier reported by us [22], whereas planar structures of compounds **1**-**3** were established by 1D and 2D NMR and high resolution electrospray ionization mass-spectrometry (HR-ESI-MS) and found to be reported as 4-hydroxyscytalone (**1**) [23], 4-hydroxy-6-dehydroxyscytalone (**2**) [24] and demethylcitreoviranol (**3**) [25]. 

Relative configurations of 3-OH and 4-OH of compounds **1** were determined as *anti* based on ^3^J_H3-H4_ = 7.1 Hz [23,26], and its absolute stereochemistry was suggested as 3*S*,4*S* based on a comparison of the optical rotation value ([α]_D_^20^ +73°) with literature data ([α]_D_^20^ −75° [23] and [α]_D_^20^ +58° [27]). Finally, the structure of **1** was established as (3*S*,4*S*)-4-hydroxyscytalone.

Relative configurations of aliphatic hydroxy groups at C-3 and C-4 in 4-hydroxy-6-dehydroxyscytalone (**2**) were established as *anti* based on ^3^J_H3-H4_ = 6.4 Hz. Absolute stereochemistry of **2** was determined as 3*S*,4*S* by comparison of its experimental CD data (Supplementary Materials, Figure S7) with literature data [27] and experimental CD data for **1** (Supplementary Materials, Figure S3). Thus, the structure of **2** was established as (3*S*,4*S*)-4-hydroxy-6-dehydroxyscytalone.

**Figure 1:**
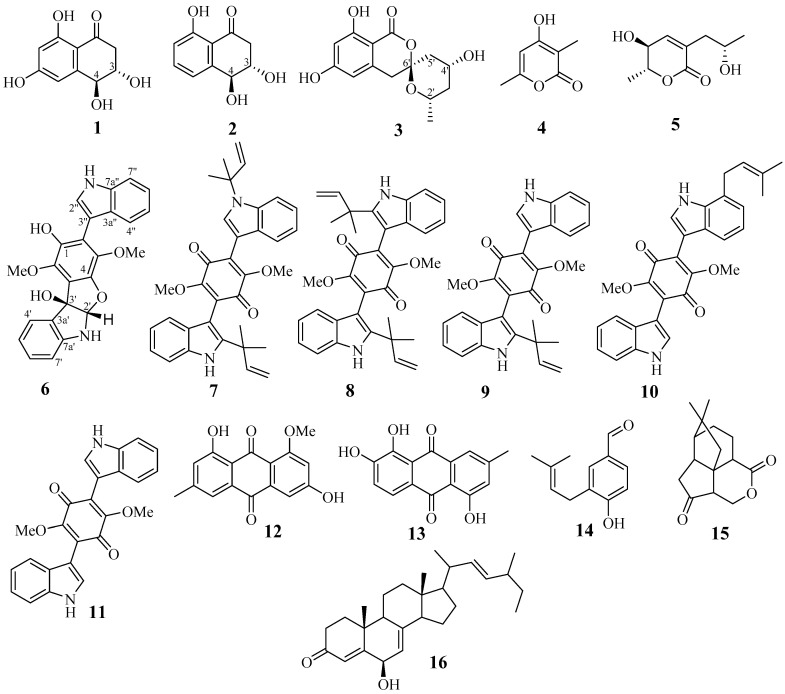
Structures of isolated compounds.

The vicinal coupling constants between H-2’ and H-3’a (^3^J = 11.8 Hz), H-2’ and H-3’b (^3^J = 2.1 Hz), H-3’a and H-4’ (^3^J = 11.8 Hz) and H-4’ and H-5’a (^3^J = 11.1 Hz) and W-type coupling constant between H-3’b and H-5’b (^5^J = 11.1 Hz) and comparing these data with literature values [28] revealed a relative stereochemistry of demethylcitreoviranol (**3**) as depicted (Figure 1). Unfortunately, we were not able to determine an absolute stereoconfigurations of **3** due to an insufficient amount of this compound.

A chemical composition of sediment-derived fungus *Aspergillus flocculosus* was recently studied by us and reported earlier [18]. Dihydroaspyrone (**5**) was the main metabolite of the extract of this fungus.

The fungus *Aspergillus terreus* LM.1.5 was cultivated for 21 days on modified rice medium. The dry EtOAc extract of the culture was separated successively over a column of silica gel and by normal-phase and reversed-phase HPLC to afford pure compounds **6**–**16** (Figure 1). 

Pseudo-molecular peak [M+Na]^+^ at *m/z* 439.1257 in the (+)-HR-ESI-MS spectrum of **6** suggested the molecular formula as C_24_H_20_N_2_O_5_ (calculate for C_24_H_20_N_2_O_5_Na, 439.1264), which was confirmed by NMR data. A thorough analysis of ^1^H and ^13^C NMR spectra together with DEPT and HSQC data (Table 1) indicated the presence of two methoxy groups (δ_C_ 61.5, 60.4; δ_H_ 4.12, 3.58), one oxygen-bearing *sp*^3^-metine carbon (δ_C_ 104.6; δ_H_ 6.24), one oxygenated quaternary *sp*^3^-carbon (δ_C_ 90.3), nine *sp*^2^-methine carbons (δ_C_ 130.1, 125.0, 124.8, 122.7, 120.4, 120.3, 120.1, 111.4, 109.9; δ_H_ 7.78, 7.47, 7.43, 7.29, 7.24, 7.18, 7.14, 6.88, 6.70), 11 quaternary *sp*^2^-carbons (δ_C_ 148.6, 144.9, 141.3, 139.1, 138.3, 136.2, 129.7, 126.5, 121.6, 116.7, 106.7) and two heteroatom-bonded protons (δ_H_ 8.44, 5.06). 

**Figure 2:**
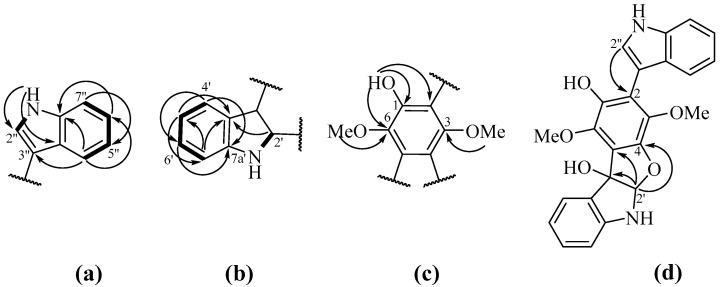
The key HMBC (shown by arrows) and ^1^H-^1^H COSY (shown by bold bonds) correlations in different moieties of **6** (**a**, **b**—Indole moieties, **c**—hydroquinone moiety, **d**—whole molecule).

HMBC correlations (Figure 2a) from 1”-NH (δ_H_ 8.44) to C-3” (δ_C_ 106.7), C-3a’’ (δ_C_ 126.5), from H-2” (δ_H_ 7.29) to C-3” (δ_C_ 106.7), C-3a’’ and C-7a’’ (δ_C_ 136.2), from H-4” (δ_H_ 7.47) to C-3”, C-6” (δ_C_ 122.7) and C-7a’’, from H-5” (δ_H_ 7.14) to C-3a’’ and C-7” (δ_C_ 111.4), from H-6” (δ_H_ 7.24) to C-4” (δ_C_ 120.3) and C-7a’’, from H-7” (δ_H_ 7.43) to C-3a’’ and C-5” (δ_C_ 120.4) and ^1^H-^1^H COSY correlations (Figure 2a) between 1”-NH/H-2”, H-4”/H-5”, H-5”/H-6” and H-6”/H-7” indicated the presence of a monosubstituted indole fragment. HMBC correlations (Figure 2b) from H-2’ (δ_H_ 6.24) to C-3’ (δ_C_ 90.3), C-3a’ (δ_C_ 129.7) and C-7a’ (δ_C_ 148.6), from H-4’ (δ_H_ 7.78) to C-3’, C-7a’ and C-6’ (δ_C_ 130.1), from H-5’ (δ_H_ 6.88) to C-3a’, C-7’ (δ_C_ 109.9), from H-6’ (δ_H_ 7.18) to C-4’ (δ_C_ 125.0) and C-7a’, from H-7’ (δ_H_ 6.70) to C-3a’ and C-5’(δ_C_ 120.1), ^1^H-^1^H COSY correlations (Figure 2b) between H-4’/H-5’, H-5’/H-6’, and H-6’/H-7’ and value of chemical shifts of C-2’ (δ_C_ 104.6) and C-3’ indicate of the presence of disubstituted indoline moiety. The remaining signals of quaternary *sp*^2^-carbons (C-1, δ_C_ 141.3; C-2, δ_C_ 116.7; C-3, δ_C_ 138.4; C-4, δ_C_ 144.9; C-5, δ_C_ 121.6 and C-6, δ_C_ 139.1) and HMBC correlations (Figure 2c) namely from 6-OMe (δ_H_ 4.12) to C-6, from 3-OMe (δ_H_ 3.58) to C-3, from 1-OH (δ_C_ 5.06) to C-1, C-2 and C-6 were assigned to the 1,4-dimethoxy-6-hydroxybenzene ring. The joint of indoline and benzene moieties has been proven with HMBC correlations (Figure 2d) from H-2’ to C-3 and C-4, and values of chemical shifts of C-3, C-4, C-2’ and C-3’. Thus, the structure of a tetracyclic system consists of indoline-benzofuran fragment was established. HMBC correlations from H-2” to C-2 indicated the structure of bis-indolebenzofuran-derivative.

A direct comparison of the NMR data of **6** with literature data for known varioloid D [29,30] revealed the close similarities, except the signals of C-1, C-2, C-4, C-6 and C-3” in **6**. These data proved the presence of a tetracyclic system formed via a reduction of C-2’ and C-3’ in indole moiety followed by cyclization at C-4 and C-2’. Thus, compound **6** has a planar structure 3-(1H-indol-3-yl)-1,4-dimethoxy-5a,6-dihydro-10bH-benzofuro [2,3-b]indole-2,10b-diol. Absolute configurations of stereocenters at C-2’ and C-3’ in **6** were determined based on a comparison of experimental ECD data of **6** with those for closely related known varioloid C [29,30]. Compound **6** was named asterriquinone F. It should be noted that a compound with this planar structure was once reported by Arai and Yamamoto [31] as the unnamed derivative of asterriquinone D. Nevertheless, these authors have not provided reliable structure elucidation evidence. The similarity of optical rotation values of asterriquinone F (6) and the unnamed compound from Arai and Yamamoto’s report (+47 and +39, respectively) may indicate an identity of these compounds. In addition, another Arai and Yamamoto’s paper proved an origin of asterriquinone F (**6**) via oxidation of asterriquinone D (**11**) [32].

Besides asterriquinone F (**6**), 10 known compounds were isolated from *A. terreus.* They were identified using NMR and MS analysis as asterriquinones A3 (**7**), B4 (**8**), C1 (**9**), C2 (**10**) and D (**11**) [33], questin (**12**) [34,35], 1,2,5-trihydroxy-7-methyl-9,10-antraquinone (**13**) [36], 4-hydroxy-3-(3-methylbut-2-enyl)benzaldehyde (**14**) [37,38], quadrone (**15**) [39] and 6β-hydroxyergosta-4,7,22-trien-3-on (**16**) [40]. It should be noted that antraquinone (**13**) was reported once as a byproduct in nataloe-emodin synthesis [36].

Unfortunately, compounds **13**–**16** were obtained in insufficient amounts and were not studied for biological activities.

### 2.2. Biological Activities of the Studied Compounds

The neuroprotective activity of the compounds in Parkinson’s disease (PD) in vitro models was investigated in murine malignant Neuro-2a cells, which are widely used for this purpose [41,42]. Firstly, all investigated compounds were tested on cytotoxicity against neuroblastoma Neuro-2a cells (Table 2).

Compounds **1**–**7** and **10**–**12** did not show any influences on Neuro-2a cell viability up to 100 µM. Quinones **8** and **9** were more effective in this assay and demonstrated cytotoxic activity with IC_50_ at 91.45 and 42.32 µM, respectively.

#### 2.2.1. 4-Hydroxyscytalone (1), 4-Hydroxy-6-Dehydroxyscytalone (2) and Demethylcitreoviranol (3)

**Figure 3:**
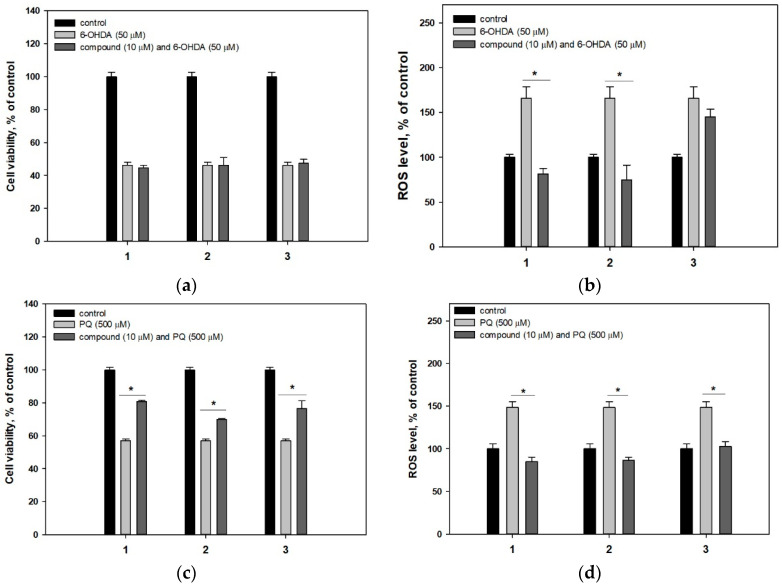

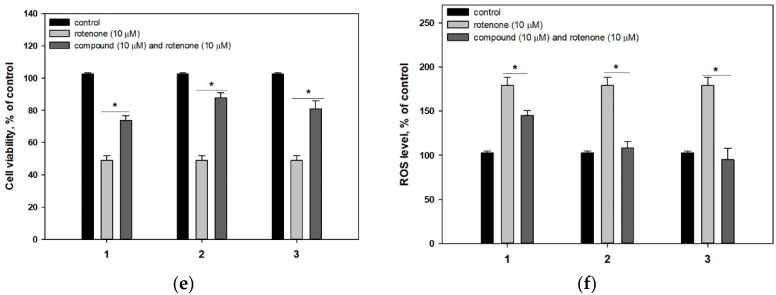
Influence of compounds **1**–**3** on viability of Neuro-2a cells treated with 6-OHDA (**a**), PQ (**c**) and rotenone (**e**). Influence of compounds **1**–**3** on ROS level in cells treated with 6-OHDA (**b**), PQ (**d**) and rotenone (**f**). * Differences are significant with *p* ≤ 0.05.

Compounds **1**–**3** did not affect viability of Neuro-2a cells incubated with 6-OHDA but they were significantly active in paraquat (PQ)- and rotenone-induced PD cell models (Figure 3). PQ decreased the Neuro-2a cell viability by 44% and compounds **1**–**3** increased the viability of PQ-treated cells by 41.8%, 22.8% and 34.3%, respectively (Figure 3c). Rotenone decreased the viability of Neuro-2a cells by 48% and compounds **1**–**3** increased the viability of rotenone-treated cells by 50.9%, 79.1% and 65.2%, respectively (Figure 3e).

Moreover, the influence of investigated compounds on the ROS level in neurotoxin-treated cells was tested. 6-OHDA, PQ and rotenone increased an intracellular ROS level by 66% (Figure 3b), 48% (Figure 3d) and 79% (Figure 3f), respectively. Compounds **1** and **2** decreased a ROS level in all used PD cell models (Figure 3b,d,f) whereas **3** was effective against ROS level enhancing in PQ- and rotenone-induced PD models only. At the same time, radical scavenging activity of **1**–**3** in cell-free assay was not significant (Table 2).

#### 2.2.2. 4-Hydroxy-3,6-Dimethyl-2-Pyrone (4) and Dihydroaspyrone (5)

4-Hydroxy-3,6-dimethyl-2-pyrone (**4**) and dihydroaspyrone (**5**) were not cytotoxic against neuroblastoma Neuro-2a cells up to 100 µM (Table 2).

**Figure 4:**
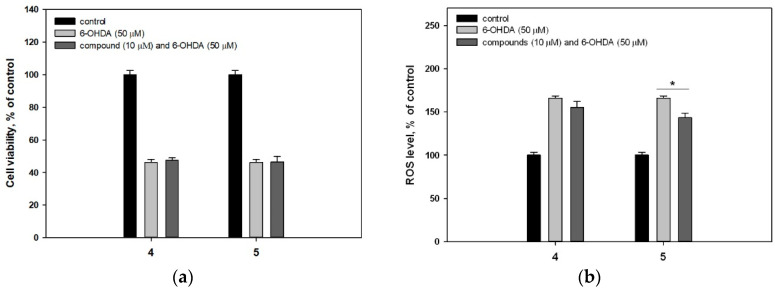

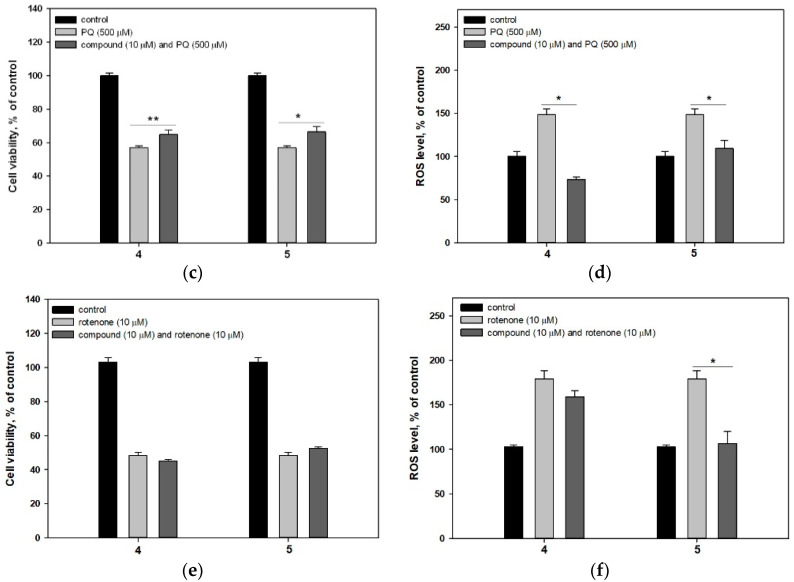
Influence of compounds **4** and **5** on viability of Neuro-2a cells treated with 6-OHDA (**a**), PQ (**c**) and rotenone (**e**). Influence of compounds **4** and **5** on the ROS level in cells treated with 6-OHDA (**b**), PQ (**d**) and rotenone (**f**). * Differences are significant with *p* ≤ 0.05. ** Differences are significant with *p* ≤ 0.10.

4-Hydroxy-3,6-dimethyl-2-pyrone (**4**) and dihydroaspyrone (**5**) did not demonstrate any protective effects against 6-OHDA (Figure 4a). Compound **5** statistically increased the viability of PQ-treated cells by 21.9% whereas 4-hydroxy-3,6-dimethyl-2-pyrone (**4**) had no effect on viability. These data are consistent with the effect of compounds **4** and **5** on the intracellular ROS level (Figure 4d). Pyrone **4** had almost no effect in this test, while **5** significantly reduced the ROS level in PQ-treated Neuro-2a cells.

Quite unexpectedly, pyrones **4** and **5** did not affect rotenone-treated Neuro-2a cell viability (Figure 4e) since they both significantly reduced the ROS level in these cells (Figure 4f). It was reported earlier, that dihydroaspyrone **5** showed radical scavenging activity with half-maximal effective concentration at 500 μM. In our study both pyrones did not show significant antiradical activity (Table 2).

#### 2.2.3. Asterriquinones (**6–11**) and Questin (**12**)

The cytotoxic activity of compounds **6**–**12** toward Neuro-2a cells were studied and asterriquinones B4 (**8**) and C1 (**9**) and questin (**12**) showed half-maximal toxic effect at 91.45, 42.32 and 105.36 µM (Table 2). So, compounds **6**–**12** were investigated in PD in vitro models at a nontoxic concentration of 1 µM.

**Figure 5:**
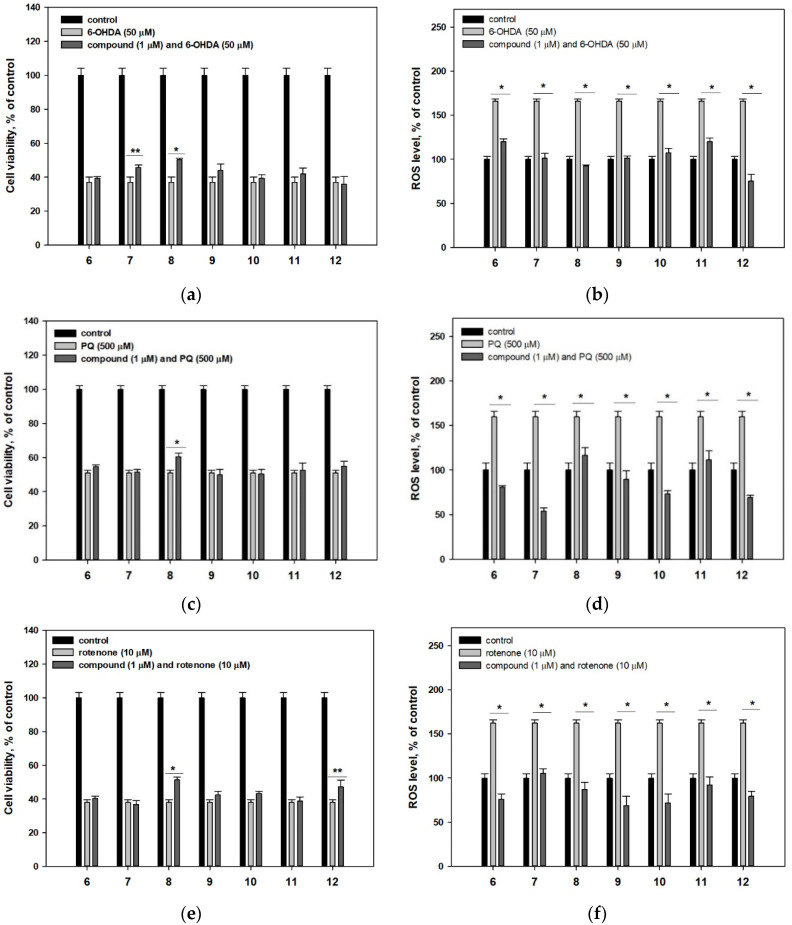
Influence of compounds **6**–**12** on viability of Neuro-2a cells treated with 6-OHDA (**a**), PQ (**c**) and rotenone (**e**). Influence of compounds **6**–**12** on ROS level in Neuro-2a cells treated with 6-OHDA (**b**), PQ (**d**) and rotenone (**f**). * Differences are significant with *p* ≤ 0.05. ** Differences are significant with *p* ≤ 0.10.

Asterriquinone A3 (**7**) showed a weak cytoprotective activity in 6-OHDA-induced PD cell model (Figure 5a). Additionally, asterriquinone B4 (**8**) protected Neuro-2a cells against all used neurotoxins and increased cell viability by 36.3% (Figure 5a), 18.4% (Figure 5c) and 34.8% (Figure 5e). Other investigated asterriquinones **6** and **9**–**11** were inactive in these assays. Nevertheless, all investigated asterriquinones decreased the ROS level in 6-OHDA-, PQ- and rotenone-treated Neuro-2a cells (Figure 5b,d,f).

Questin (**12**) showed moderate cytoprotective activity in the rotenone-induced PD cell model and increased the viability of cells by only 23.7% (Figure 5e). At the same time, **12** was inactive against 6-OHDA and PQ neurotoxicity while the significant in vitro anti-ROS activity of **12** was found (Figure 5b,d,f). Earlier DPPH-radical scavenging activity has been reported for questin (**12**) however no exact data have been published [44]. In our investigation **12** did not show any significant DPPH radical scavenging up to 100 μM (Table 2).

## 3. Discussion

4-Hydroxyscytalone (**1**) is a well-known metabolite intermediate of pentaketide pathway of melanin biosynthesis [45]. Melanin occurs in the cell walls of many fungi. Melanized fungal cells survive desiccation and ultraviolet irradiation notably better than their hyaline counterparts. The ability of certain fungi to produce melanin also appears to be an important determinant of pathogenicity. Known intermediates in the pentaketide melanin pathway include 1,3,6,8-tetrahydroxynaphthalene (1,3,6,8-THN) formed via pentaketide precursor cyclization, scytalone, 1,3,8-trihydroxynaphthalene (1,3,8-THN), vermelone and l,8-dihydroxynaphthalene (DHN). 4-Hydroxyscytalone (**1**) is formed via hydrogenation of 1,2,4,5,7-pentahydroxynaphtalene (1,2,4,5,7-PHN), which is a redox product of 1,3,6,8-THN. 4-Hydroxy-6-dehydroxyscytalone (**2**) also known as 3,4,8-trihydroxynaphtalene (3,4,8-THN) may be produced via hydrogenation and dehydration of 1,2,4,5,7-PHN or from scytalone via its dehydration and subsequent oxygenation and hydrogenation [45].

Pentaketides **1** and **2** were isolated from fungi several times [46,47], and described as phytotoxic [48,49] and antinematodal agents [50]. Cytotoxicity of 4-hydroxyscytalone (**1**) against four tumor cell lines, including MCF-7, HepG-2, NCI-H460 and SF-268 and some others was investigated, but no activity was shown [27,51,52]. Moreover, a weak antimicrobial activity against *Escherichia coli* and *Bacillus subtilis* was reported for **1** [53]. Despite a relationship of **1** and **2** with melanin, their protective properties toward PQ and rotenone cytotoxicity are described for the first time.

Demethylcitreoviranol (**3**) have been reported only twice [25,54]. It was investigated in the ARE luciferase assay and showed a weak inducing of the Nrf2-ARE pathway, which may suppress of oxidative genes and oxidative stress induced neurodegenerative diseases and carcinogenesis [54]. This has been confirmed in PQ- and rotenone-induced PD cell models. 

The structural differences between **1** and **3** did not affect their neuroprotective activity. Probably, 1-naphtalenone or geometrically similar isocoumarine core is of great importance for the neuroprotective effect of these compounds against PQ and rotenone toxicity.

The lack of protective effect of **1**–**3** against the 6-OHDA-induced neuronal damage is apparently associated with a different mechanism of 6-OHDA, PQ and rotenone actions. All these neurotoxins cause oxidative stress and enhance ROS accumulation in cells. 6-OHDA induces oxidative stress both during its autoxidation to *p*-quinone and, also, during one-electron reduction of *p*-quinone to *p*-semiquinone, catalyzed by flavoenzymes that transfer one electron. PQ causes generation of intracellular free radicals via reducing of the divalent paraquat ion (PQ2+) to monovalent paraquat ion (PQ+) by NADPH-oxidase of mitochondrial complex I and reestablishing a new redox reaction by PQ+. In turn, rotenone was reported as a direct inhibitor of mitochondrial complex I. Thus, neuroprotective activity of compounds **1**-**3** is due not so much to the ROS scavenging (since it decreased intracellular ROS level in all three PD models), but also to the influence on some other aspects of PQ and rotenone neurotoxicity.

4-Hydroxy-3,6-dimethyl-2-pyrone (**4**) earlier was tested for antibacterial activities (methicillin-resistant *Staphylococcus aureus* (MRSA), *S. aureus*, *Enterococcus faecalis* and *Acinetobacter baumannii*, *E. coli* and *Klebsiella pneumonia*) and cytotoxic activities against K562, BEL-7042, SGC-7901, A-549 and Hela cell lines and was ineffective [55]. In addition, it demonstrated a weak cytotoxicity against murine lymphoma L5178Y cell line [56]. As it was reported by Abe at al. **4** shows DPPH radical scavenging activity via donation of a hydrogen atom to DPPH radical and forming adduct with DPPH radical [57]. We suggest this mechanism may be realized in PQ-treated Neuro-2a cells. Differences between the mechanism of ROS generation by 6-OHDA, PQ and rotenone resulted in this fact that **4** reduced the ROS level in PQ-treated cells only. 

Dihydroaspyrone (**5**) earlier showed a weak cytotoxicity against HeLa [58], while it did not show cytotoxicity against a number of other cell lines [59,60] and antimicrobial activity against several microbial pathogens [59,61]. In present study **5** reduced ROS level in all neurotoxin-treated Neuro-2a cells but it statistically increased viability of PQ-treated cells only. Additional hydroxylation of **5** compared with **4** may play a key role for realizing of anti-ROS activity of **5** in neurotoxin-induced PD cell models.

It was earlier published that synthetic analogues of the asterriquinones, 1H5 and 5E5, have activated TrkA tropomyosin receptor kinase A directly in the cells and protected differentiated PC12 cells (rat adrenal pheochromocytomas) or contributed to the differentiation of neurons [62,63]. 

In our investigation only asterriquinone B4 (**8**) showed a significant neuroprotective activity among studied quinones. Probably, C-2 reverse-prenylated indole is the key moiety for neuroprotective properties of asterriquinones. Thus, asterriquinones A3 (**7**) and C1 (**9**) with one C-2 reverse-prenylated indole moiety was much less active compared with symmetric asterriquinone B4 (**8**). Other asterriquinones without this moiety were inactive. Recently we reported that other C-2 reverse-prenylated indole containing fungal metabolites increased rotenone- and PQ-treated Neuro-2a cells viability [16].

Thus, neuroprotective effects of asterriquinone B4 (**8**) and questin (**12**) from *A. terreus* in toxin-induced PD cell models were found at the first time. Earlier some butenolides and butyrolactone I from tropical strains *A. terreus* were reported as antineuroinflammatory compounds [64,65]. Moreover, butyrolactones I and VII from *A. terreus* exhibited protective activity against the glutamate-induced excitotoxicity [66]. Butyrolactone aspernolide F from endophytic strain *A. terreus* shown protective activity against doxorubicin-induced cardiotoxicity [67].

Metabolism of marine fungi is focused on survival in difficult highly competitive environments. For this reason, antimicrobial and cytotoxic fungal metabolites were the main goal of researchers during many years [68]. At the same time, the presence of cytoprotective compounds also should help fungi to successfully compete with bacteria in microbial communities. However, the cytoprotective potential of marine fungi is unappreciated today and these research are quite promising.

## 4. Materials and Methods 

### 4.1. General

Optical rotations were measured on a Perkin-Elmer 343 polarimeter (Perkin Elmer, Waltham, MA, USA). UV spectra were recorded on a Specord UV VIS spectrometer (Carl Zeiss, Jena, Germany) in MeOH. CD spectra were measured with a Chirascan-Plus CD spectrometer (Applied Photophysics, Leatherhead, United Kingdom) in MeOH. NMR spectra were recorded in CDCl_3_, acetone-d_6_ and DMSO-d_6_ with Bruker DPX-500 (Bruker BioSpin GmbH, Rheinstetten, Germany) or Bruker DRX-700 (Bruker BioSpin GmbH, Rheinstetten, Germany) spectrometers using TMS as an internal standard. HR-ESI-MS spectra were measured on a Maxis impact mass spectrometer (Bruker Daltonics GmbH, Rheinstetten, Germany).

Low-pressure liquid column chromatography was performed using silica gel (50/100 μm, Imid, Russia) and Sephadex^TM^ LH-20 (GE Healthcare, Uppsala, Sweden). Plates (4.5 cm × 6.0 cm) precoated with silica gel (5–17 μm, Imid) and silica gel 60 RP-18 F_254_S (20 cm × 20 cm, Merck KGaA, Darmstadt, Germany) were used for thin-layer chromatography. Preparative HPLC was carried out with a Shimadzu LC-20 chromatograph (Shimadzu USA Manufacturing, Canby, OR, USA) using YMC ODS-AM (YMC Co., Ishikawa, Japan; 5 µm, 10 mm × 250 mm) and YMC SIL (YMC Co., Ishikawa, Japan) (5 µm, 10 mm × 250 mm) columns with a Shimadzu RID-20A refractometer (Shimadzu Corporation, Kyoto, Japan) and with an Agilent 1260 Infinity II chromatograph (Agilent Technologies, Waldbronn, Germany) using a Supelco Discovery C-18 column (Sigma-Aldrich Co. LLC, Bellefonte, PA, USA) (5 µm, 4.6 mm × 250 mm) with an Agilent 1260 Infinity II UV detector (Agilent Technologies, Waldbronn, Germany).

### 4.2. Fungal Strains

The strain *Penicillium* sp. KMM 4672 was isolated from a brown alga *Padina* sp. (Van Phong Bay, South China Sea, Vietnam) on malt extract agar, and identified on the basis of morphological and molecular features, as described earlier [69].

The strain *Aspergillus flocculosus* was isolated from a sediment sample (Nha Trang Bay, South China Sea, Vietnam) by inoculating on the modified Sabouraud medium and on the basis of morphological and molecular features, as described earlier [17].

The strain of *Aspergillus terreus* was isolated from leaves of the unidentified mangrove tree collected in Khanh Hoa province (Vietnam, South China Sea) by inoculating on the modified Sabouraud medium (peptone 10 g, glucose 20 g, agar 18 g, natural sea water 1000 mL, penicillin 1.5 g and streptomycin 1.5 g, pH 6.0–7.0). The fungus was identified according to a molecular biological protocol by DNA amplification and sequencing of the ITS region (GenBank accession number MN788658.1). BLAST search results indicated that the sequence was 98.06% identical (858/875 bp) with the sequence of *Aspergillus terreus* strain DTO 403-C9 (GenBank accession number MT316343.1). The strain is stored at the collection of microorganisms of the Nha Trang Institute of Technology and Research Application VAST (Nha Trang, Vietnam) under the code LM.5.2

### 4.3. Cultivation of the Fungi

All the fungal strains were cultured at room temperature for three weeks in 60 × 500 mL Erlenmeyer flasks each containing rice (20.0 g), yeast extract (20.0 mg), KH_2_PO_4_ (10 mg) and natural seawater (40 mL).

### 4.4. Extraction and Isolation

The main part of the isolation procedures of compounds from *Penicillium* sp. KMM 4672 was described in a previous paper [22]. The *n*-hexane–EtOAc (85:15, 380.0 mg) fraction was separated by a sephadex LH-20 column (80 cm × 2 cm) with CHCI_3_-EtOH (1:1) and then purified by HPLC on an YMC ODS-AM column, eluting with MeCN–H_2_O (45:55) to yield **2** (5.6 mg). The *n*-hexane–EtOAc (80:20, 150.0 mg) fraction was separated by a sephadex LH-20 column (80 cm × 2 cm) with CHCI_3_-EtOH (1:1) and then purified by HPLC on an YMC ODS-AM column, eluting with MeOH–H_2_O (65:35) and MeCN–H_2_O (45:55) to yield **3** (2.0 mg). The *n*-hexane–EtOAc (70:30, 190.0 mg) fraction was separated by a sephadex LH-20 column (80 cm × 2 cm) with CHCI_3_-EtOH (1:1) and then purified by HPLC on an YMC ODS-AM column, eluting with MeOH–H_2_O (65:35) to yield **1** (1.9 mg). The isolation of compound **4** was described in a previous paper [22].

The isolation of compound **5** from *Aspergillus flocculosus* was described in a previous paper [18].

The fungal mycelia of *Aspergillus terreus* with the medium were extracted for 24 h with 12.0 L of EtOAc. Evaporation of the solvent under reduced pressure gave a dark brown oil (2.7 g). To this residue was added 150 mL of H_2_O-EtOH (4:1), and the mixture was thoroughly stirred to yield a suspension. The suspension was sequentially extracted with hexane (100 mL × 3), EtOAc (150 mL × 3) and n-BuOH (150 mL × 2). The EtOAc fraction was concentrated under reduced pressure to give a dry residue (1.6 g), which was separated on a silica gel column (20.0 cm × 4 cm) eluted with a hexane-EtOAc gradient (1:0→0:1). The hexane-EtOAc fraction AT-1-5 (95:5, 564.72 mg) was separated using Sephadex LH-20 eluting with chloroform and by HPLC on an YMC ODS-AM column in MeCN-H_2_O (70:30) to yield **13** (1.00 mg) and **14** (1.30 mg). The hexane-EtOAc fraction AT-1-9 (95:5, 38.96 mg) was separated using Sephadex LH-20 eluting with chloroform and subsequent HPLC separations on an YMC SIL column in EtOAc-n-hexane (25:75) and EtOAc-n-hexane (30:70), and on an YMC ODS-AM column in MeCN-H_2_O (90:10), to yield **7** (1.43 mg). The hexane-EtOAc fraction AT-1-19 (90:10, 47.70 mg) was separated sequentially on a column with Sephadex LH-20 in CHCl_3_ and by HPLC on an YMC-SIL column in EtOAc-n-hexane (20:80) and EtOAc-CHCl_3_ (5:95) to yield **8** (2.70 mg), **15** (1.94 mg) and **16** (0.70 mg). The hexane-EtOAc fraction AT-1-14 (90:10, 82.31 mg) was separated using Sephadex LH-20 eluting with chloroform to yield **12** (1.80 mg). The hexane-EtOAc fraction AT-1-26 (85:15, 135.00 mg) was separated using Sephadex LH-20 eluting with chloroform and purified by HPLC on an YMC ODS-AM column in MeCN-H_2_O (70:30) to yield **9** (2.50 mg). The hexane-EtOAc fraction AT-1-30 (85:15, 154.00 mg) was separated using Sephadex LH-20 eluting with chloroform and purified by HPLC on an YMC ODS-AM column in MeCN-H_2_O (70:30) to yield **10** (2.80 mg). The hexane-EtOAc fraction AT-1-105 (65:35, 267.18 mg) was separated using Sephadex LH-20 eluting with chloroform to yield **11** (9.90 mg). The hexane-EtOAc fraction AT-101-69 (75:25, 154 mg) was separated using Sephadex LH-20 eluting with chloroform and purified by HPLC on an YMC ODS-AM column in MeCN-H_2_O (75:25) and then on a Supelco C-18 column in MeOH-H_2_O gradient (1:1→1:0) to yield **6** (2.44 mg).

*(3S,4S)-4-hydroxyscytalone* (**1**): White powder; ${\left[\alpha \right]}_{D}^{20}$ +73° (c 0.04, MeOH); UV (MeOH) λ_max_ (logε) 313 (3.60), 283 (3.90), 234 (3.77), 216 (3.98); CD (0.71 mM, MeOH) λ_max_ (Δε) 195 (+0.77), 215 (−4.82), 241 (+0.52), 280 (+1.36), 315 (+0.13); ^1^H and ^13^C-NMR data, see Supplementary Data (Figures S1 and S2); HR-ESI-MS [M – H]^–^ 209.0457 (calcd for C_10_H_9_O_5_, 209.0455).

*(3S,4S)-4-hydroxy-6-dehydroxyscytalone* (**2**): White powder; ${\left[\alpha \right]}_{D}^{20}$ +29° (c 0.22, MeOH); UV (MeOH) λ_max_ (logε) 333 (3.45), 259 (3.82), 216 (4.15); CD (0.96 mM, MeOH) λ_max_ (Δε) 196 (+0.93), 215 (−8.59), 231 (+0.28), 259 (+3.20); ^1^H and ^13^C-NMR data, see Supplementary Data (Figures S4–S6); HR-ESI-MS [M – H]^–^ 193.0505 (calcd for C_10_H_9_O_4_, 193.0506).

*Demethylcitreoviranol* (**3**): White powder; ${\left[\alpha \right]}_{D}^{20}$ –47° (c 0.09, MeOH); UV (MeOH) λ_max_ (logε) 302 (3.56), 271 (3.85), 212 (4.11); CD (0.54 mM, MeOH) λ_max_ (Δε) 199 (–6.56), 236 (−0.96), 272 (–0.97), 306 (+0.75); ^1^H and ^13^C-NMR data, see Supplementary Data (Figures S8–S10); HR-ESI-MS [M + Na]^+^ 303.0836 (calcd for C_14_H_15_O_6_Na, 303.0839).

*Asterriquinone F* (**6**): White powder; ${\left[\alpha \right]}_{D}^{20}$ +47° (c 0.04, MeOH); UV (MeOH) λ_max_ (logε) 281 (4.19), 221 (4.80), 200 (4.79); CD (0.092 mM, MeOH) λ_max_ (Δε) 195 (+14.98), 201 (−9.35), 208 (+15.19), 230 (−38.81), 274 (−5.90), 319 (+18.21); ^1^H and ^13^C-NMR data, see Table 1 and Supplementary Data (Figures S12–S18); HR-ESI-MS [M + Na]^+^ 439.1257 (calcd for C_24_H_20_N_2_O_5_Na, 439.1264).

### 4.5. DPPH Radical Scavenger Assay

The DPPH radical scavenging activities of the compounds were tested as described [70].

The compounds were dissolved in MeOH, and the solutions (120 µL) were dispensed into wells of a 96-well microplate. In all, 30 µL of the DPPH (Sigma-Aldrich, Steinheim, Germany) solution in MeOH (7.5 × 10^−3^ M) was added to each well. The concentrations of the test compounds in the mixtures were 10 and 100 µM. The mixtures were shaken and left to stand for 30 min, and the absorbance of the resulting solutions was measured at 520 nm with a microplate reader MultiscanFC (ThermoScientific, Waltham, MA, USA). The concentration scavenging 50% of the DPPH radical (EC_50_) was calculated for each investigated compound.

### 4.6. Bioassays

#### 4.6.1. Cell Culture

The murine neuroblastoma cell line Neuro-2a was purchased from ATCC.

Neuro-2a cells were cultured in DMEM medium containing 10% fetal bovine serum (Biolot, St. Petersburg, Russia) and 1% penicillin/streptomycin (Biolot, St. Petersburg, Russia). Cells were incubated at 37 °C in a humidified atmosphere containing 5% (*v*/*v*) CO_2_ [71].

#### 4.6.2. Cytotoxicity Assay

The in vitro cytotoxicity of the individual substances was evaluated using an MTT (3-(4,5-dimethylthiazol-2-yl)-2,5-diphenyltetrazolium bromide) assay, which was performed according to the manufacturer’s instructions (Sigma-Aldrich, USA). Absorbance of the converted formazan was measured using a Multiskan FC plate photometer (Thermo Scientific, Waltham, MA, USA) at λ = 570 nm. The results were presented as percent of control data, and concentration of cell viability inhibition on 50% (IC_50_) was calculated [71].

#### 4.6.3. Neurotoxin-Induced Cell Models of Parkinson’s Disease

The neuroblastoma Neuro-2a line cells (1 × 10^4^ cells/well) were treated with the test compounds at concentrations of 1 and/or 10 µM for 1 h, and then the neurotoxins were added to the neuroblastoma cell suspensions [17]. Rotenone (Sigma-Aldrich, USA) was used at the concentration of 10 µM. Paraquat (Sigma-Aldrich, USA) was used at 500 µM. 6-Hydroxydopamine (Sigma-Aldrich, USA) was used at 50 µM. Cells incubated without neurotoxins and the test compounds and cells incubated with neurotoxins only were used as positive and negative controls, respectively. After 24 h of incubation, the cell viabilities were measured using the MTT method. The results are presented as the percent of positive control data.

#### 4.6.4. ROS Level Studying in Neurotoxin-Treated Neuro-2a Cells

The cells (1 × 10^4^ cells/well of a 96-well plate) were incubated with compound solutions (10 µM) during 1 h. Then, 6-OHDA/PQ/rotenone were added to cell suspension to resulting concentration of 50 µM, 500 µM and 10 µM respectively for incubation during 1 h. Cells incubated without neurotoxins and compounds and with neurotoxins alone were used as positive and negative controls, respectively. The 20 µL of 2,7-dichlorodihydrofluorescein diacetate solution (H_2_DCFDA, Molecular Probes, Eugene, OR, USA) was added to each well (10 µM, final concentration) and the plate was incubated for an additional 10 min at 37 °C. The intensity of dichlorofluorescein fluorescence was measured with PHERAstar FS plate reader (BMG Labtech, Ortenberg, Germany) at λ_ex_ = 485 nm and λ_em_ = 518 nm. The data were processed by MARS Data Analysis v. 3.01R2 (BMG Labtech, Germany). The results were presented as the percent of positive control data [71].

## Figures and Tables

**Table 1:** ^1^H and ^13^C NMR spectroscopic data (δ in ppm, CDCl_3_) for **6**.

Pos.	δ_C_, mult	δ_H_, (*J* in Hz)	HMBC	COSY
1	141.3, C			
2	116.7, C			
3	138.4, C			
4	144.9, C			
5	121.6, C			
6	139.1, C			
6-OMe	61.5, CH_3_	4.12, s	6	
3-OMe	60.4, CH_3_	3.58, s	3	
2’	104.6, CH	6.24, s	4, 5, 3’, 3a’, 7a’	
3’	90.3, C			
3a’	129.7, C			
4’	125.0, CH	7.78, d (7.5)	3’, 6’, 7’, 7a’	5’
5’	120.1, CH	6.88, t (7.5)	3a’, 4’, 7’	4’, 6’
6’	130.1, CH	7.18, t (7.8)	3a’, 4’, 7’, 7a’	5’, 7’
7’	109.9, CH	6.70, d (8.0)	3a’, 5’	6’
7a’	148.6, C			
1”	(NH)	8.44, brs	3”	2”
2”	124.8, CH	7.29, d (2.4)	2, 3”, 3a’’, 7a’’	3’’
3”	106.7, C			
3a’’	126.5, C			
4”	120.3, CH	7.47, d (8.1)	3”, 3a’’, 5”, 6”, 7”, 7a’’	5’’
5”	120.4, CH	7.14, t (7.5)	3a’’, 7”	4’’, 6’’
6”	122.7, CH	7.24, t (7.8)	4”, 7a’’	5’’, 7’’
7”	111.4, CH	7.43, d (8.1)	3a’’, 5”, 6”	6’’
7a’’	136.2, C			
1-OH		5.06, brs	1, 2, 6	

**Table 2:** Cytotoxicity and radical scavenging activities of compounds **1**–**12**.

Compounds	Cytotoxicity	DPPH Radical Scavenging	
	IC_50_, µM	100 µM % of MeOH	EC_50_, µM
**1**	>100	83.4 ± 1.2	>100
**2**	>100	84.8 ± 3.2	>100
**3**	>100	91.2 ± 1.8	>100
**4**	>100	87.2 ± 1.4	500 [43]
**5**	>100	95.2 ± 1.0	>100
**6**	>100	67.3 ± 3.9	>100
**7**	>100	88.9 ± 1.6	>100
**8**	91.45 ± 1.87	89.5 ± 0.9	>100
**9**	42.32 ± 1.45	82.4 ± 1.7	>100
**10**	>100	91.3 ± 1.1	>100
**11**	>100	85.6 ± 1.3	>100
**12**	>100	87.7 ± 2.4	>100

## Supplementary Materials

The following are available online at https://www.mdpi.com/1660-3397/18/12/608/s1. Figures S1, S2, S4–S6, S8–S10, S12–S18, S20–S39: NMR spectra of compounds **1**–**16**, Figures S3, S7, S11, S19: CD spectra of compounds **1**–**3** and **6**, Tables S1–S4: NMR data for compounds **7**–**14**.
