# Supplementary material for: Neuroprotective Metabolites from Vietnamese Marine Derived Fungi of Aspergillus and Penicillium Genera
Source: Mar Drugs. 2020 Nov 30;18(12):608. doi: 10.3390/md18120608 (PMC7760690; doi:10.3390/md18120608)

SUPPLEMENTARY MATERIAL

# Neuroprotective Metabolites from Vietnamese Marine Derived Fungi of Aspergillus and Penicillium Genera

**Elena V. Girich[1], Anton N. Yurchenko[1*], Olga F. Smetanina[1], Phan Thi Hoai Trinh[2], Ngo Thi Duy Ngoc[2], Mikhail V. Pivkin[1], Roman S. Popov[1], Evgeny A. Pislyagin[1], Ekaterina S. Menchinskaya[1], Ekaterina A. Chingizova[1], Shamil Sh. Afiyatullov[1] and Ekaterina A. Yurchenko[1]**

[1] G.B. Elyakov Pacific Institute of Bioorganic Chemistry, Far Eastern Branch of Russia Academy of Sciences, Vladivostok, Russia;

[2] Department of marine biotechnology, Nhatrang Institute of Technology Research and Application, Vietnam Academy of Science and Technology, Nha Trang, Vietnam;

* Correspondence: yurchant@ya.ru; Tel.: +7-423-231-1168 (A.N.Y.)

**Abstract:** Low molecular weight secondary metabolites of marine fungi *Aspergillus flocculosus*, *Aspergillus terreus* and *Penicillium* sp. from Van Phong and Nha Trang Bays (Vietnam) were studied and a number of polyketides, bis-indole quinones and terpenoids were isolated. The structures of the isolated compounds were determined by 1D and 2D NMR and HRESIMS techniques. Stereochemistry of some compounds was established based on ECD data. A chemical structure of asterriquinone F (**6**) was thoroughly described for the first time. Anthraquinone (**13**) was firstly obtained from natural source. Neuroprotective influences of the isolated compounds against 6-OHDA, paraquat and rotenone toxicity were investigated. 4-Hydroxyscytalone (**1**), 4-hydroxy-6-dehydroxyscytalone (**2**) and demethylcitreoviranol (**3**) have shown significant increasing of paraquat- and rotenone-treated Neuro-2a cell viability and anti-ROS activity.

**Keywords:** *Aspergillus terreus*; *Aspergillus flocculosus*; *Penicillium* sp.; marine-derived fungi; South China Sea; secondary metabolites; asterriquinones; polyketides; neuroprotective activity; reactive oxygen species

**Content**


Table S1. $^{13}C$ NMR ($\delta$ in ppm) data for asterriquinones A3 (**7**), B4 (**8**), C1 (**9**), C2 (**10**) and D (**11**) ........ 4

Table S2. $^{1}H$ NMR data ($\delta$ in ppm, $J$ in Hz) for asterriquinones A3(**7**), B4 (**8**), C1 (**9**), C2 (**10**) and D (**11**) ........ 5

Table S3. $^{1}H$ and $^{13}C$ NMR data (700 MHz, DMSO-$d_6$, $\delta$ in ppm) for 1,2,5-trihydroxy-7-methyl-9,10-antraquinone (**13**)........ 6

Table S4. $^{1}H$ and $^{13}C$ NMR data (700 MHz, $CDCl_3$, $\delta$ in ppm) for 4-hydroxy-3-(3-methylbut-2-enyl)benzaldehyde (**14**) ........ 6

**Figure S1. $^{1}H$ NMR (700 MHz, $CDCl_3$) spectrum of 4-hydroxyscytalone (1)**........ 7

**Figure S2. $^{13}C$ NMR (700 MHz, $CDCl_3$) spectrum of 4-hydroxyscytalone (1)**........ 9

**Figure S3. CD spectrum of 4-hydroxyscytalone (1)** ........ 10

**Figure S4. $^{1}H$ NMR (700 MHz, $CDCl_3$) spectrum of 4-hydroxy-6-dehydroxyscytalone (2)** ........ 11

**Figure S5. $^{13}C$ NMR (700 MHz, $CDCl_3$) spectrum of 4-hydroxy-6-dehydroxyscytalone (2)** ........ 13

**Figure S6. DEPT NMR (700 MHz, $CDCl_3$) spectrum of 4-hydroxy-6-dehydroxyscytalone (2)** ........ 14

**Figure S7. CD spectrum of 4-hydroxy-6-dehydroxyscytalone (2)**........ 15

**Figure S8. $^{1}H$ NMR (700 MHz, $CDCl_3$) spectrum of demethylcitreoviranol (3)** ........ 16

**Figure S9. $^{13}C$ NMR (700 MHz, $CDCl_3$) spectrum of demethylcitreoviranol (3)** ........ 17

**Figure S10. DEPT NMR (700 MHz, $CDCl_3$) spectrum of demethylcitreoviranol (3)** ........ 19

**Figure S11. CD spectrum of demethylcitreoviranol (3)**........ 20

**Figure S12. $^{1}H$ NMR (700 MHz, $CDCl_3$) spectrum of asterriquinone F (6)**........ 21

**Figure S13. $^{13}C$ NMR (176 MHz, $CDCl_3$) spectrum of asterriquinone F (6)**........ 23

**Figure S14. DEPT (176 MHz, $CDCl_3$) spectrum of asterriquinone F (6)**........ 24

**Figure S15. HSQC (176 MHz, $CDCl_3$) spectrum of asterriquinone F (6)** ........ 25

**Figure S16. HMBC (176 MHz, $CDCl_3$) spectrum of Asterriquinone F (6)** ........ 26

**Figure S17. COSY (176 MHz, $CDCl_3$) spectrum of asterriquinone F (6)**........ 27

**Figure S18. ROESY (176 MHz, $CDCl_3$) spectrum of asterriquinone F (6)**........ 28

**Figure S19. CD spectrum of asterriquinone F (6)** ........ 29

**Figure S20. $^{1}H$ NMR (700 MHz, $CDCl_3$) spectrum of asterriquinone A3 (7)**........ 30

**Figure S21. $^{13}C$ NMR (176 MHz, $CDCl_3$) spectrum of asterriquinone A3 (7)**........ 31

**Figure S22. $^{1}H$ NMR (500 MHz, $CDCl_3$) spectrum of asterriquinone B4 (8)** ........ 32

**Figure S23. $^{13}C$ NMR (176 MHz, $CDCl_3$) spectrum of asterriquinone B4 (8)** ........ 33

**Figure S24.** $^{1}$**H NMR (500 MHz,** $CDCl_3$**) spectrum of asterriquinone C1 (9)** ......... 34

**Figure S25.** $^{13}$**C NMR (125 MHz,** $CDCl_3$**) spectrum of asterriquinone C1 (9)** ........ 35

**Figure S26.** $^{1}$**H NMR (700 MHz,** $CDCl_3$**) spectrum of asterriquinone C2 (10)** ....... 36

**Figure S27.** $^{13}$**C NMR (176 MHz,** $CDCl_3$**) spectrum of asterriquinone C2 (10)** ...... 37

**Figure S28.** $^{1}$**H NMR (700 MHz, Acetone-*d*$_6$) spectrum of asterriquinone D (11)** .. 38

**Figure S29.** $^{13}$**C NMR (176 MHz, Acetone-*d*$_6$) spectrum of asterriquinone D (11)**. 39

**Figure S30.** $^{1}$**H NMR (700 MHz, DMSO-*d*$_6$) spectrum of 1,2,5-trihydroxy-7-methyl-9,10-anthraquinone (13)** ........................................................................................ 40

**Figure S31.** $^{13}$**C NMR (176 MHz, DMSO-*d*$_6$) spectrum of 1,2,5-trihydroxy-7-methyl-9,10-anthraquinone (13)** ........................................................................ 41

**Figure S32.** $^{1}$**H NMR (700 MHz,** $CDCl_3$**) spectrum of 4-hydroxy-3-(3-methylbut-2-enyl)benzaldehyde (14)** ........................................................................................ 42

**Figure S33.** $^{13}$**C NMR (176 MHz,** $CDCl_3$**) spectrum of 4-hydroxy-3-(3-methylbut-2-enyl)benzaldehyde (14)** ........................................................................................ 43

**Figure S34.** $^{1}$**H NMR (500 MHz, MeOD-*d*$_3$) spectrum of questin (12)** .................... 44

**Figure S35.** $^{13}$**C NMR (125 MHz, MeOD-*d*$_3$) spectrum of questin (12)** .................. 45

**Figure S36.** $^{1}$**H NMR (500 MHz, MeOD-*d*$_3$) spectrum of quadrone (15)** ................. 46

**Figure S37.** $^{13}$**C NMR (125 MHz, MeOD-*d*$_3$) spectrum of quadrone (15)** ................ 47

**Figure S38.** $^{1}$**H NMR (500 MHz,** $CDCl_3$**) spectrum of 6β-hydroxyergosta-4,7,22-trien-3-on (16)** ........................................................................................................ 48

**Figure S39.** $^{13}$**C NMR (125 MHz,** $CDCl_3$**) spectrum of 6β-hydroxyergosta-4,7,22-trien-3-on (16)** ........................................................................................................ 49

Table S1. $^{13}$C NMR ($\delta$ in ppm) data for asterriquinones A3 (**7**), B4 (**8**), C1 (**9**), C2 (**10**) and D (**11**)

| Atom | **7***  | **8*** | **9*** | **10*** | **11**** |
|---|---|---|---|---|---|
| 1 | 184.0, C | 183.70, C | 184.0, C | 183.8, C | 185.2, C |
| 2 | 120.7, C | 120.7, C | 120.7, C | 120.7, C | 130.2, C |
| 3 | 156.2, C | 156.7, C | 156.2, C | 153.9, C | 155.4, C |
| 4 | 183.8, C | 183.68, C | 183.8, C | 183.8, C | 185.2, C |
| 5 | 122.2, C | 120.66, C | 126.7, C | 120.66, C | 130.2, C |
| 6 | 153.9, C | 156.8, C | 154.4, C | 153.9, C | 155.4, C |
| 2′ | 142.1, C | 142.1, C | 142.1, C | 127.4, CH | 130.1, CH |
| 3′ | 101.8, C | 101.84, C | 101.7, C | 105.7, C | 106.7, C |
| 3a′ | 129.9, C | 129.9, C | 129.9, C | 126.7, C | 128.7, C |
| 4′ | 122.1, CH | 118.7, CH | 118.7, CH | 119.4, CH | 123.0, CH |
| 5′ | 120.3, CH | 120.2, CH | 120.3, CH | 120.7, CH | 121.2, CH |
| 6′ | 118.7, CH | 122.1, CH | 122.1, CH | 122.21, CH | 123.2, CH |
| 7′ | 110.6, CH | 110.6, CH | 110.6, CH | 124.1, C | 113.1, CH |
| 7a′ | 134.5, C | 134.5, C | 134.5, C | 135.0, C | 137.8, C |
| 8′ | 39.3, C | 39.3, C | 39.3, C | 30.8, $CH_2$ | |
| 9′ | 27.0, $CH_3$ | 27.1, $CH_3$ | 27.1, $CH_3$ | 122.21, CH | |
| 10′ | 26.8, $CH_3$ | 27.0, $CH_3$ | 26.8, $CH_3$ | 133.6, C | |
| 11′ | 145.5, CH | 145.50, CH | 145.4, CH | 18.0, $CH_3$ | |
| 12′ | 112.3, $CH_2$ | 112.2, $CH_2$ | 112.3, $CH_2$ | 25.7, $CH_3$ | |
| 2″ | 129.2, CH | 141.9, C | 127.8, CH | 121.5, CH | 130.1, CH |
| 3″ | 103.4, C | 101.78, C | 105.5, C | 105.9, C | 106.7, C |
| 3a″ | 128.7, C | 129.7, C | 126.7, C | 126.6, C | 128.7, C |
| 4″ | 121.1, CH | 118.4, CH | 121.4, CH | 127.8, CH | 123.0, CH |
| 5″ | 119.9, CH | 120.3, CH | 120.5, CH | 120.6, CH | 121.2, CH |
| 6″ | 121.7, CH | 122.06, CH | 122.4, CH | 122.5, CH | 123.2, CH |
| 7″ | 114.0, CH | 110.7, CH | 111.3, CH | 111.3, CH | 113.1, CH |
| 7a″ | 135.3, C | 134.51, C | 135.6, C | 135.7, C | 137.8, C |
| 8″ | 39.3, C | 39.3, C | | | |
| 9″ | 28.03, $CH_3$ | 26.8, $CH_3$ | | | |
| 10″ | 28.01, $CH_3$ | 26.8, $CH_3$ | | | |
| 11″ | 143.8, CH | 145.52, CH | | | |
| 12″ | 113.9, $CH_2$ | 112.4, $CH_2$ | | | |
| 3-OMe | 60.1, $CH_3$ | 60.1, $CH_3$ | 60.0, $CH_3$ | 60.65, $CH_3$ | 61.5, $CH_3$ |
| 6-OMe | 60.6, $CH_3$ | 61.2, $CH_3$ | 60.7, $CH_3$ | 60.68, $CH_3$ | 61.5, $CH_3$ |

* - data were obtained in $CDCl_3$, 125 MHz; ** - data were obtained in acetone-$d_6$, 176 MHz

Table S2. $^{1}$H NMR data ($\delta$ in ppm, $J$ in Hz) for asterriquinones A3(**7**), B4 (**8**), C1 (**9**), C2 (**10**) and D (**11**)

| Atom | **7*** | **8*** | **9*** | **10*** | **11**** |
|---|---|---|---|---|---|
| 1′(NH) | 8.11, brs | 8.13, brs | 8.14, brs | 8.53, brs | |
| 2′ | | | | 7.59, d (2.8) | 7.69, s |
| 4′ | 7.164, m | 7.32, d (7.5) | 7.29, d (7.9) | 7.44, d (4.2) | 7.54, d (8.0) |
| 5′ | 7.09, t (7.45) | 7.12, t (7.4) | 7.09, t (7.5) | 7.12, t (7.5) | 7.09, t (7.5) |
| 6′ | 7.29, d (7.9) | 7.17, td (7.4, 3.8) | 7.33, d (8.1) | 7.05, d (7.2) | 7.16, t (7.5) |
| 7′ | 7.33, d (8.1) | 7.33, d (7.6) | 7.33, d (8.1) | | 7.49, d (8.1) |
| 8′ | | | | 3.62, brs<br>3.61, brs | |
| 9′ | 1.51, s | 1.50, brs | 1.51, s | 5.46, t (7.3) | |
| 10′ | 1.50, s | 1.50, brs | 1.50, s | | |
| 11′ | 6.11, dd (17.4, 10.5) | 6.11, t (10.7) | 6.11, dd (10.5, 6.9) | 1.85, s | |
| 12′ | 5.15, d (10.5)<br>5.20, d (17.4) | 5.18, d (10.5)<br>5.24, d (10.7) | 5.15, d (10.5)<br>5.20, d (6.9) | 1.81, s | |
| 1″ | | NH, 8.11, brs | NH, 8.56, brs | NH, 8.51, brs | |
| 2″ | 7.74, s | | 7.60, s | 7.60, brs | 7.69, s |
| 4″ | | 7.22, d (7.9) | 7.58, d (8.0) | 7.62, d (2.7) | 7.54, d (8.0) |
| 5″ | 7.155, m | 7.09, t (7.5) | 7.19, m | 7.19, t (7.5) | 7.09, t (7.5) |
| 6″ | 7.56, dd (6.9, 1.9) | 7.17, td (7.4, 3.8) | 7.24, d (7.6) | 7.25, d (7.7) | 7.16, t (7.5) |
| 7″ | 7.57, dd 7.4, 1.5) | 7.33, d (7.6) | 7.43, d (8.1) | 7.43, d (4.2) | 7.49, d (8.1) |
| 9″ | 1.84, s | 1.53, s | | | |
| 10″ | 1.84, s | 1.53, s | | | |
| 11″ | 6.22, dd (17.4, 10.5) | 6.14, t (10.7) | | | |
| 12″ | 5.27, d (10.7)<br>5.24, d (17.4) | 5.13, d (10.5)<br>5.21, t (16.7) | | | |
| 3-OMe | 3.80, s | 3.70, brs | 3.70, s | 3.786, brs | 3.82, s |
| 6-OMe | 3.69, s | 3.70, brs | 3.81, s | 3.793, brs | 3.82, s |

* - data were obtained in $CDCl_3$, 500 MHz; ** - data were obtained in acetone-$d_6$, 700 MHz

Table S3. $^1$H and $^{13}$C NMR data (700 MHz, DMSO-$d_6$, $\delta$ in ppm) for 1,2,5-trihydroxy-7-methyl-9,10-antraquinone (**13**)

| Atom | $^{13}$C, mult | $^1$H, $J$ in Hz |
|---|---|---|
| 1 | 164.4, C | |
| 2 | 165.5, C | |
| 3 | 107.9, CH | 6.60, d (2.5) |
| 4 | 108.9, CH | 7.14, d (2.5) |
| 4a | 108.7, C | |
| 5 | 161.3, C | |
| 6 | 124.1, CH | 7.18, brs |
| 7 | 148.2, C | |
| 8 | 120.4, CH | 7.52, brs |
| 8a | 135.1, C | |
| 9 | 189.7, C | |
| 9a | 132.8, C | |
| 10 | 181.4, C | |
| 10a | 113.4, C | |
| 7-Me | 21.4, $CH_3$ | 2.42, s |
| 1-OH | | 12.09, brs |
| 2-OH | | 11.33, brs |
| 5-OH | | 12.02, brs |

Table S4. $^1$H and $^{13}$C NMR data (700 MHz, $CDCl_3$, $\delta$ in ppm) for 4-hydroxy-3-(3-methylbut-2-enyl)benzaldehyde (**14**)

| Atom | $^{13}$C, mult | $^1$H, $J$ in Hz |
|---|---|---|
| 1 | 130.1, C | |
| 2 | 131.8, CH | 7.67, s |
| 3 | 127.6, C | |
| 4 | 160.0, C | |
| 5 | 116.2, CH | 6.91, d (7.9) |
| 6 | 130.5, CH | 7.66, d (2.2) |
| 1′ | 29.5, $CH_2$ | 3.42, s<br>3.41, s |
| 2′ | 120.6, CH | 5.33, t (7.4) |
| 3′ | 136.1, C | |
| 4′ | 25.8, $CH_3$ | 1.80, s |
| 5′ | 179, $CH_3$ | 1.79, s |
| 1″ | 191.1, CH | 9.85, s |
| 4-OH | | 5.83, brs |

**Figure S1. $^{1}H$ NMR (500 MHz, DMSO-$d_6$) spectrum of 4-hydroxyscytalone (1)**

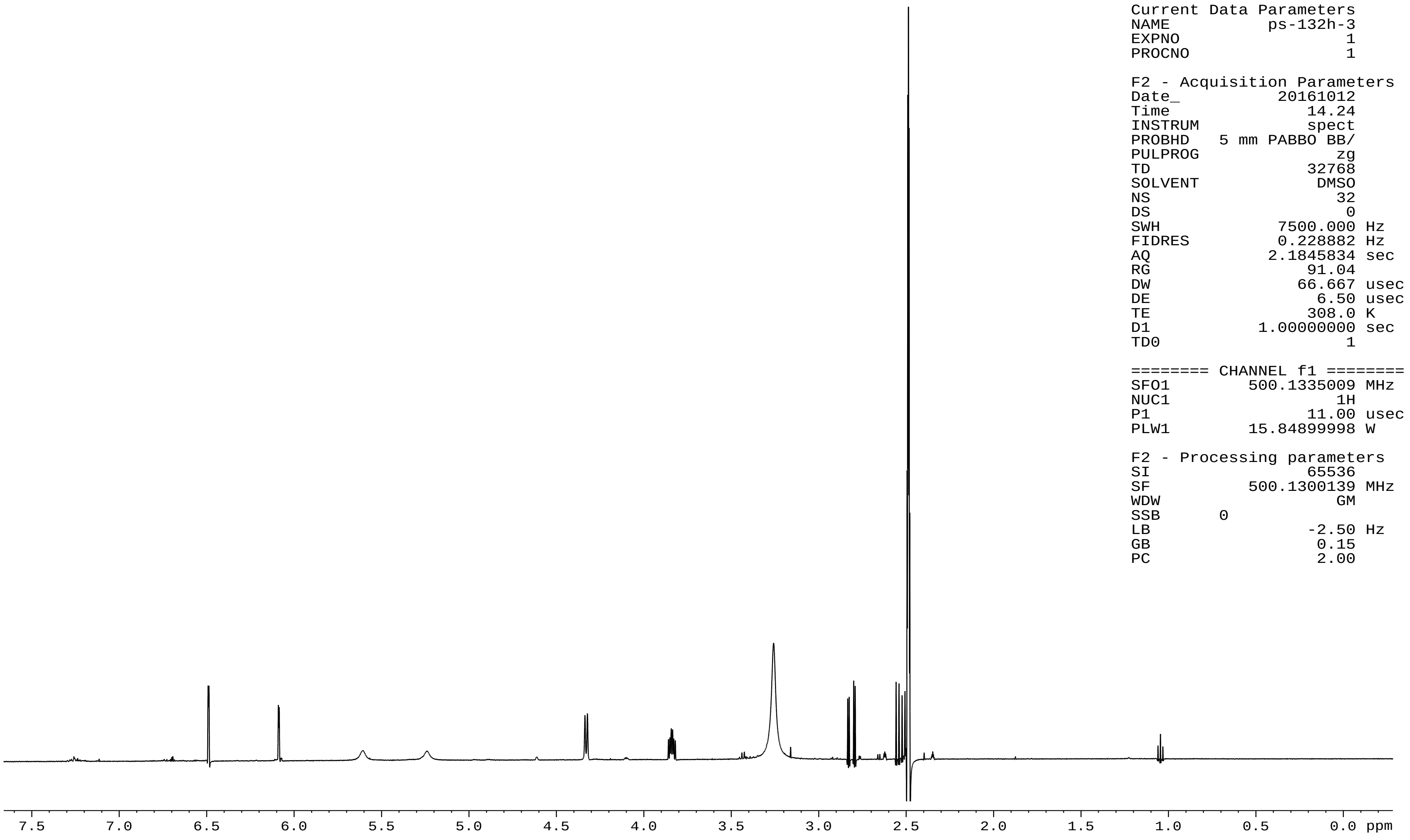

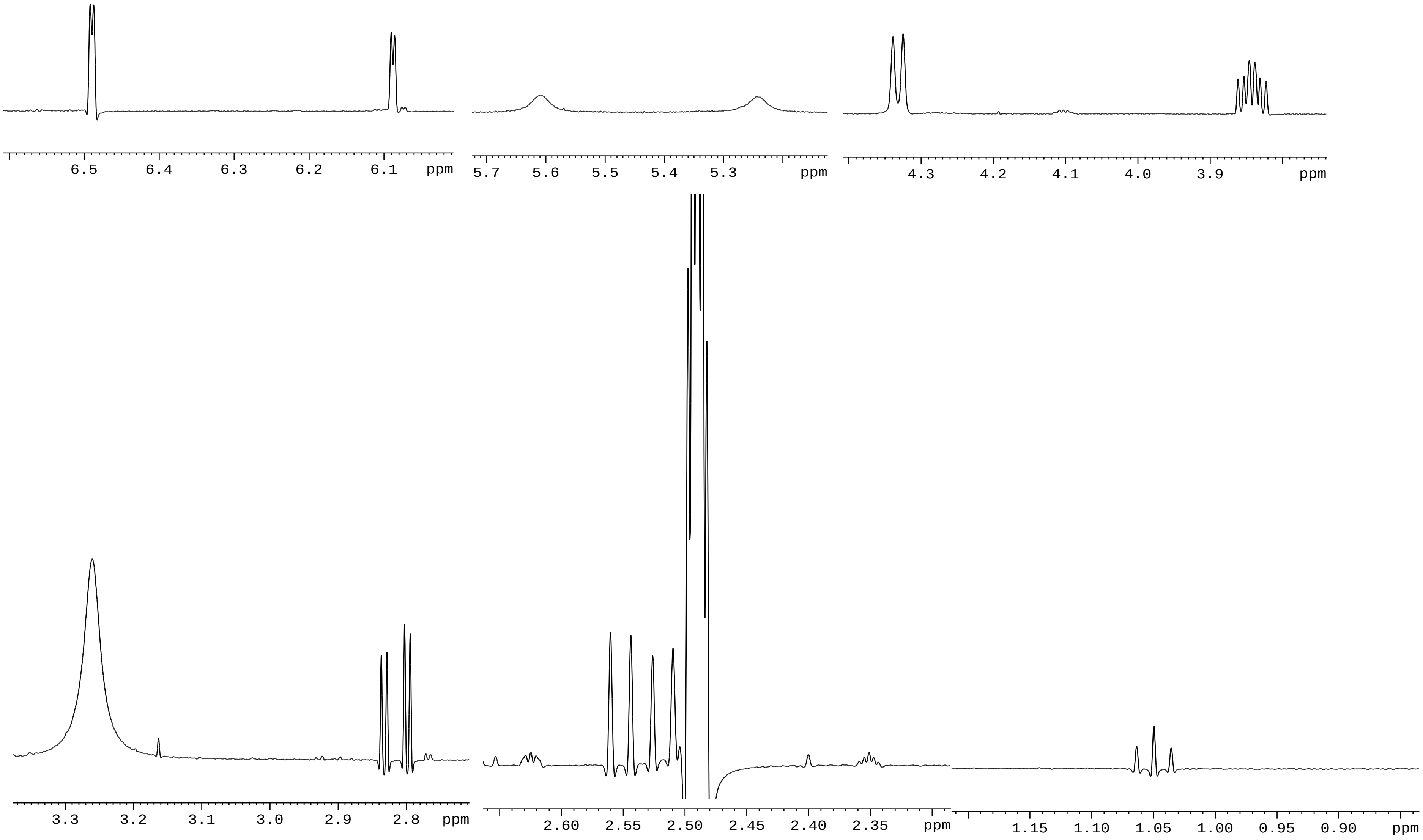
6.5 6.4 6.3 6.2 6.1 ppm
5.7 5.6 5.5 5.4 5.3 ppm
4.3 4.2 4.1 4.0 3.9 ppm
3.3 3.2 3.1 3.0 2.9 2.8 ppm
2.60 2.55 2.50 2.45 2.40 2.35 ppm
1.15 1.10 1.05 1.00 0.95 0.90 ppm

**Figure S2.** $^{13}$C **NMR (125 MHz, DMSO-*d*$_6$) spectrum of 4-hydroxyscytalone (1)**

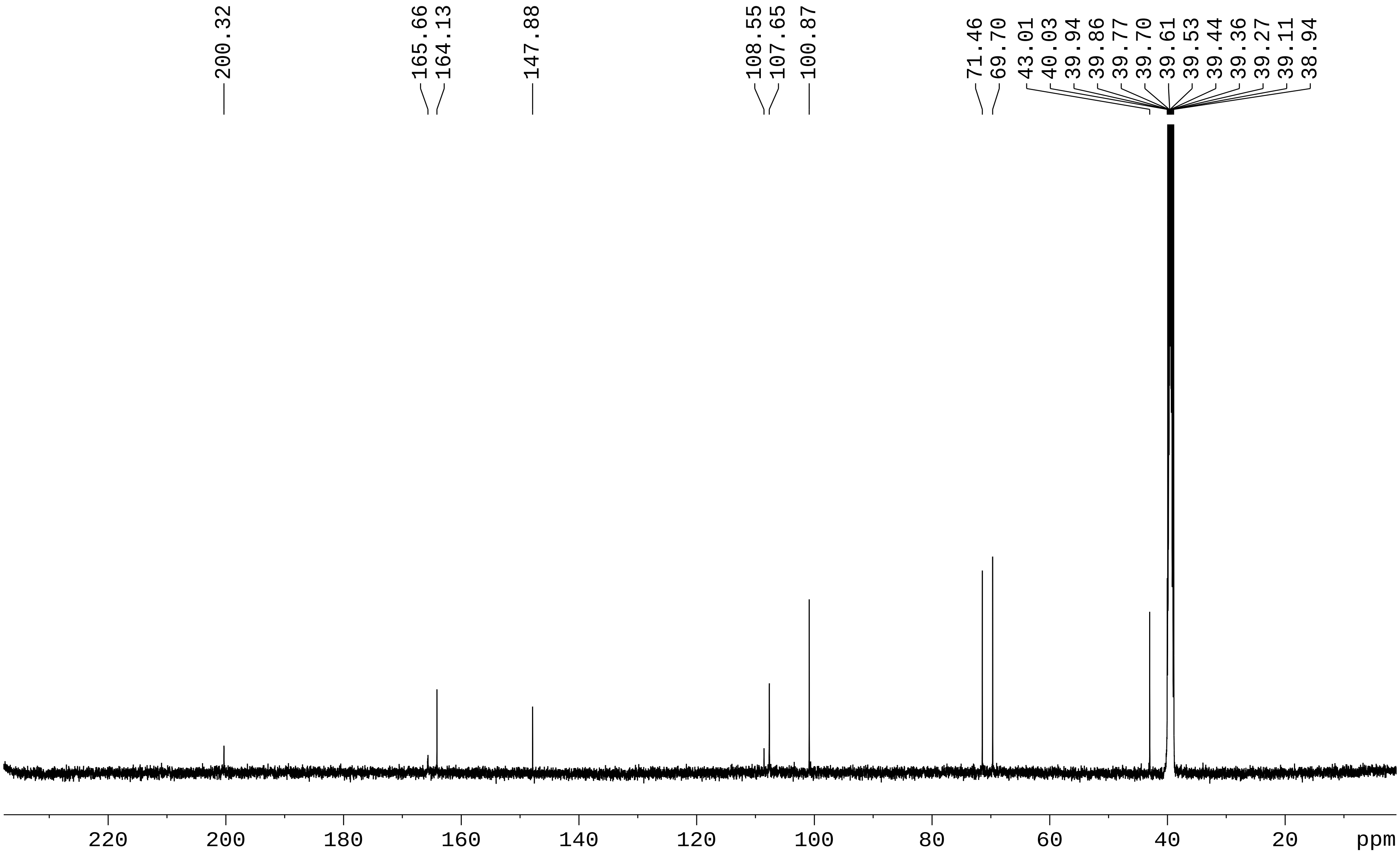

**Figure S3. CD spectrum of 4-hydroxyscytalone (1)**

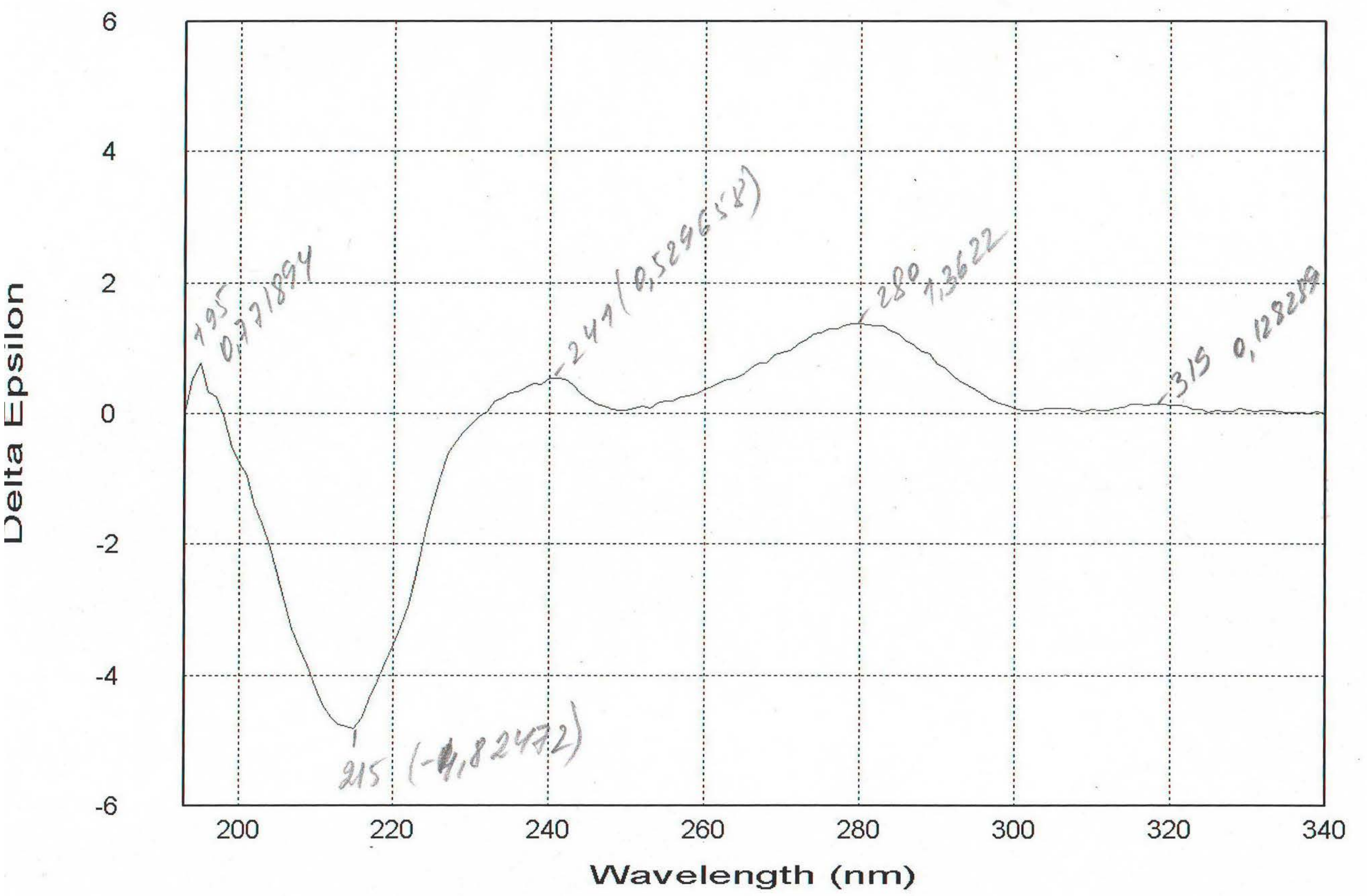

**Figure S4. $^{1}$H NMR (500 MHz, DMSO-$d_6$) spectrum of 4-hydroxy-6-dehydroxyscytalone (2)**

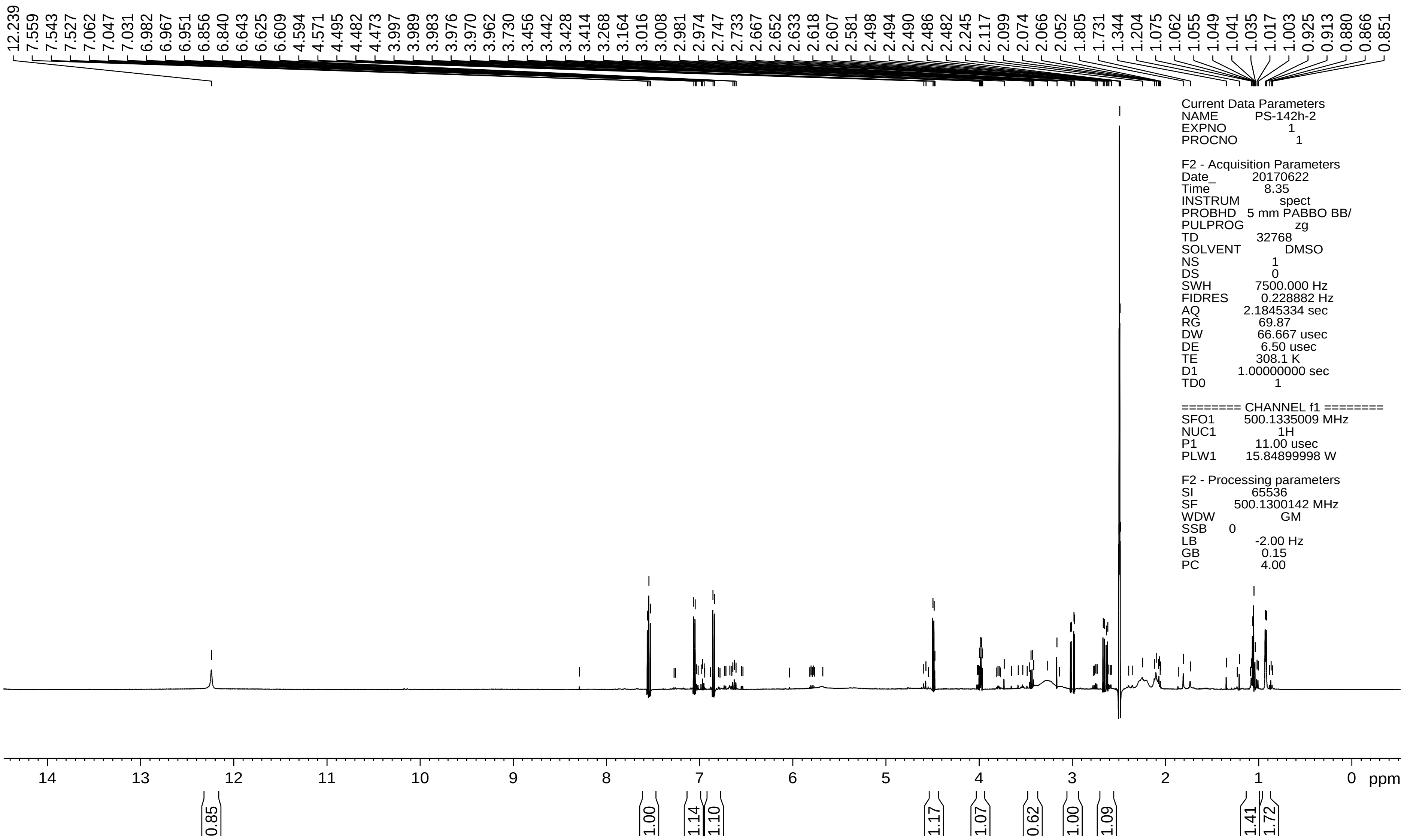

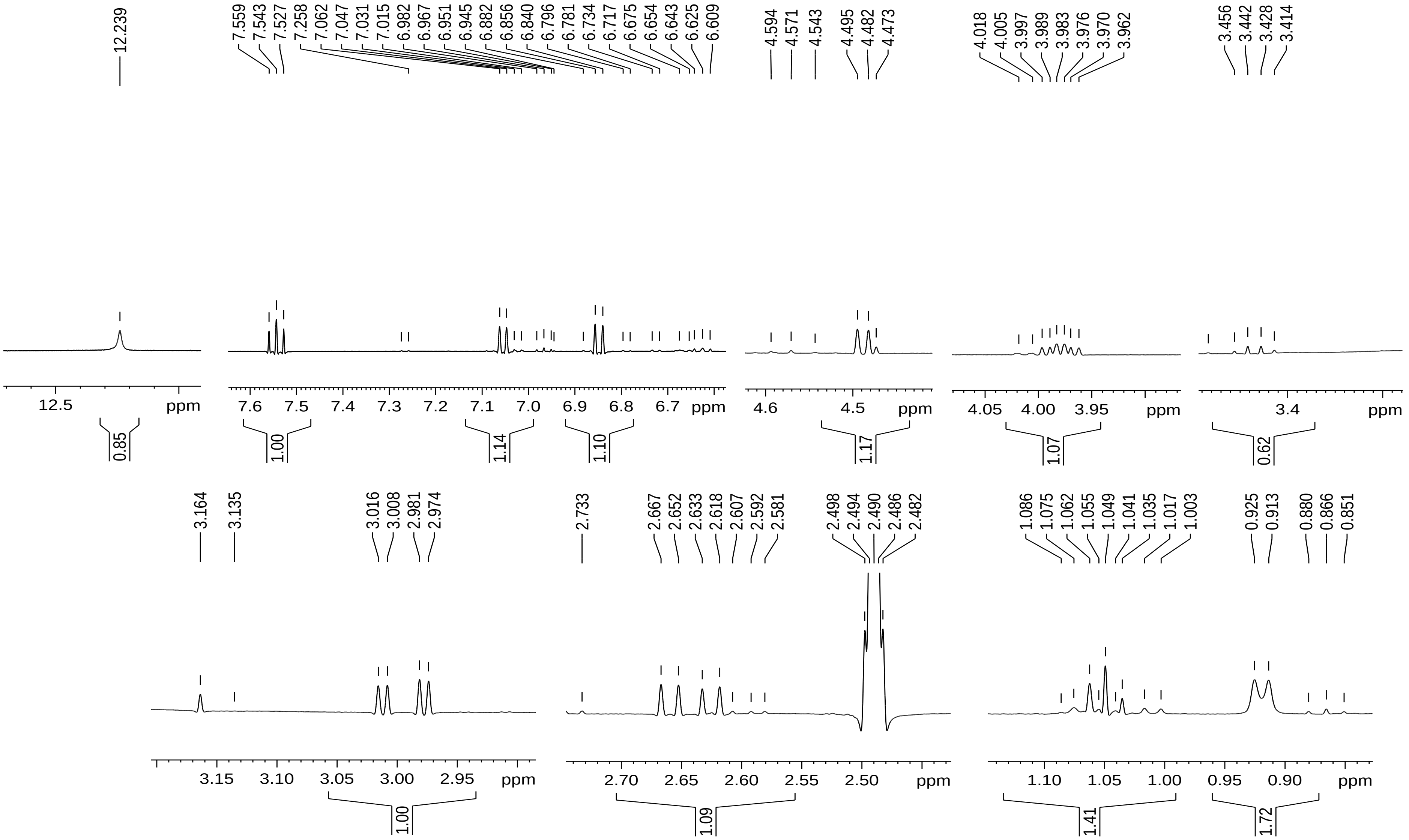
12.239
7.559 7.543 7.527 7.258 7.062 7.047 7.031 7.015 6.982 6.967 6.951 6.945 6.882 6.856 6.840 6.796 6.781 6.734 6.717 6.675 6.654 6.643 6.625 6.609
4.594 4.571 4.543 4.495 4.482 4.473
4.018 4.005 3.997 3.989 3.983 3.976 3.970 3.962
3.456 3.442 3.428 3.414
0.85 1.00 1.14 1.10 1.17 1.07 0.62
3.164 3.135 3.016 3.008 2.981 2.974
2.733 2.667 2.652 2.633 2.618 2.607 2.592 2.581
2.498 2.494 2.490 2.486 2.482
1.086 1.075 1.062 1.055 1.049 1.041 1.035 1.017 1.003
0.925 0.913 0.880 0.866 0.851
1.00 1.09 1.41 1.72
ppm

**Figure S5. $^{13}$C NMR (500 MHz, DMSO-$d_6$) spectrum of 4-hydroxy-6-dehydroxyscytalone (2)**

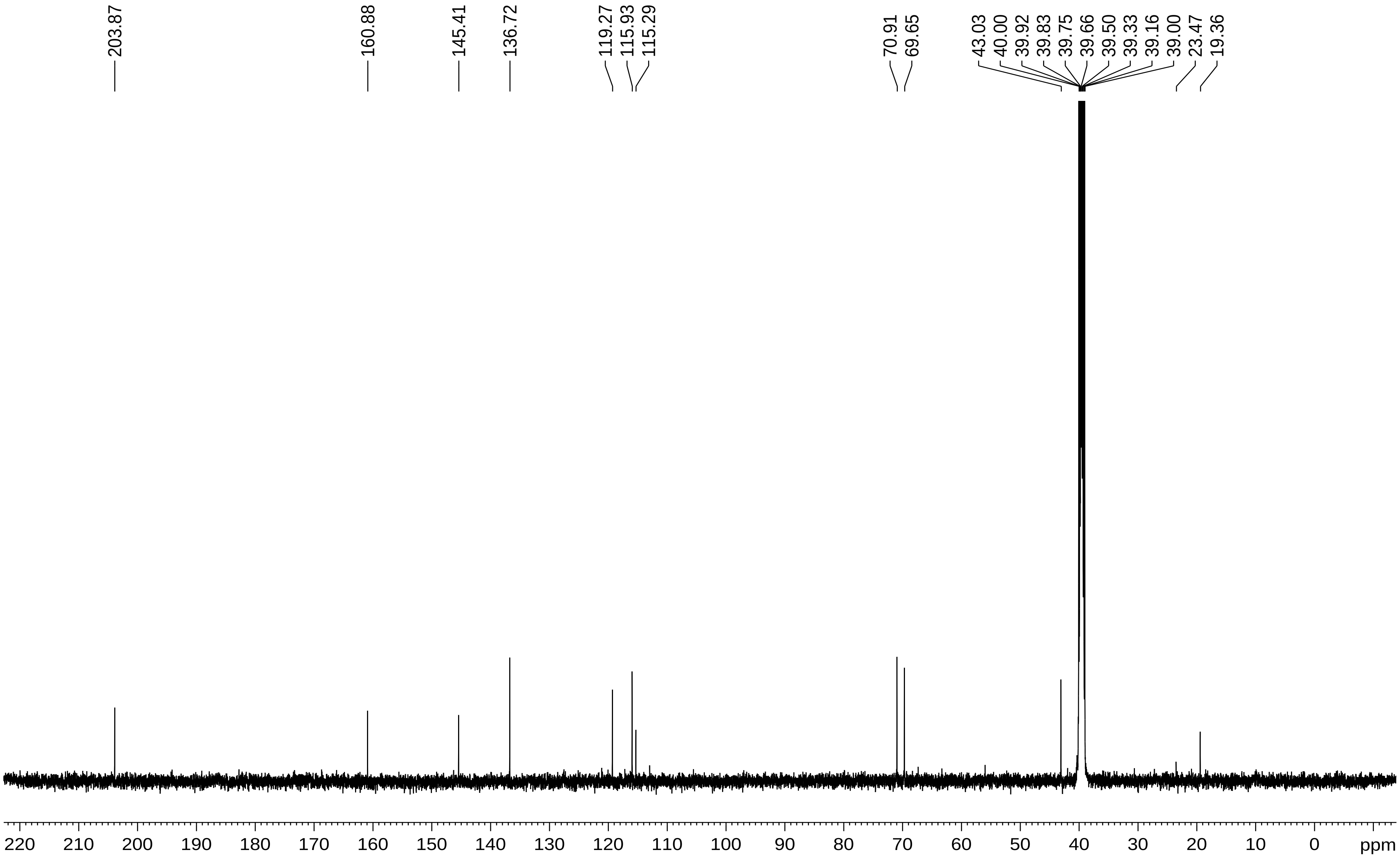

**Figure S6. DEPT NMR (125 MHz, DMSO-$d_6$) spectrum of 4-hydroxy-6-dehydroxyscytalone (2)**

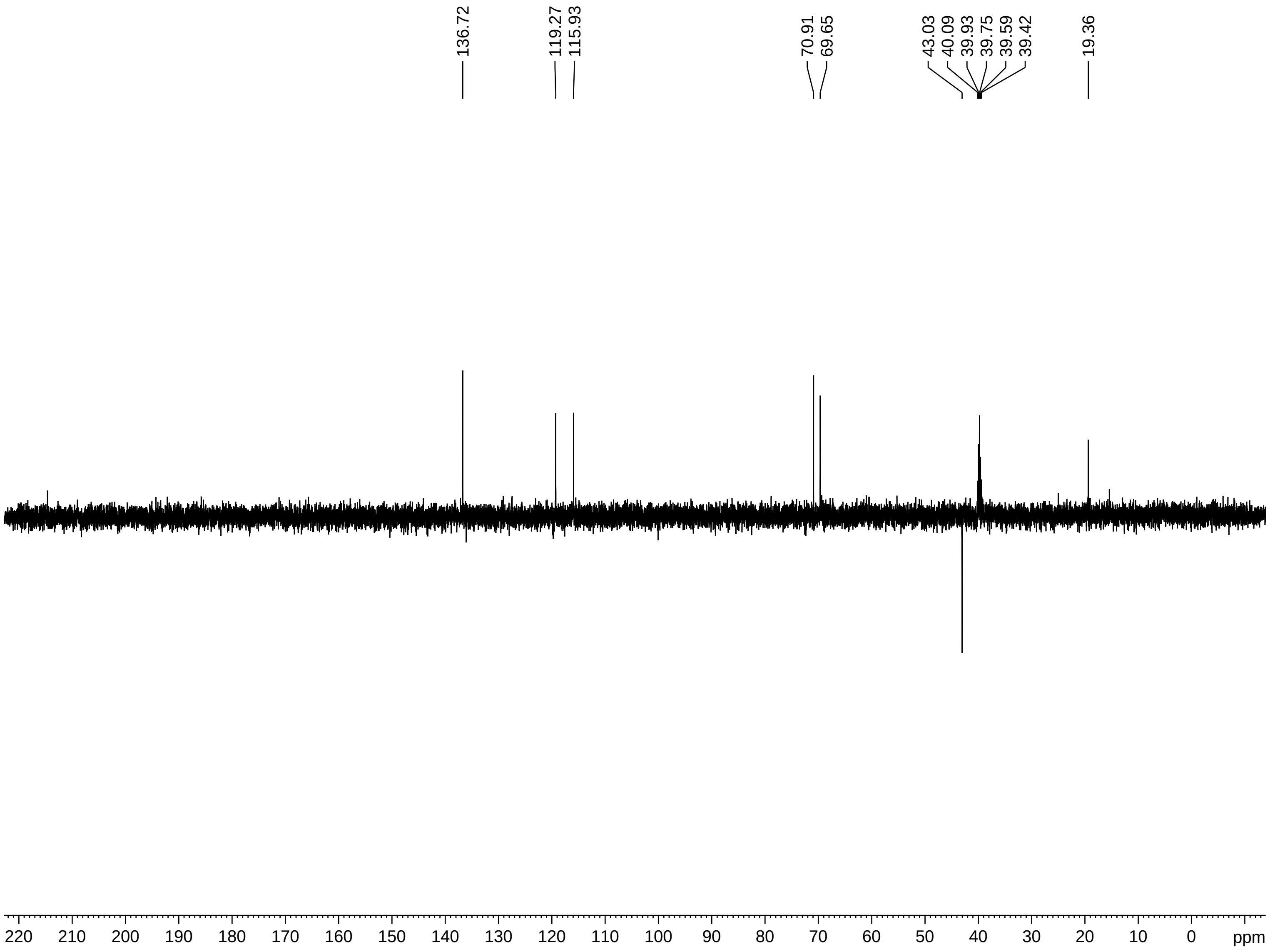


```
Current Data Parameters
NAME          PS-142h-2
EXPNO                75
PROCNO                1

F2 - Acquisition Parameters
Date_          20170622
Time              10.26
INSTRUM           spect
PROBHD   5 mm PABBO BB/
PULPROG         dept135
TD                65536
SOLVENT            DMSO
NS                   32
DS                   32
SWH          29761.904 Hz
FIDRES        0.454131 Hz
AQ           1.1010048 sec
RG               196.84
DW               16.800 usec
DE                 6.50 usec
TE                308.1 K
CNST2       145.0000000
D1           2.00000000 sec
D2           0.00344828 sec
D12          0.00002000 sec
TD0                4096

======== CHANNEL f1 ========
SFO1        125.7709936 MHz
NUC1                13C
P1                12.00 usec
P2                24.00 usec
PLW1        79.43299866 W

======== CHANNEL f2 ========
SFO2        500.1315004 MHz
NUC2                 1H
CPDPRG[2        waltz16
P3                11.00 usec
P4                22.00 usec
PCPD2             78.00 usec
PLW2        15.84899998 W
PLW12        0.31623000 W

F2 - Processing parameters
SI                32768
SF          125.7578626 MHz
WDW                  EM
SSB      0
LB                 1.00 Hz
GB       0
PC                 1.40
```

**Figure S7. CD spectrum of 4-hydroxy-6-dehydroxyscytalone (2)**

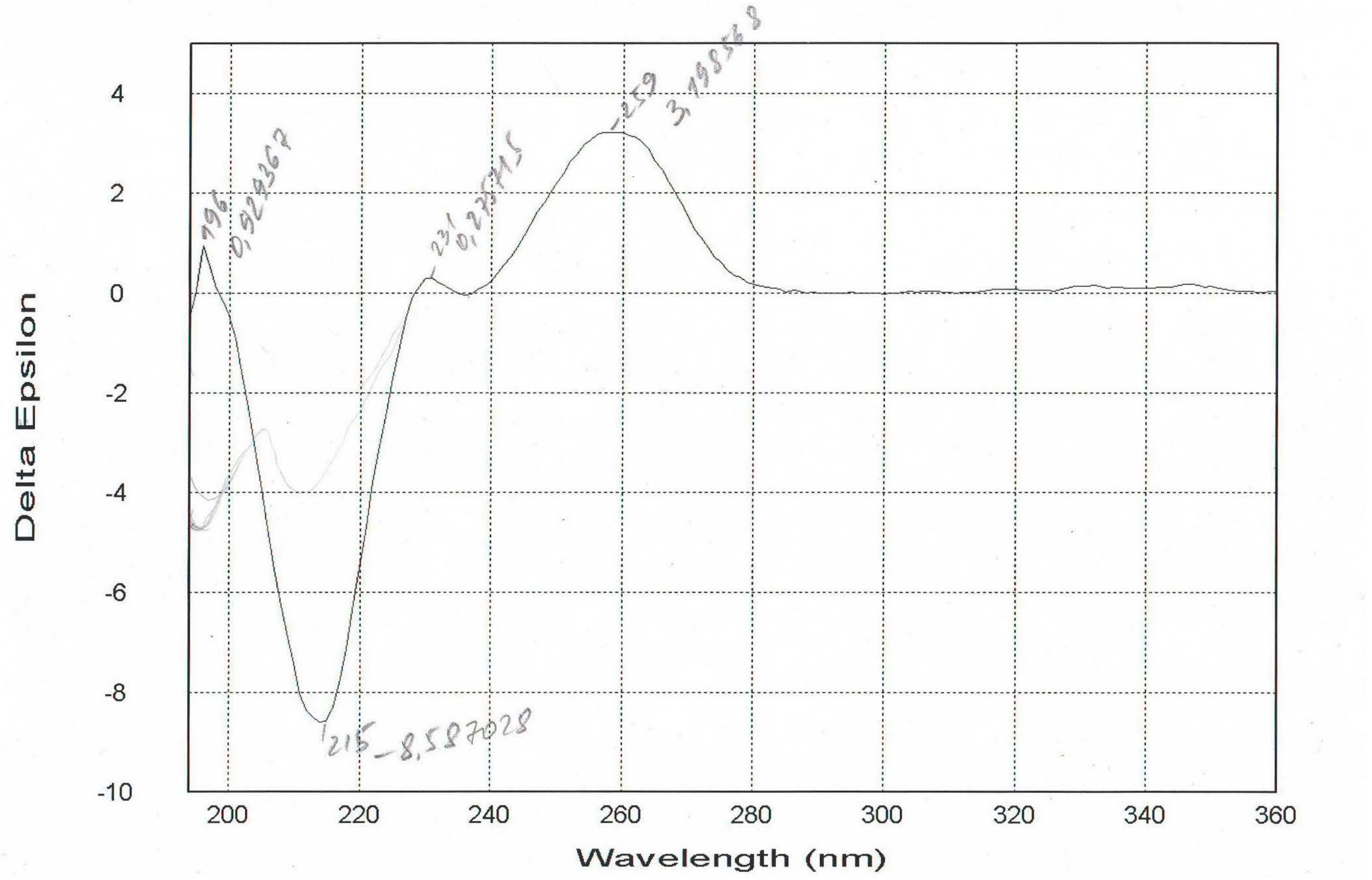

**Figure S8. $^1$H NMR (500 MHz, DMSO-$d_6$) spectrum of demethylcitreoviranol (3)**

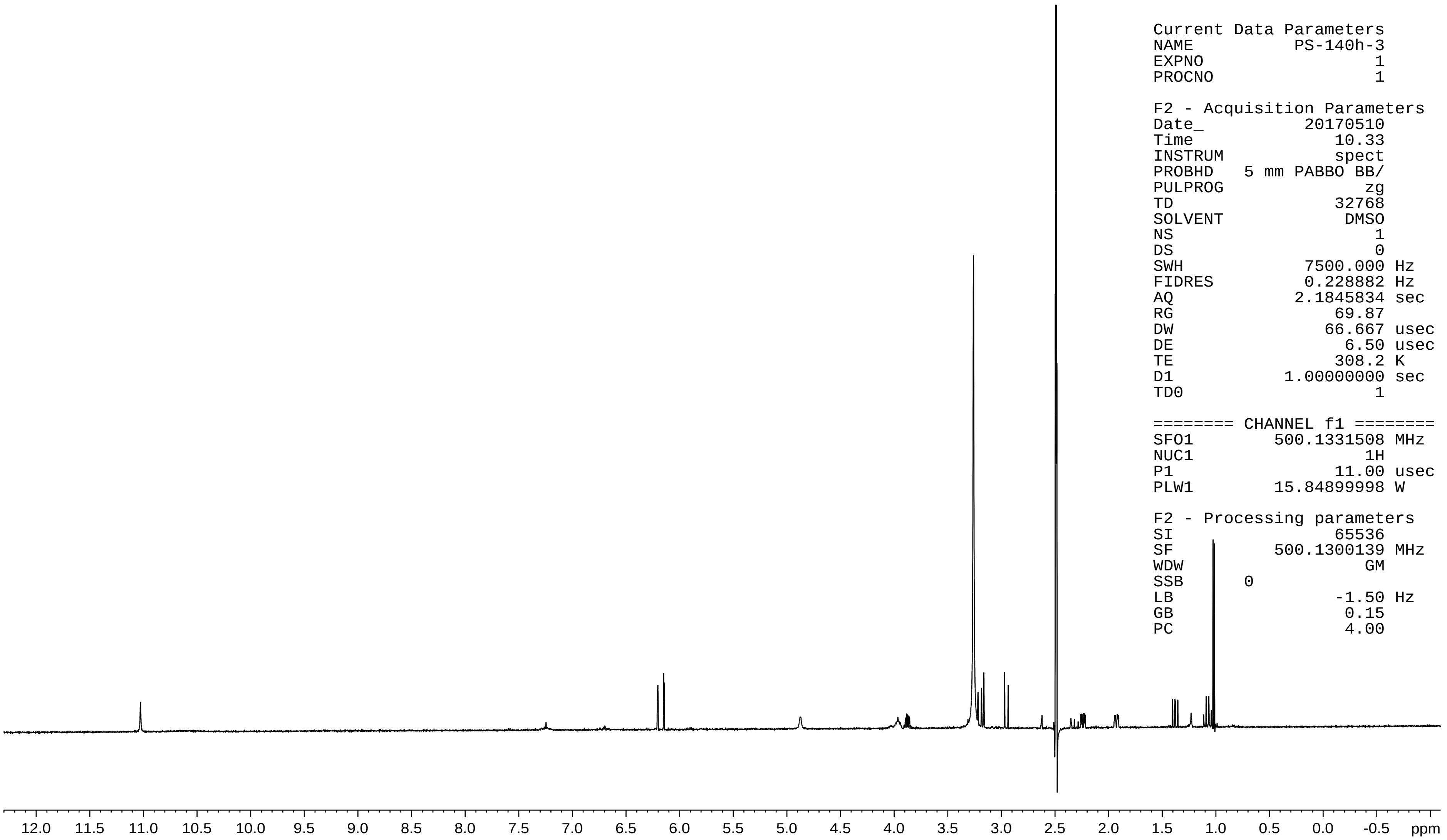

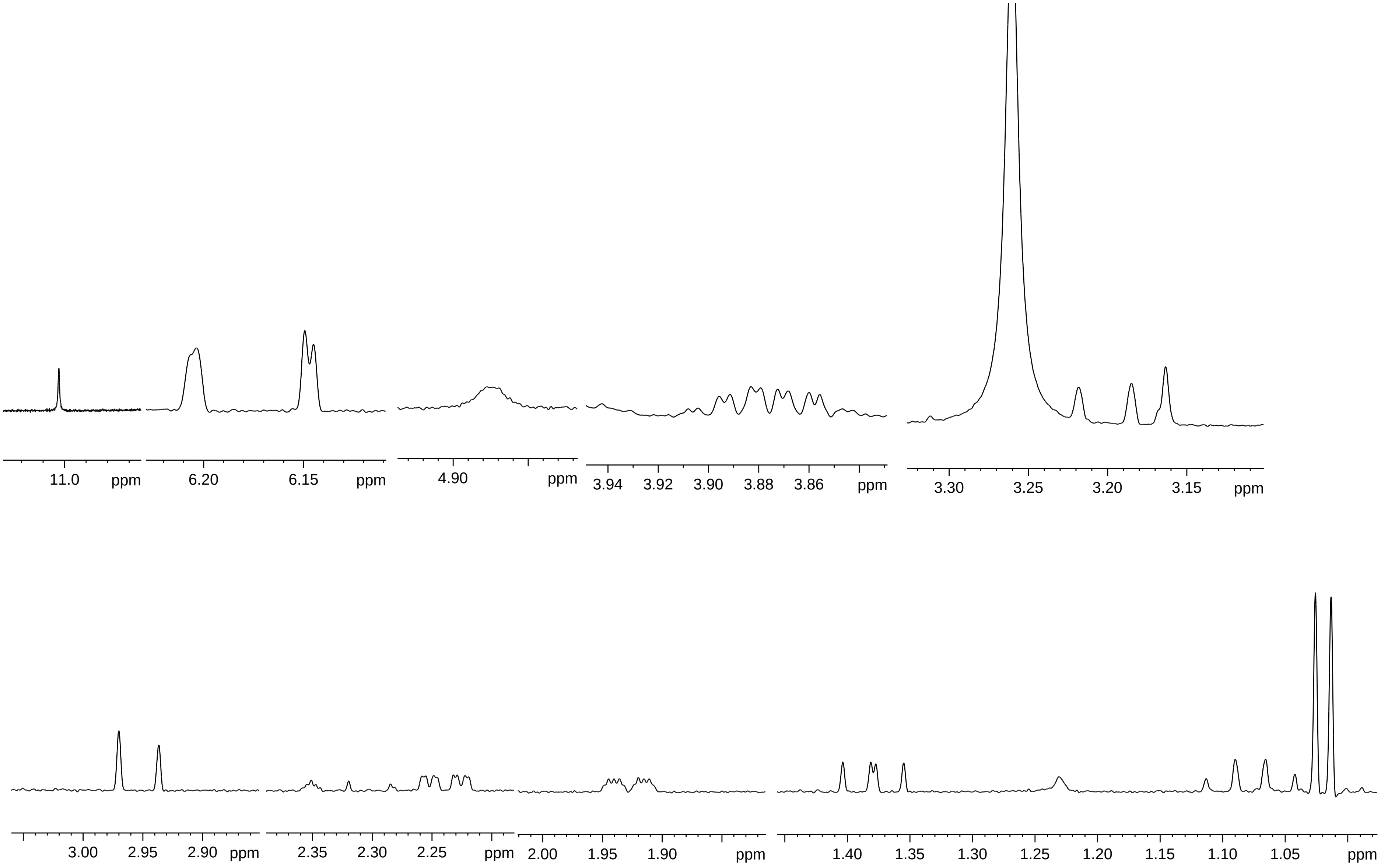


**Figure S9. $^{13}$C NMR (125 MHz, DMSO-$d_6$) spectrum of demethylcitreoviranol (3)**

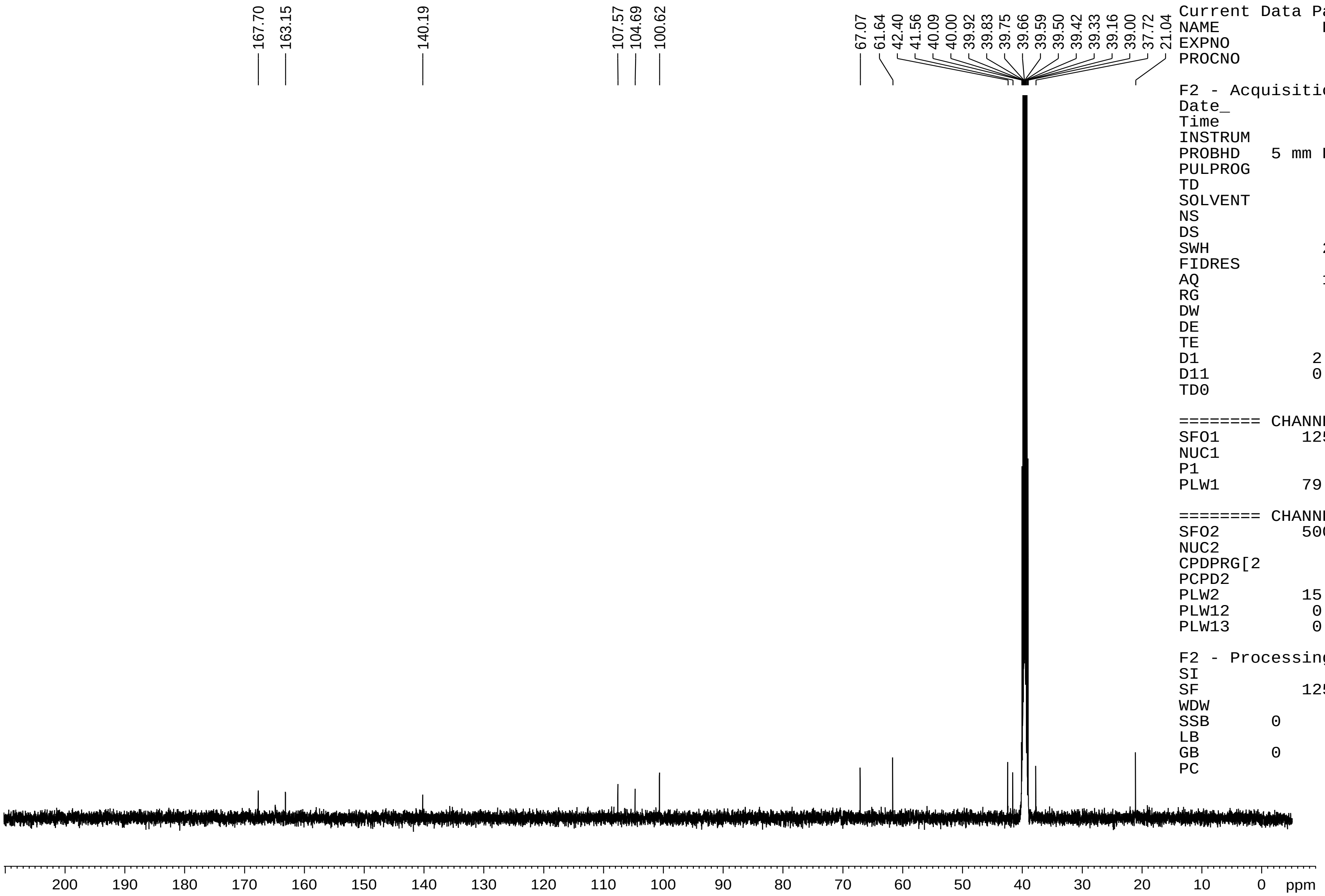


Current Data Parameters
NAME PS-140h-3
EXPNO 11
PROCNO 1

F2 - Acquisition Parameters
Date_ 20170510
Time 11.43
INSTRUM spect
PROBHD 5 mm PABBO BB/
PULPROG zgpg
TD 65536
SOLVENT DMSO
NS 2522
DS 0
SWH 27573.529 Hz
FIDRES 0.420739 Hz
AQ 1.1883861 sec
RG 196.84
DW 18.133 usec
DE 6.50 usec
TE 308.1 K
D1 2.00000000 sec
D11 0.03000000 sec
TD0 4096

======== CHANNEL f1 ========
SFO1 125.7709936 MHz
NUC1 13C
P1 12.00 usec
PLW1 79.43299866 W

======== CHANNEL f2 ========
SFO2 500.1320005 MHz
NUC2 1H
CPDPRG[2 waltz16
PCPD2 78.00 usec
PLW2 15.84899998 W
PLW12 0.31623000 W
PLW13 0.15849000 W

F2 - Processing parameters
SI 65536
SF 125.7578635 MHz
WDW EM
SSB 0
LB 1.00 Hz
GB 0
PC 1.20

**Figure S10. DEPT NMR (125 MHz, DMSO-$d_6$) spectrum of demethylcitreoviranol (3)**

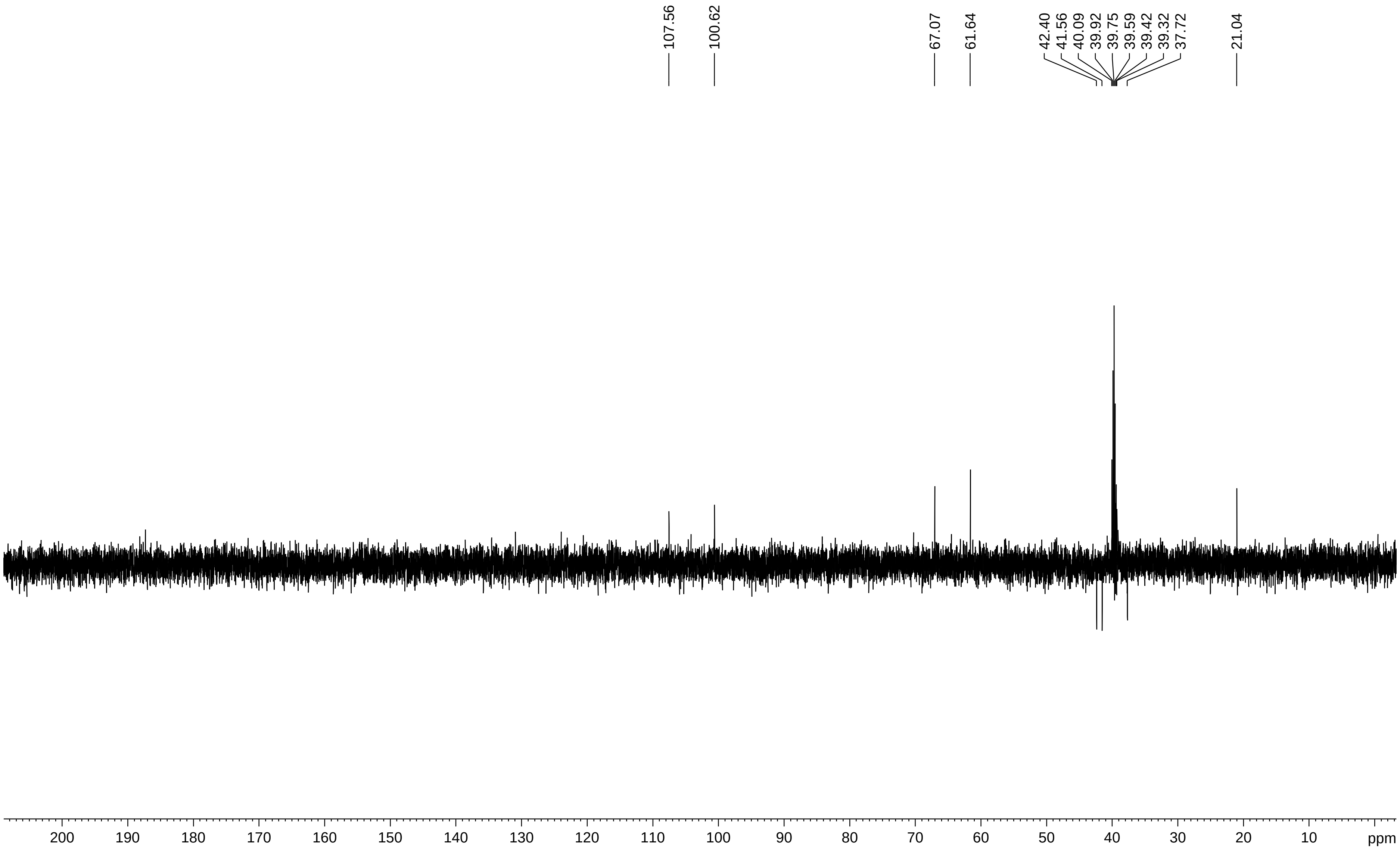

**Figure S11. CD spectrum of demethylcitreoviranol (3)**

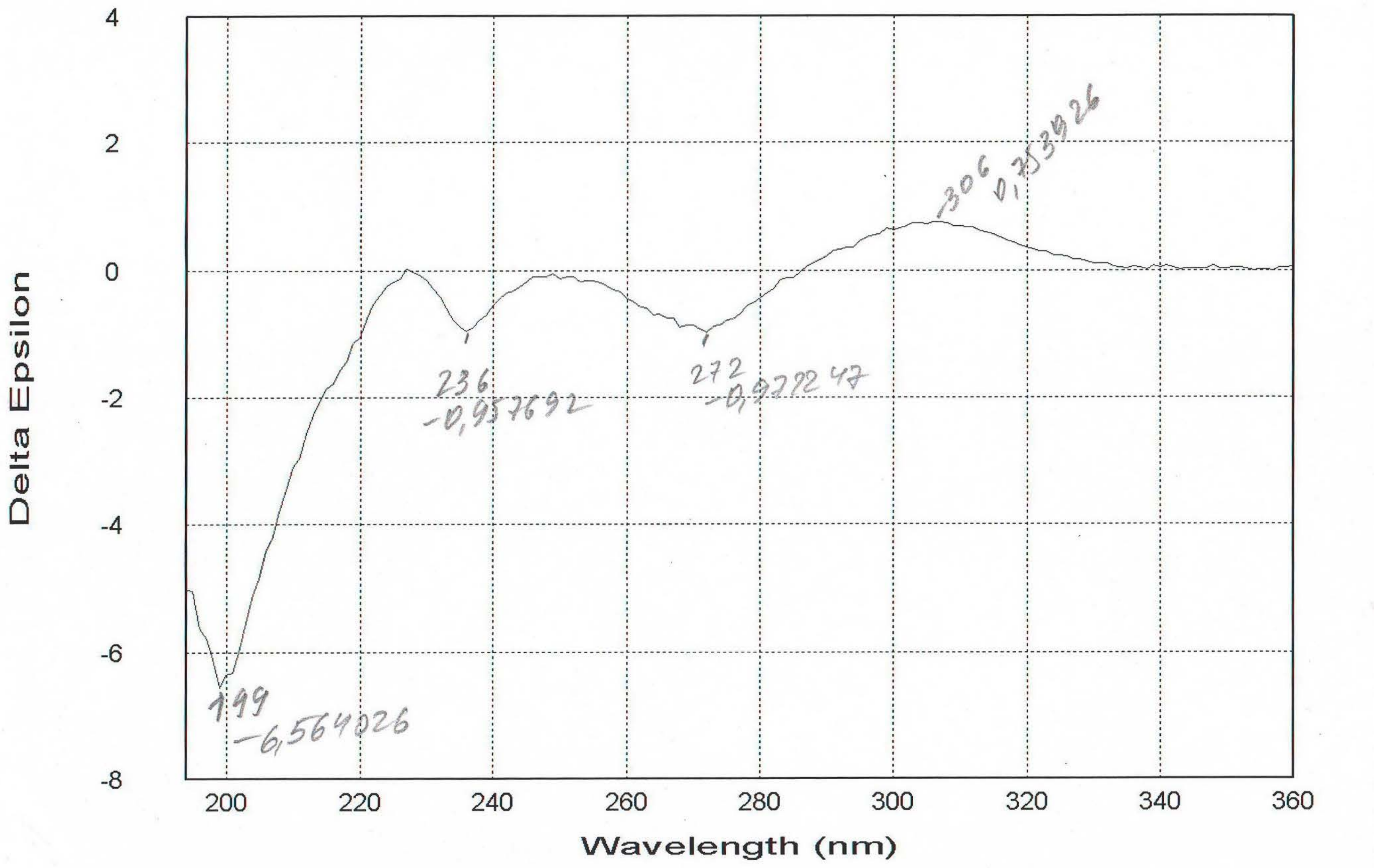

**Figure S12. $^{1}$H NMR (700 MHz, $CDCl_3$) spectrum of asterriquinone F (6)**

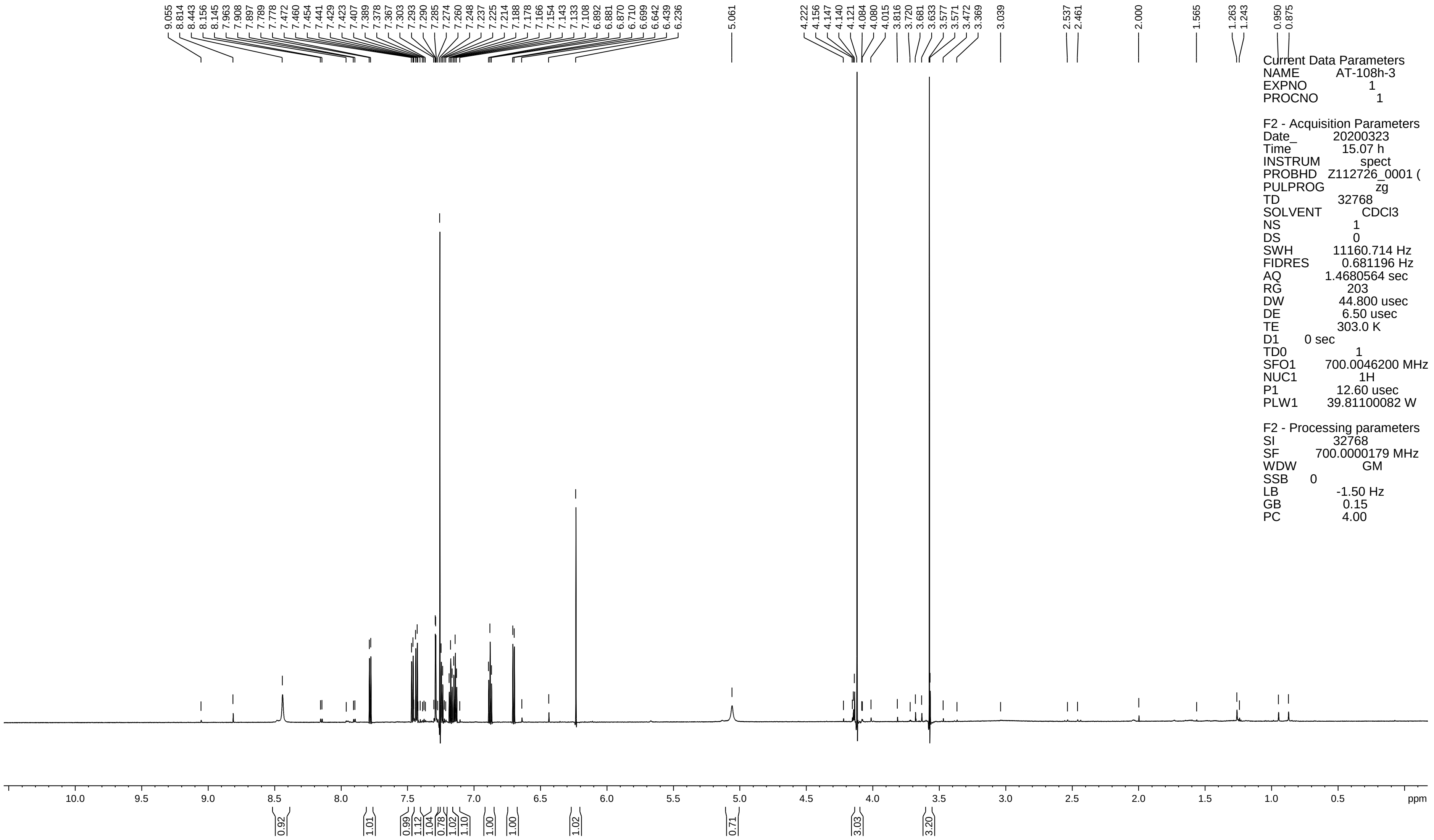

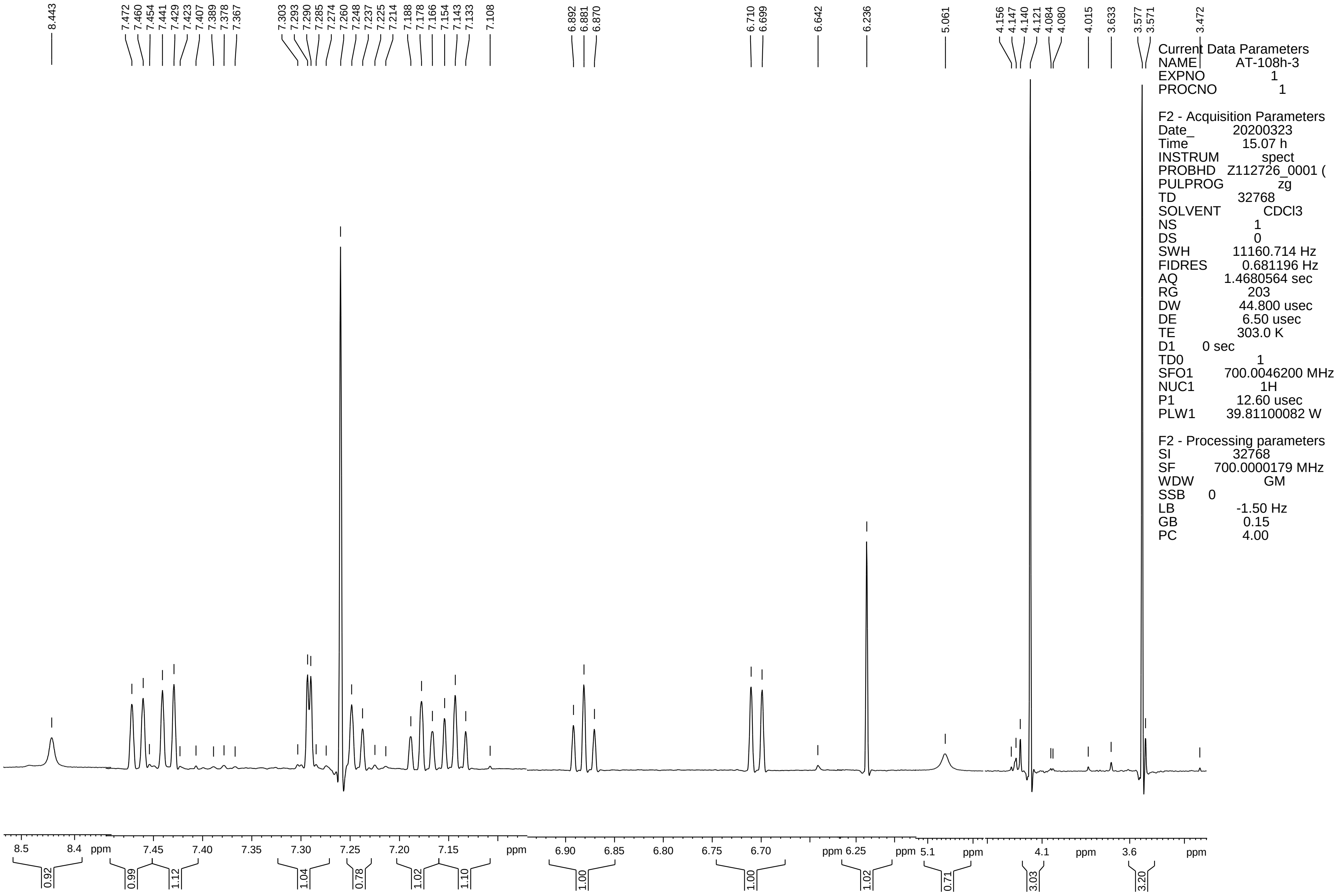
8.443
7.472 7.460 7.454 7.441 7.429 7.423 7.407 7.389 7.378 7.367
7.303 7.293 7.290 7.285 7.274 7.260 7.248 7.237 7.225 7.214 7.188 7.178 7.166 7.154 7.143 7.133 7.108
6.892 6.881 6.870
6.710 6.699
6.642
6.236
5.061
4.156 4.147 4.140 4.121 4.084 4.080 4.015
3.633 3.577 3.571 3.472
Current Data Parameters
NAME AT-108h-3
EXPNO 1
PROCNO 1
F2 - Acquisition Parameters
Date_ 20200323
Time 15.07 h
INSTRUM spect
PROBHD Z112726_0001 (
PULPROG zg
TD 32768
SOLVENT CDCl3
NS 1
DS 0
SWH 11160.714 Hz
FIDRES 0.681196 Hz
AQ 1.4680564 sec
RG 203
DW 44.800 usec
DE 6.50 usec
TE 303.0 K
D1 0 sec
TD0 1
SFO1 700.0046200 MHz
NUC1 1H
P1 12.60 usec
PLW1 39.81100082 W
F2 - Processing parameters
SI 32768
SF 700.0000179 MHz
WDW GM
SSB 0
LB -1.50 Hz
GB 0.15
PC 4.00
8.5 8.4 ppm
7.45 7.40 7.35 7.30 7.25 7.20 7.15 ppm
6.90 6.85 6.80 6.75 6.70 ppm
6.25 ppm
5.1 ppm
4.1 ppm
3.6 ppm
0.92
0.99 1.12
1.04 0.78 1.02 1.10
1.00
1.00
1.02
0.71
3.03
3.20

**Figure S13. $^{13}$C NMR (176 MHz, $CDCl_3$) spectrum of asterriquinone F (6)**

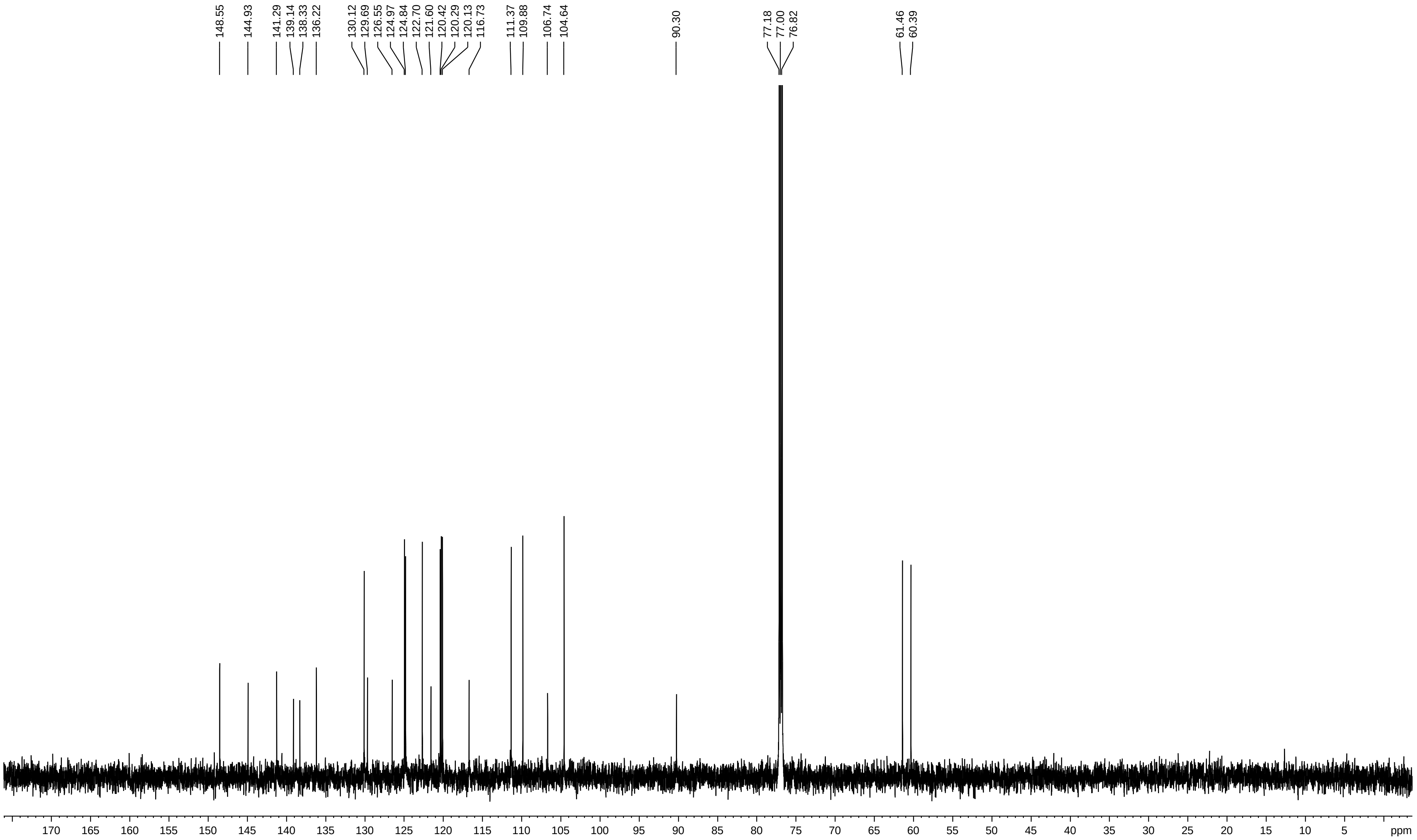

**Figure S14. DEPT (176 MHz, $CDCl_3$) spectrum of asterriquinone F (6)**

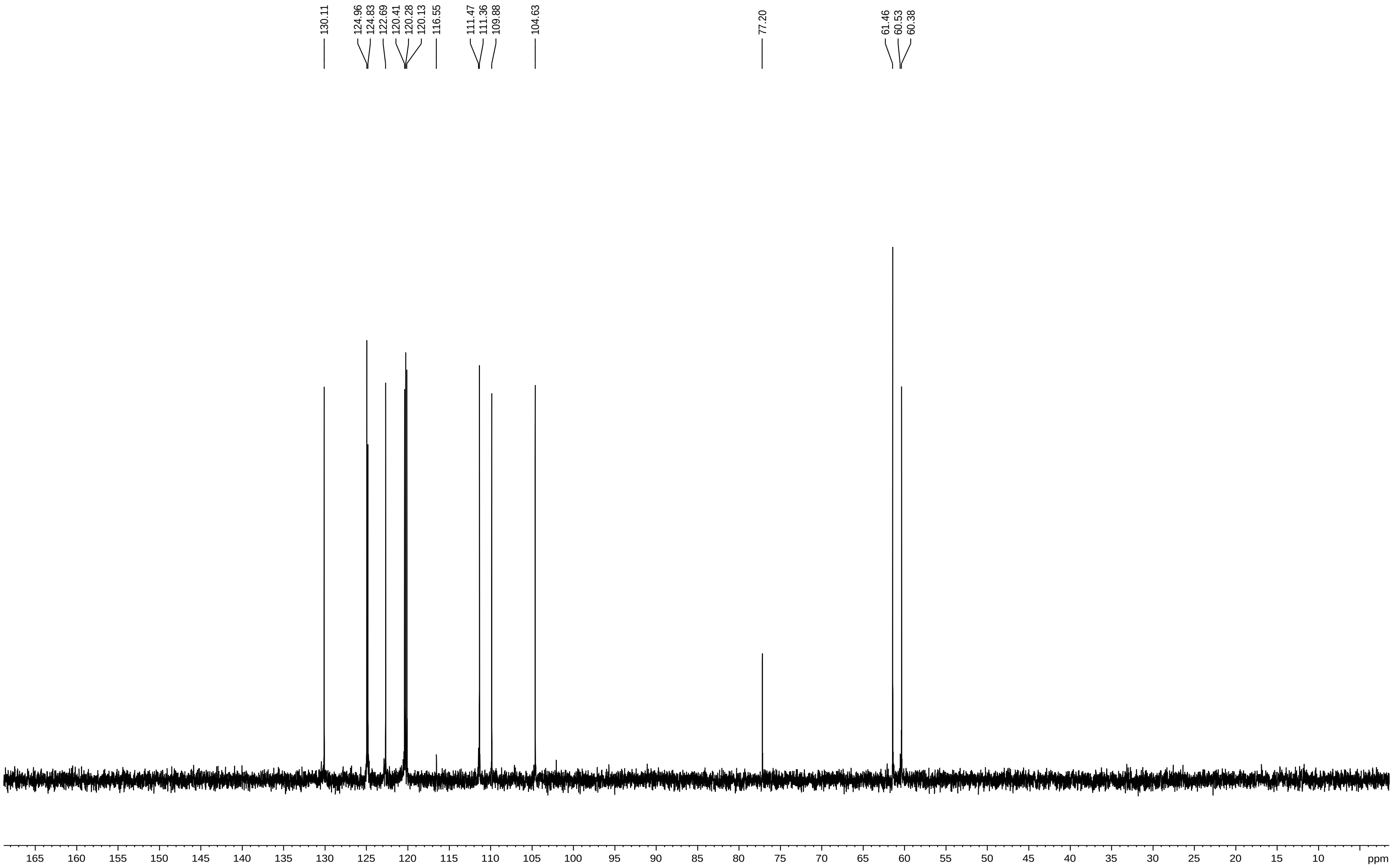

**Figure S15. HSQC (176 MHz, $CDCl_3$) spectrum of asterriquinone F (6)**

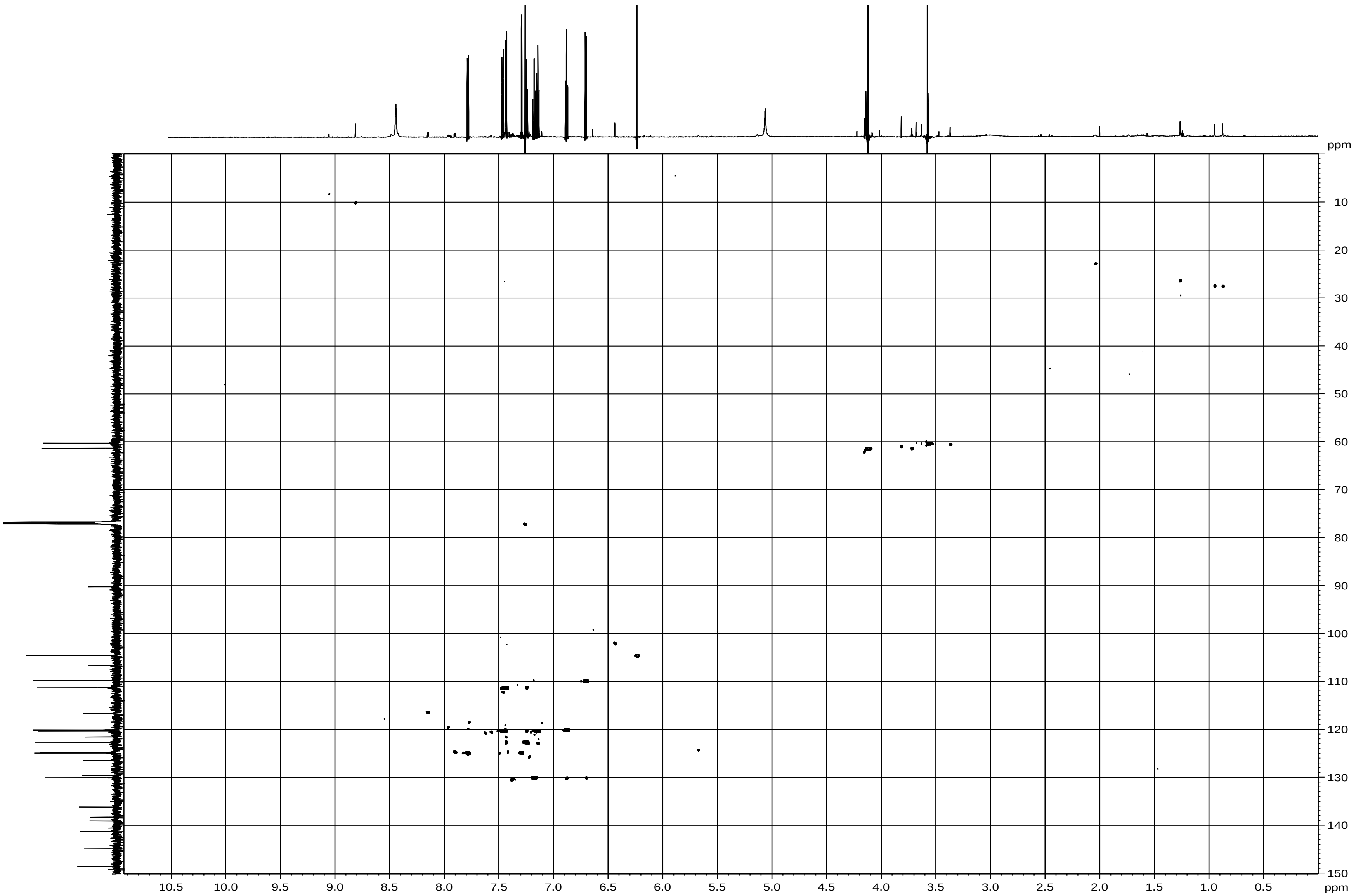

**Figure S16. HMBC (176 MHz, $CDCl_3$) spectrum of Asterriquinone F (6)**

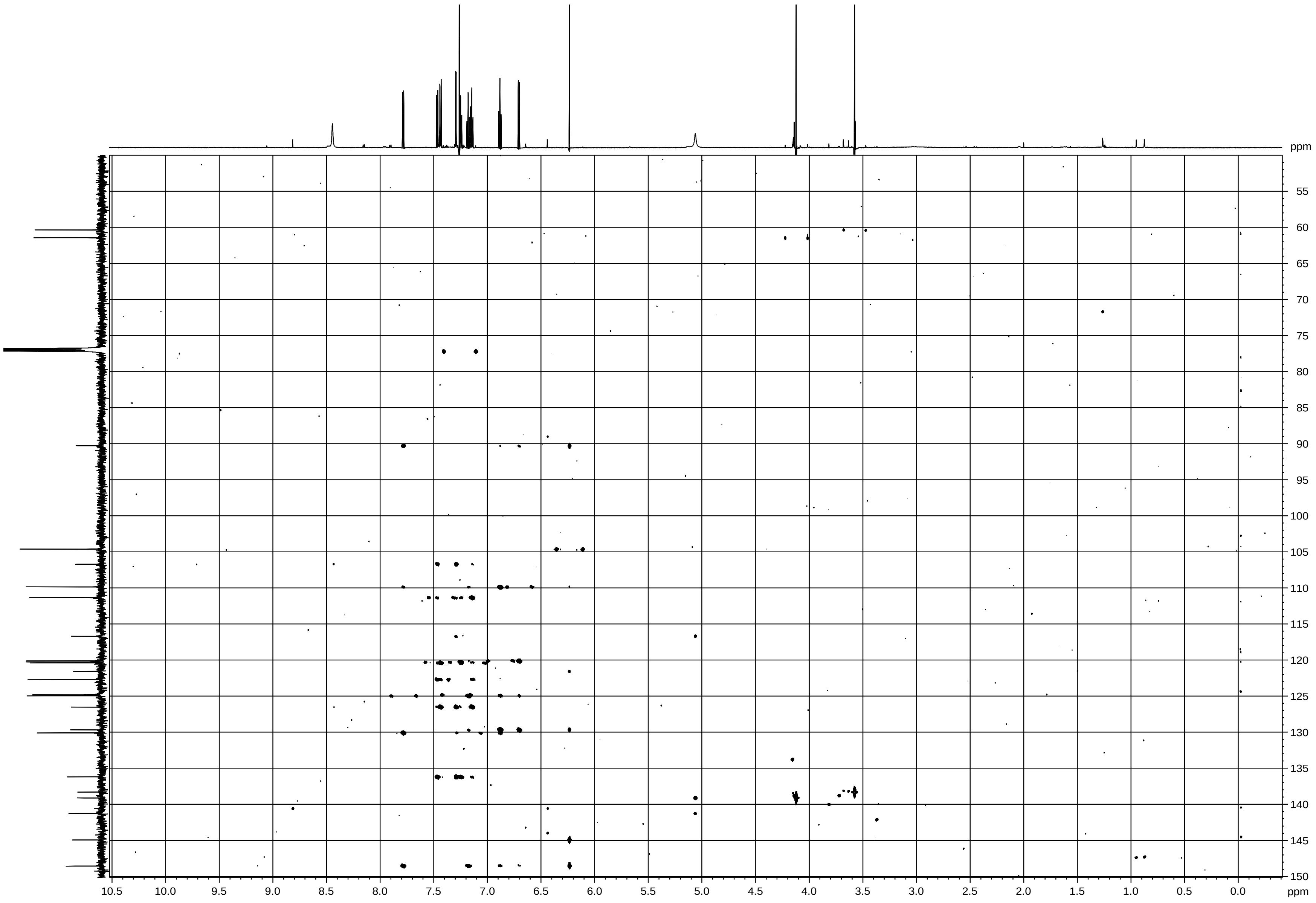

**Figure S17. COSY (176 MHz, CDCl$_3$) spectrum of asterriquinone F (6)**

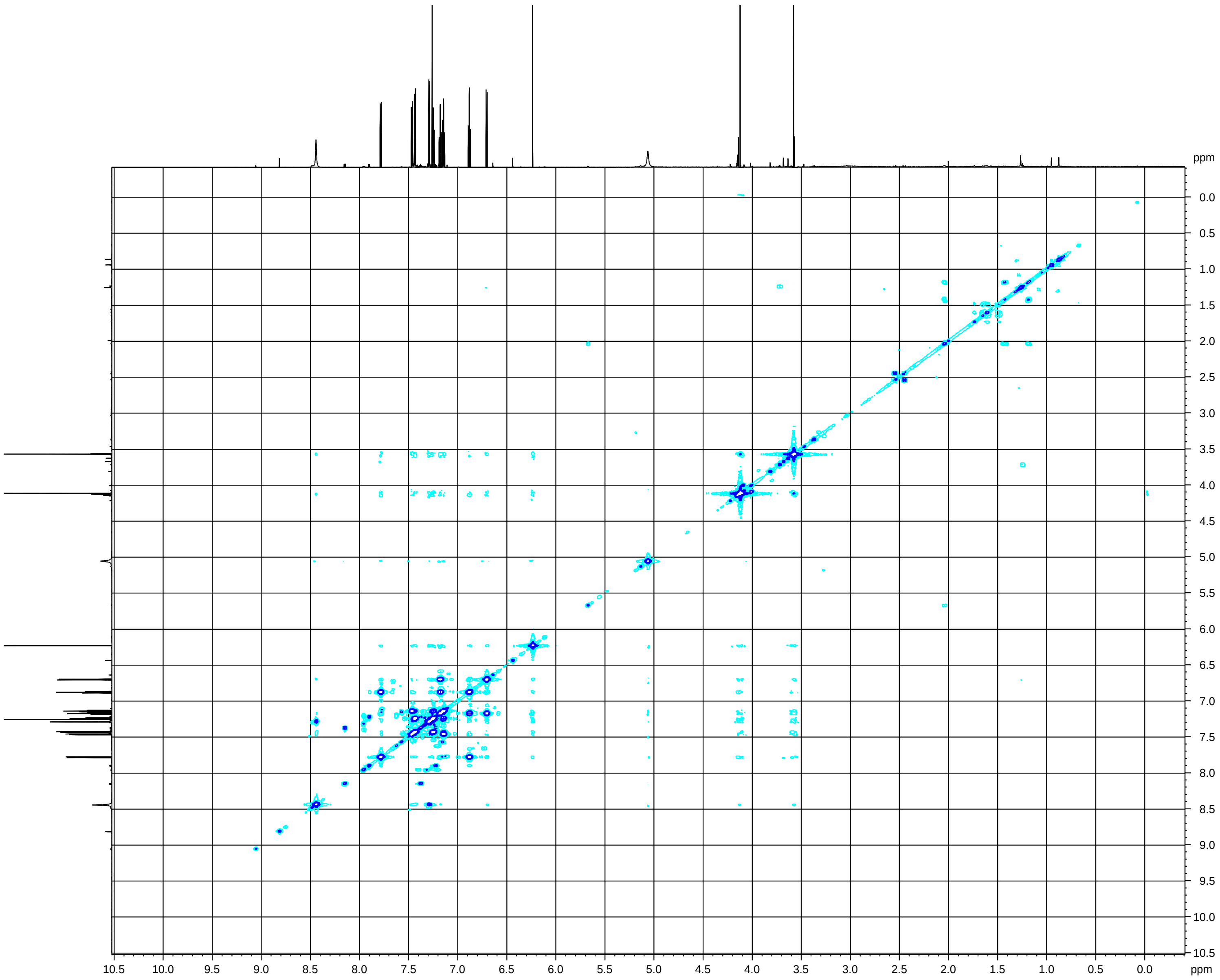

## Figure S18. ROESY (176 MHz, $CDCl_3$) spectrum of asterriquinone F (6)

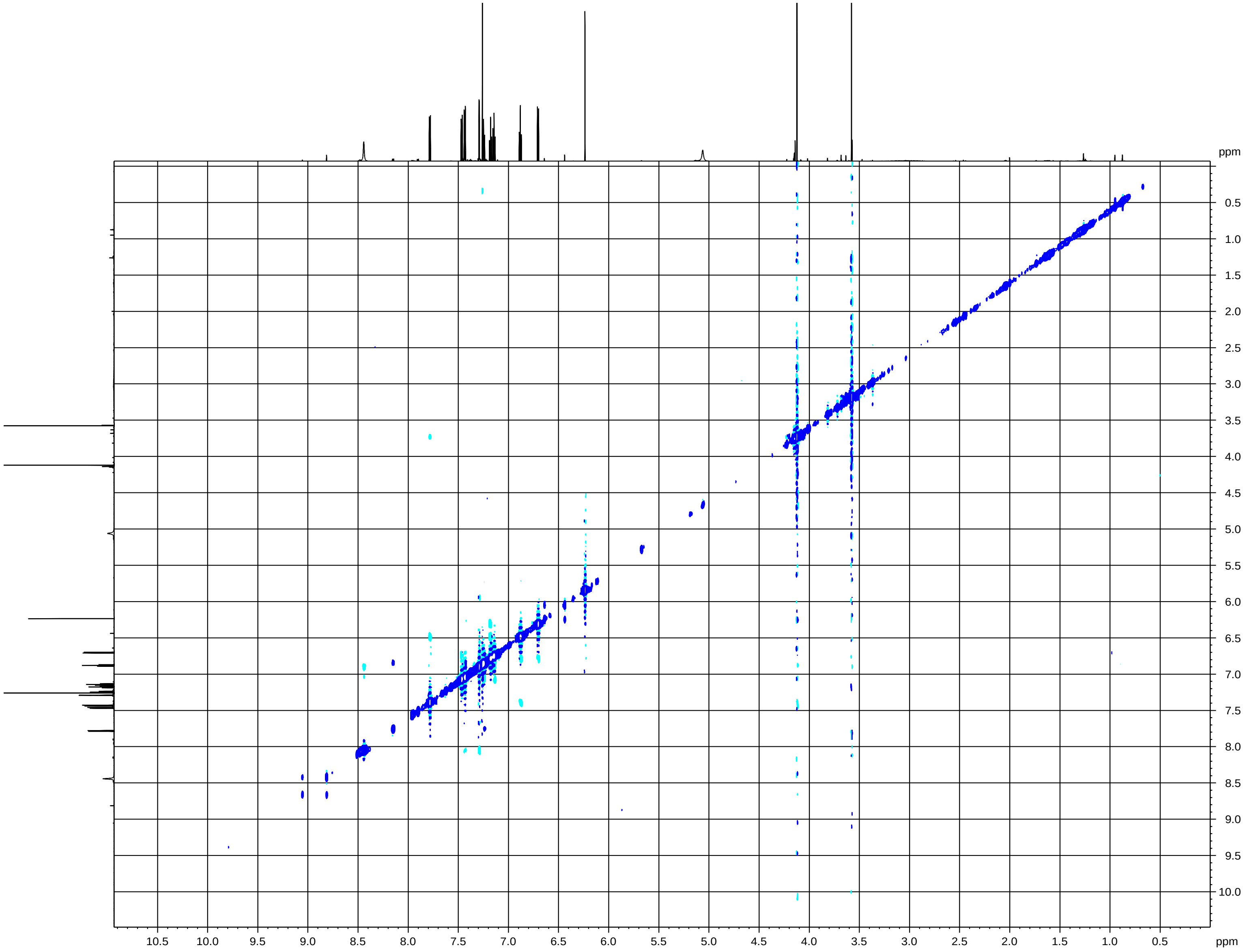

**Figure S19. CD spectrum of asterriquinone F (6)**

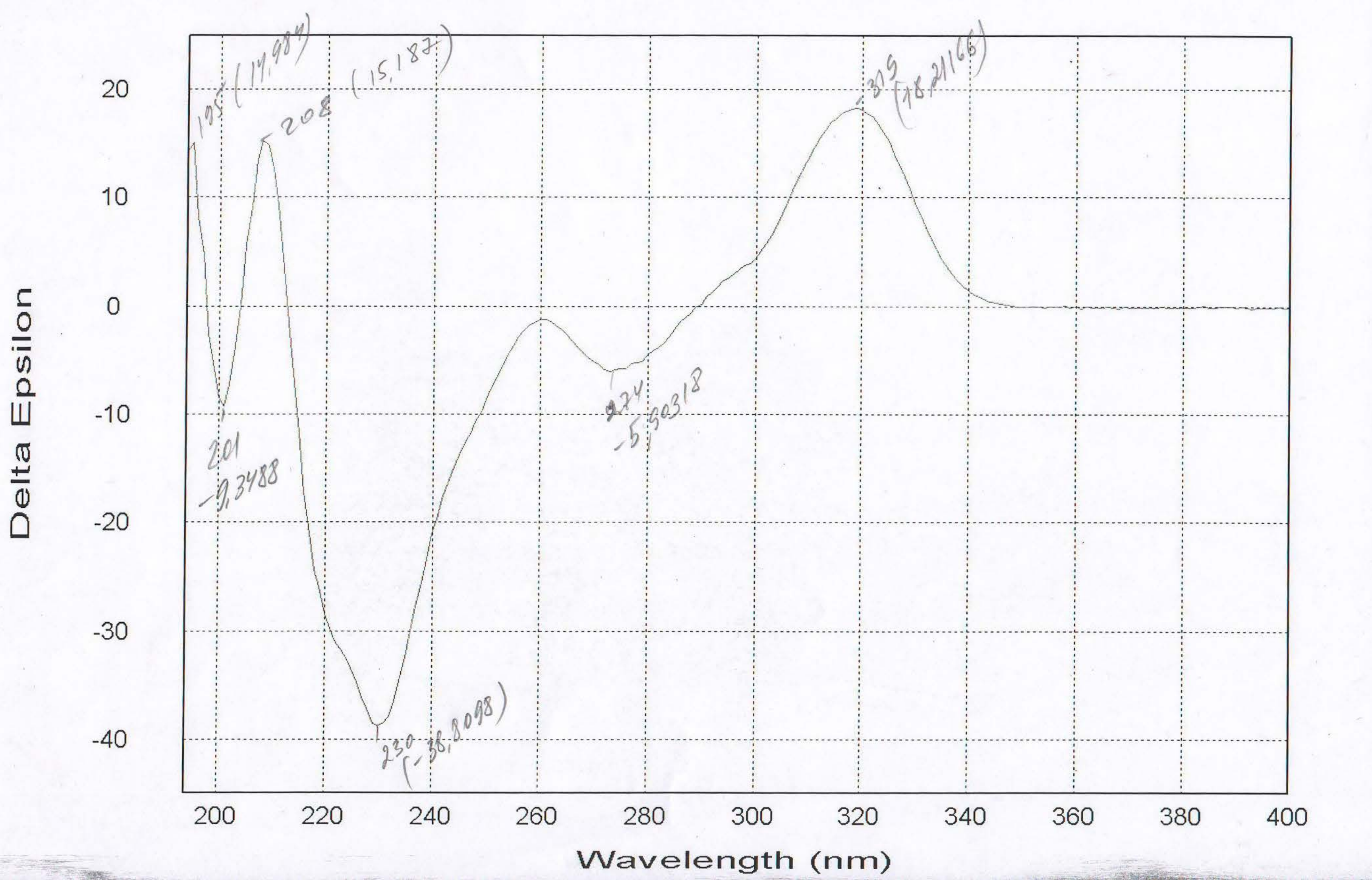

# Figure S20. $^{1}$H NMR (700 MHz, $CDCl_3$) spectrum of asterriquinone A3 (7)

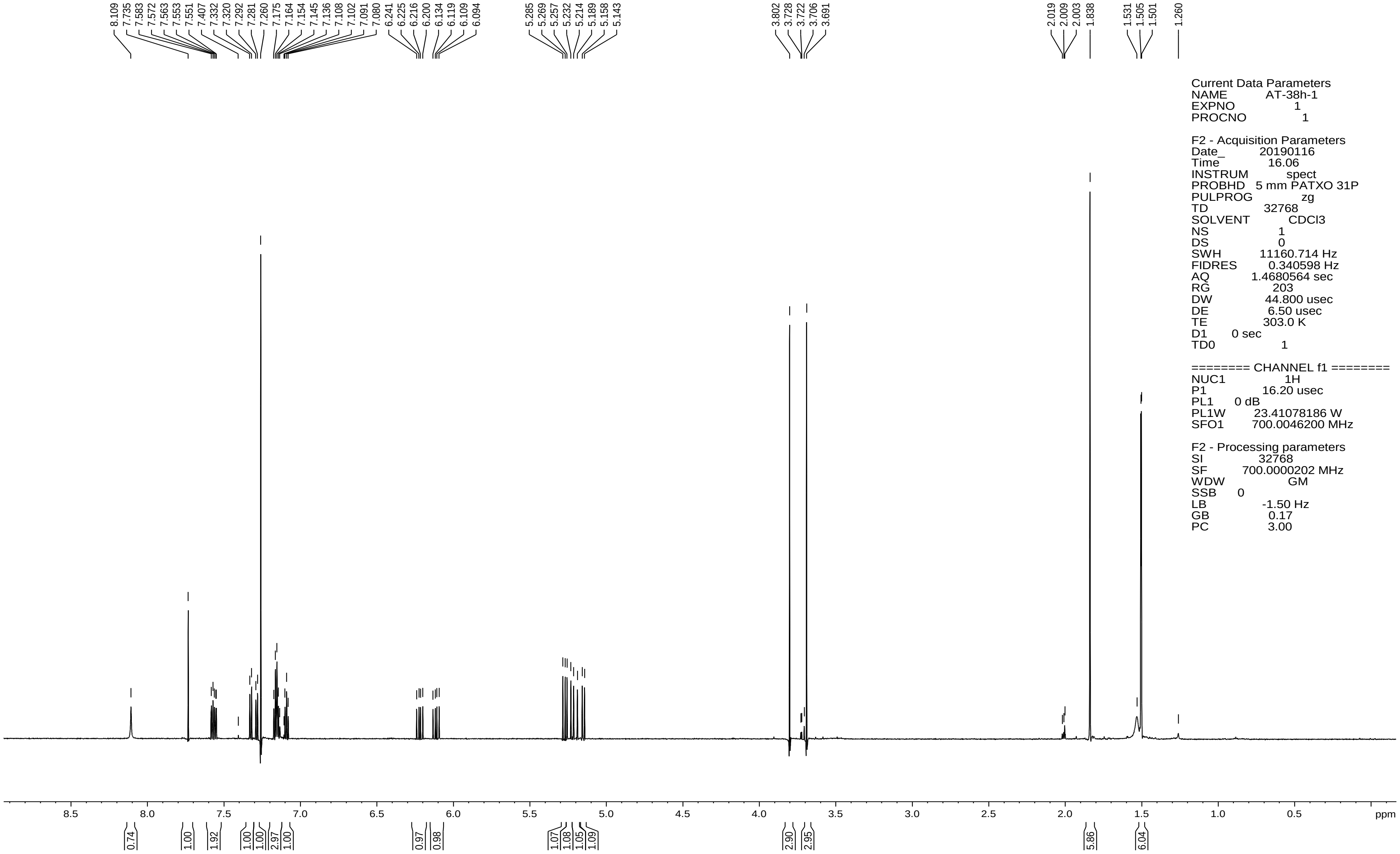

**Figure S21. $^{13}$C NMR (176 MHz, $CDCl_3$) spectrum of asterriquinone A3 (7)**

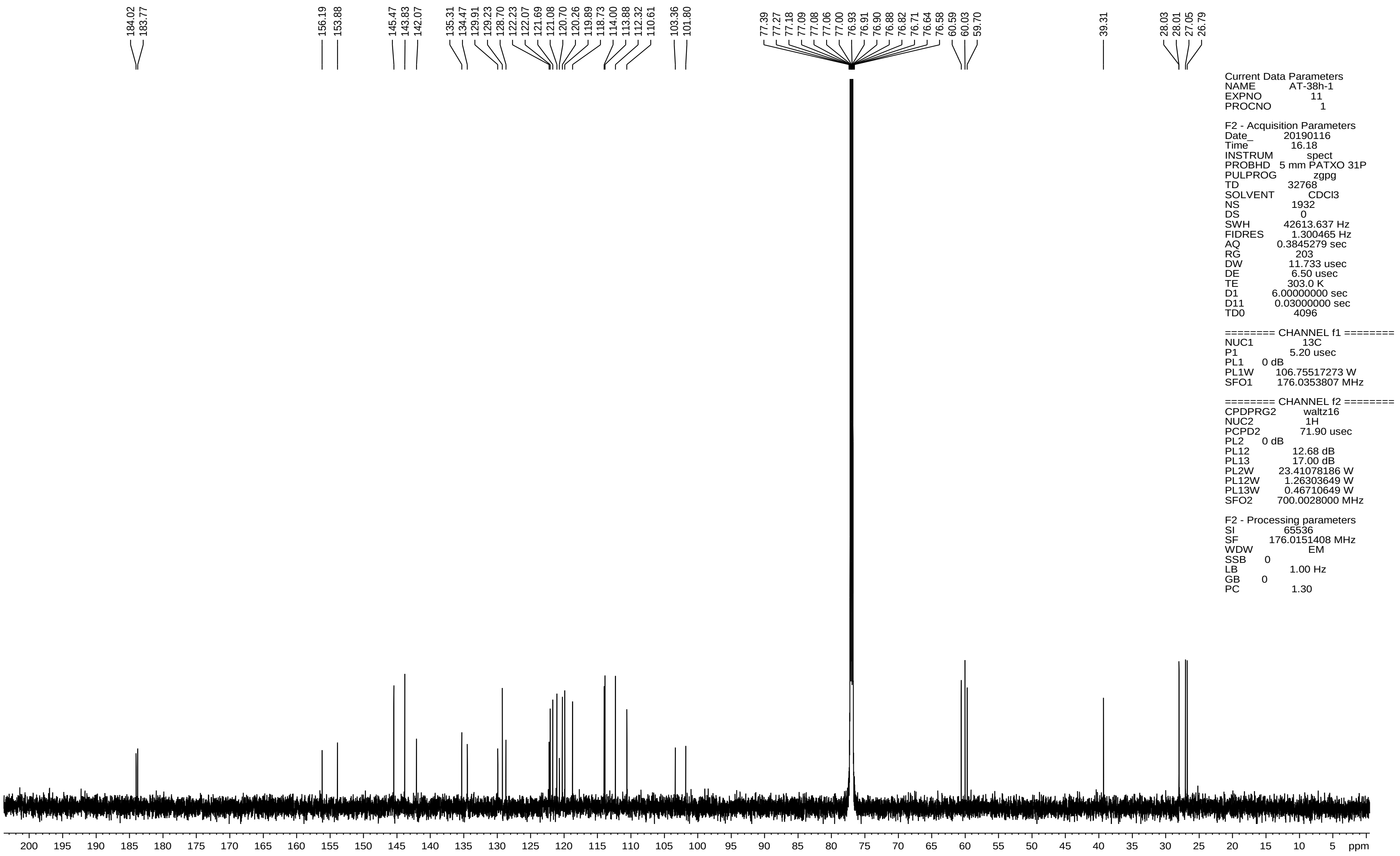

**Figure S22. $^{1}$H NMR (500 MHz, $CDCl_3$) spectrum of asterriquinone B4 (8)**

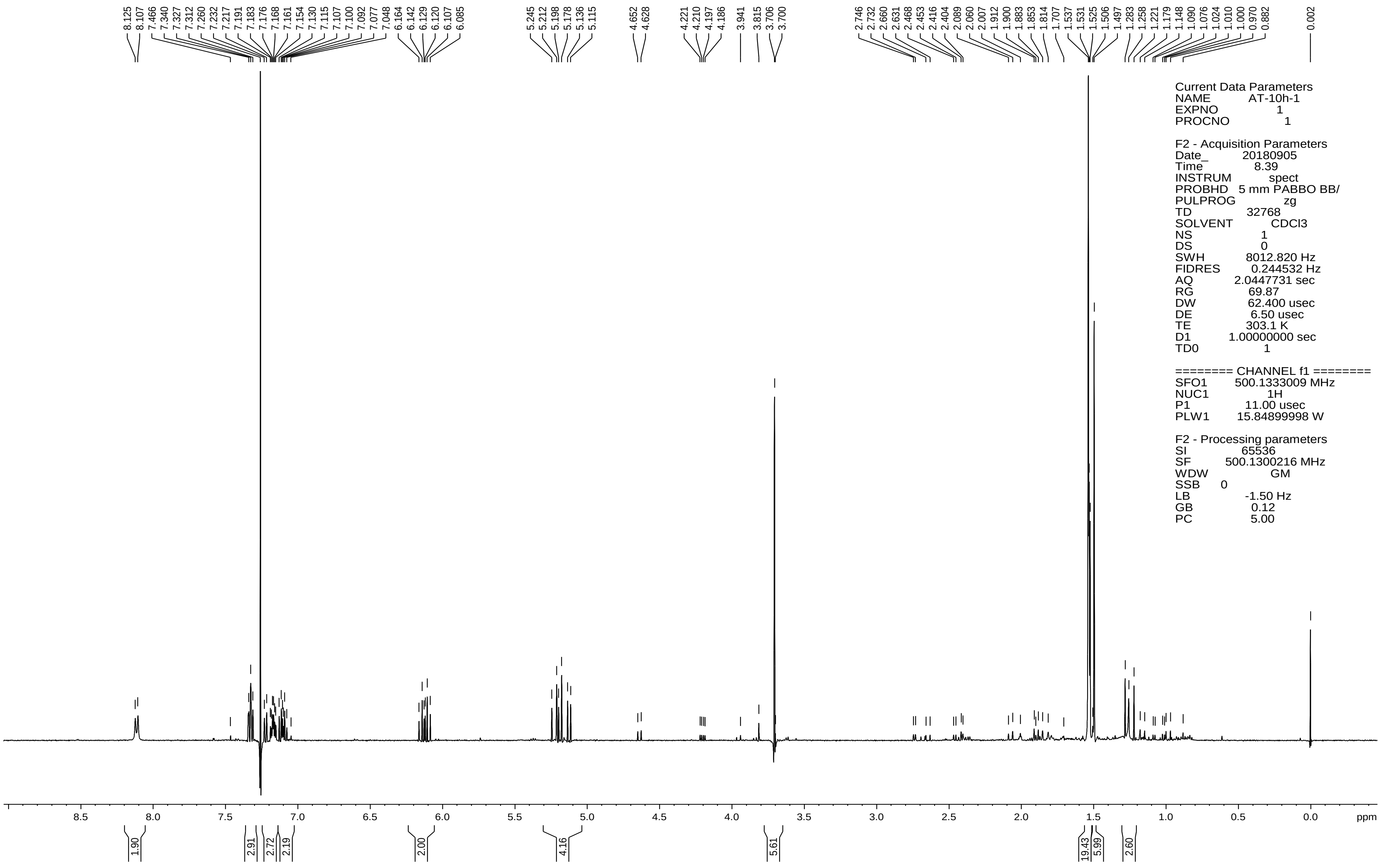

**Figure S23. $^{13}$C NMR (125 MHz, $CDCl_3$) spectrum of asterriquinone B4 (8)**

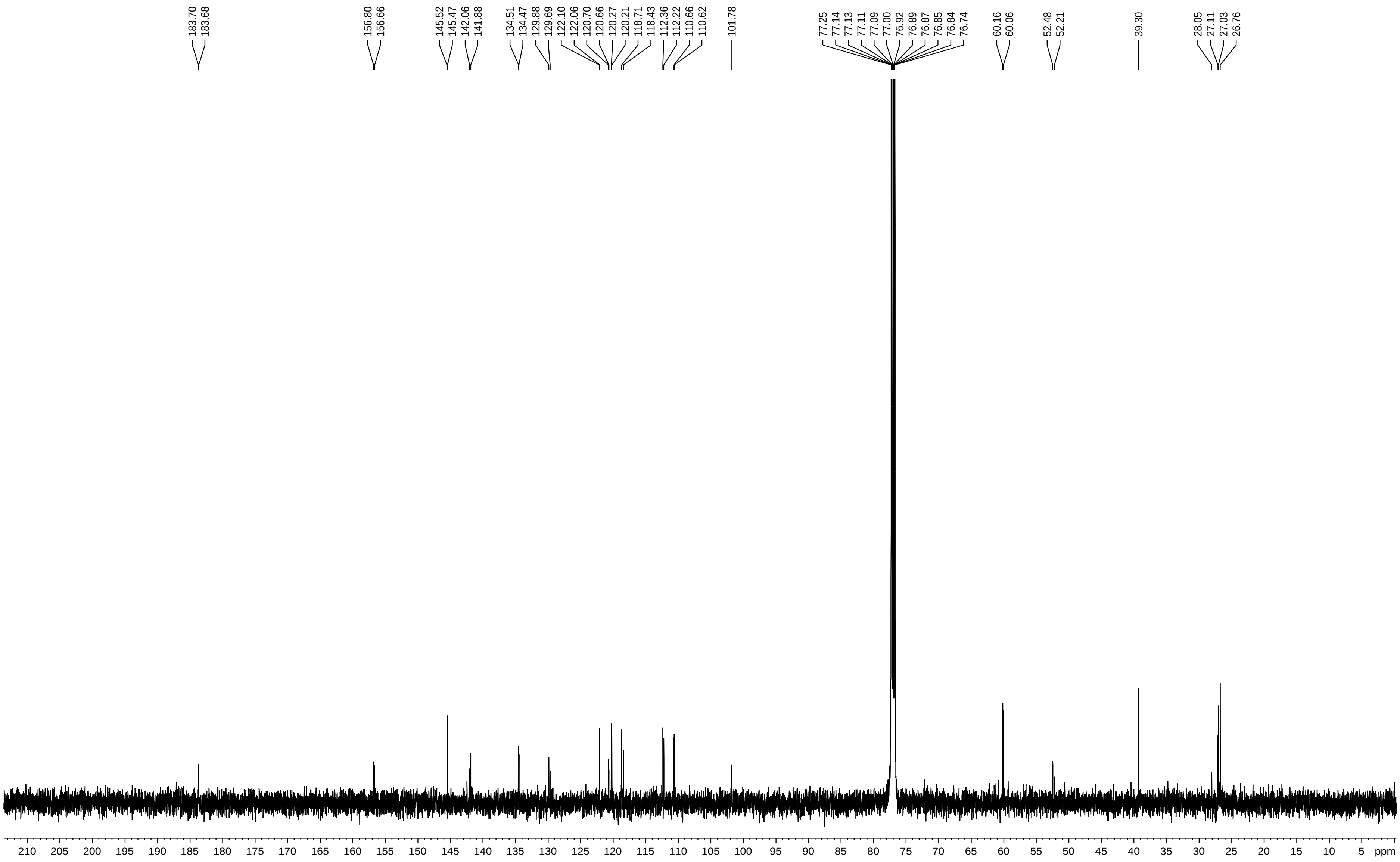

**Figure S24. $^1$H NMR (500 MHz, $CDCl_3$) spectrum of asterriquinone C1 (9)**

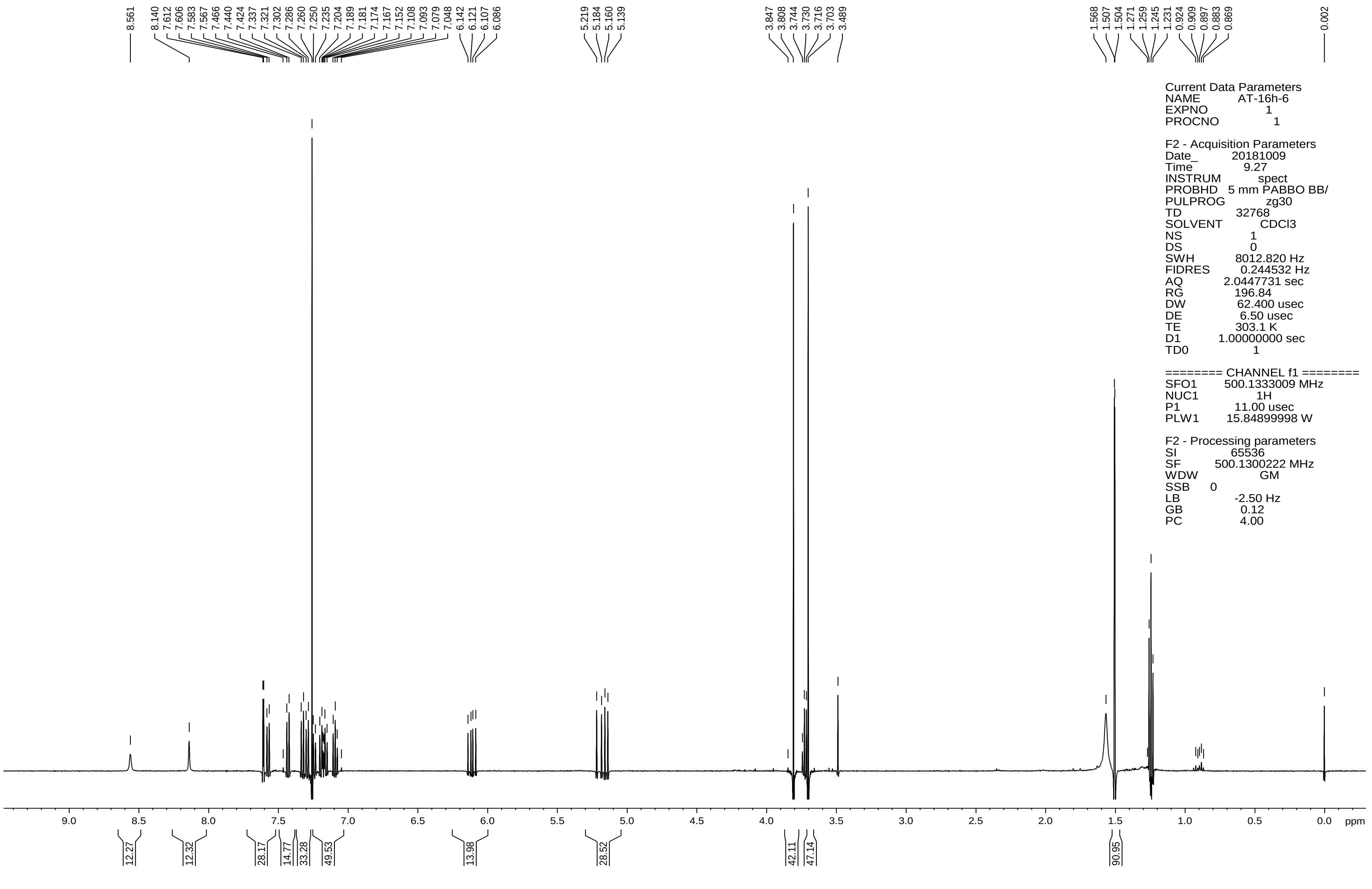

**Figure S25. $^{13}$C NMR (125 MHz, $CDCl_3$) spectrum of asterriquinone C1 (9)**

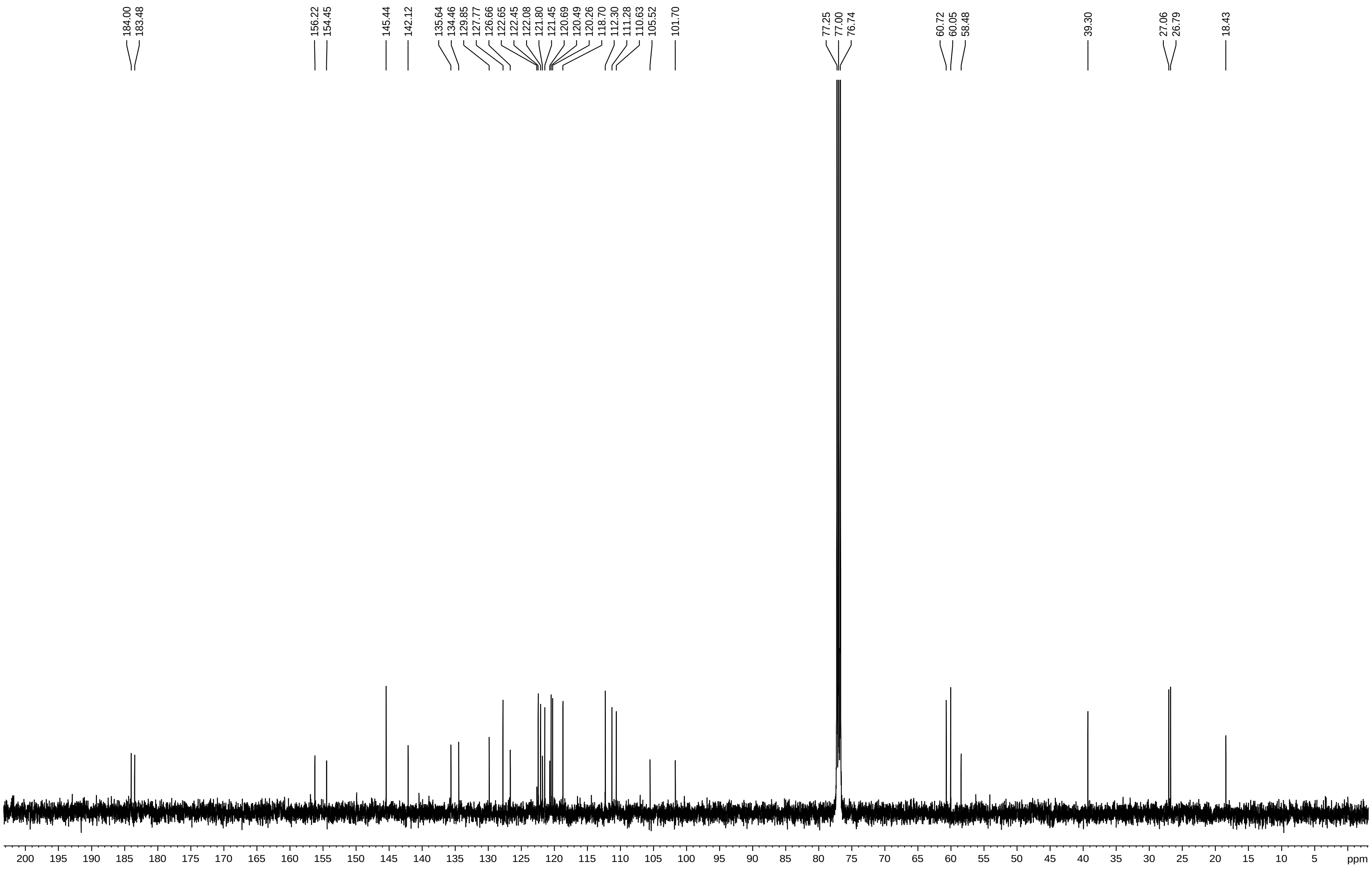

# Figure S26. $^1$H NMR (700 MHz, $CDCl_3$) spectrum of asterriquinone C2 (10)

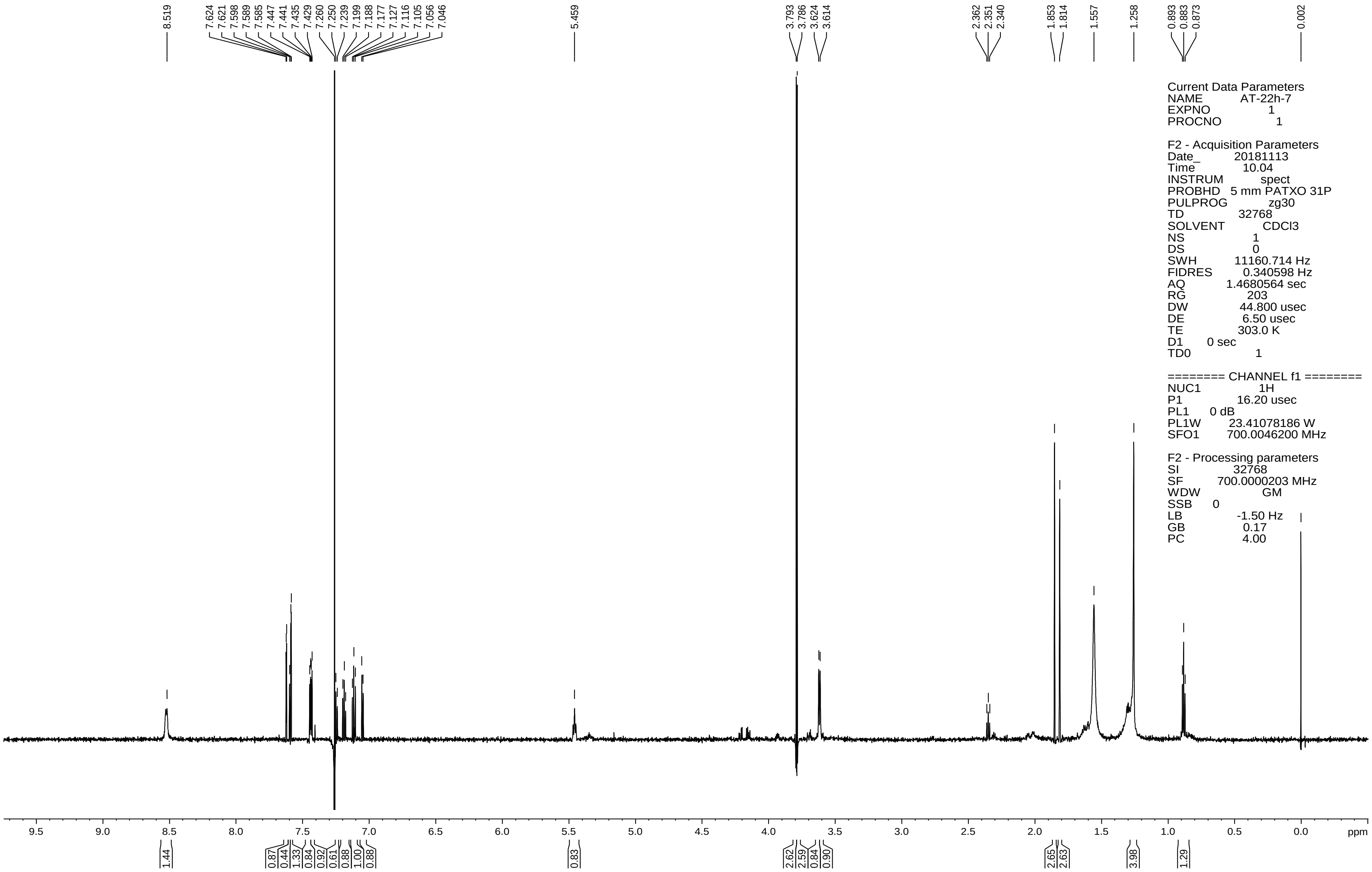

## Figure S27. $^{13}C$ NMR (176 MHz, $CDCl_3$) spectrum of asterriquinone C2 (10)

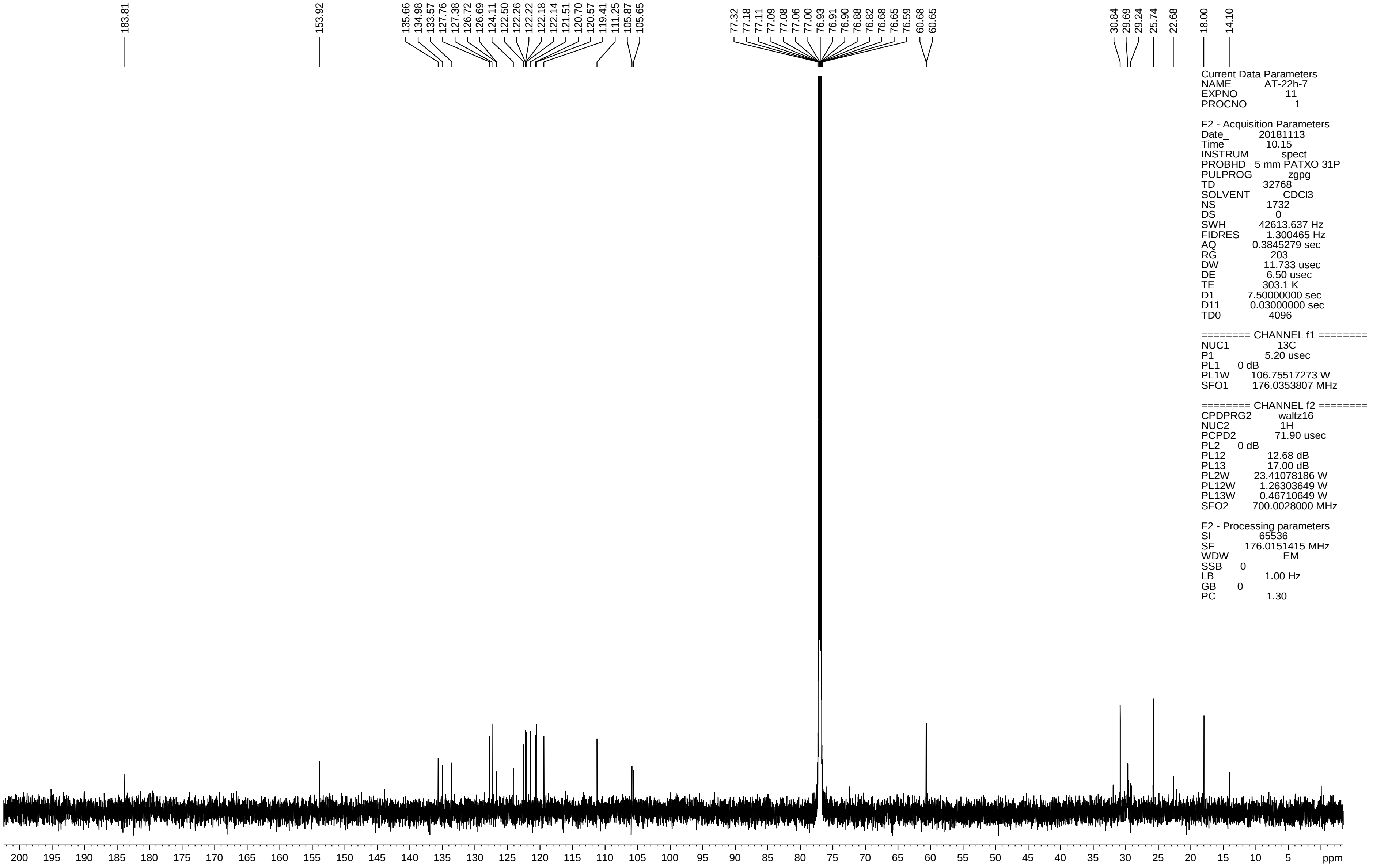

**Figure S28. $^{1}$H NMR (700 MHz, Acetone-$d_6$) spectrum of asterriquinone D (11)**

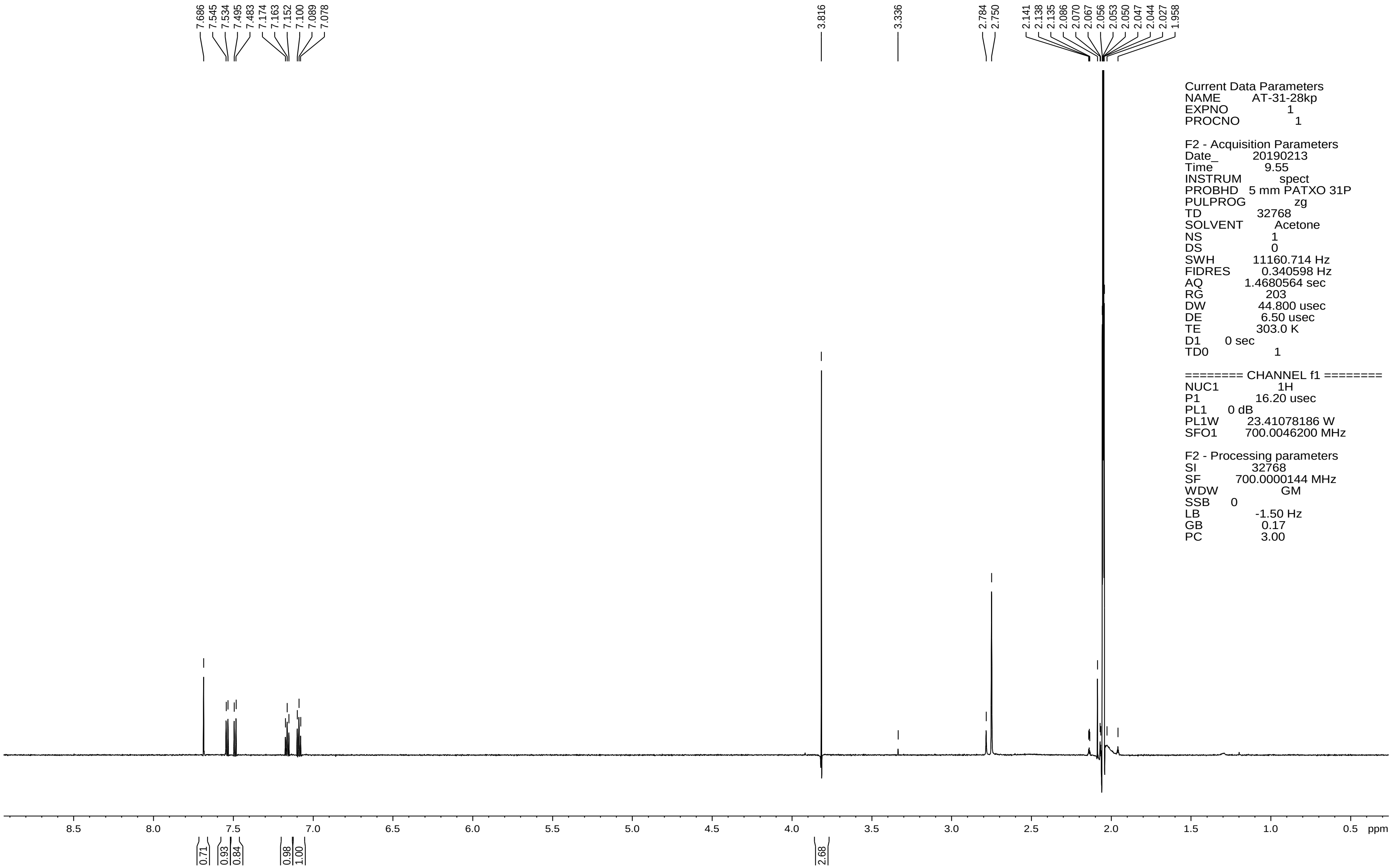

**Figure S29. $^{13}$C NMR (176 MHz, Acetone-$d_6$) spectrum of asterriquinone D (11)**

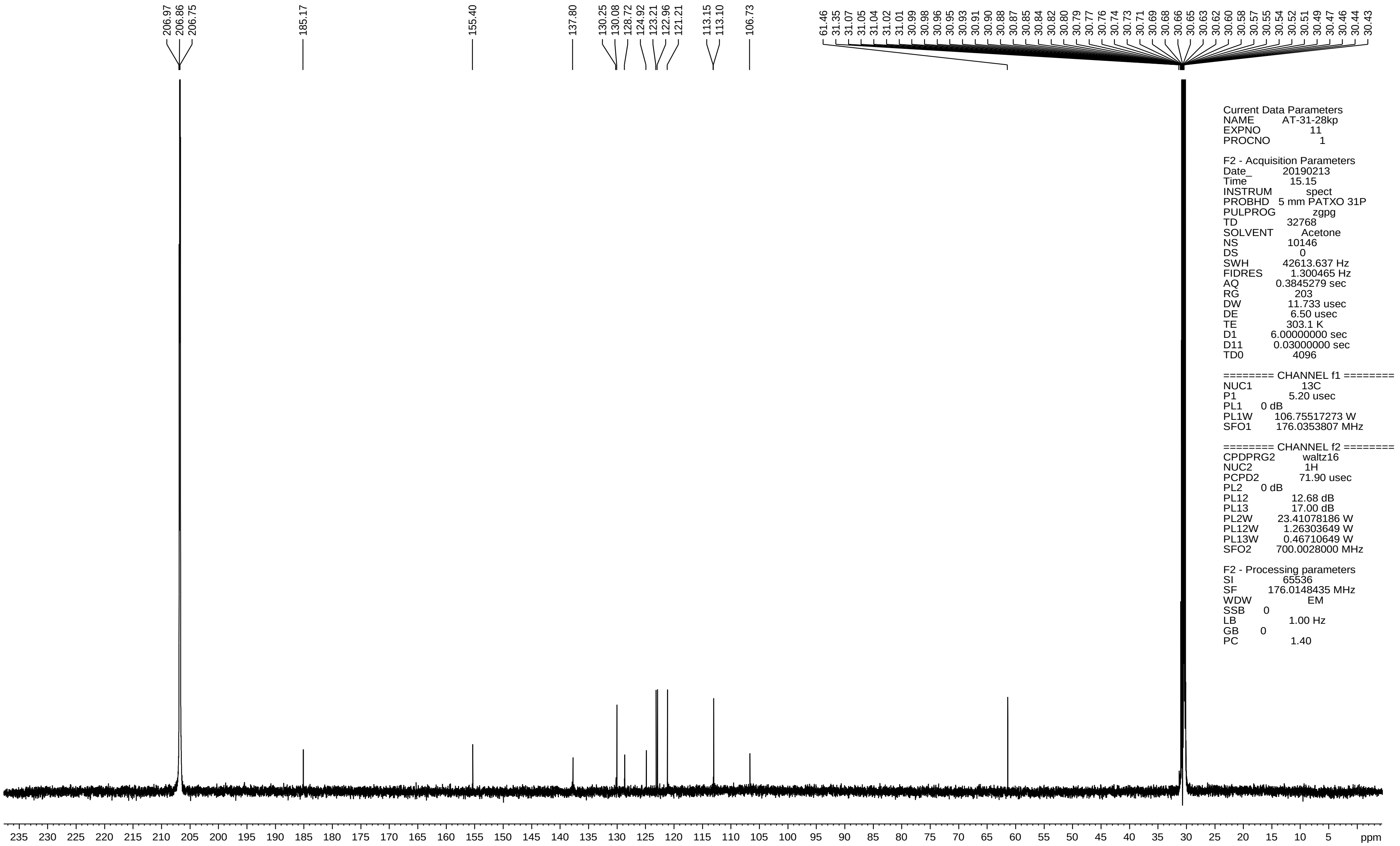

## Figure S30. $^{1}$H NMR (700 MHz, DMSO-$d_6$) spectrum of 1,2,5-trihydroxy-7-methyl-9,10-anthraquinone (13)

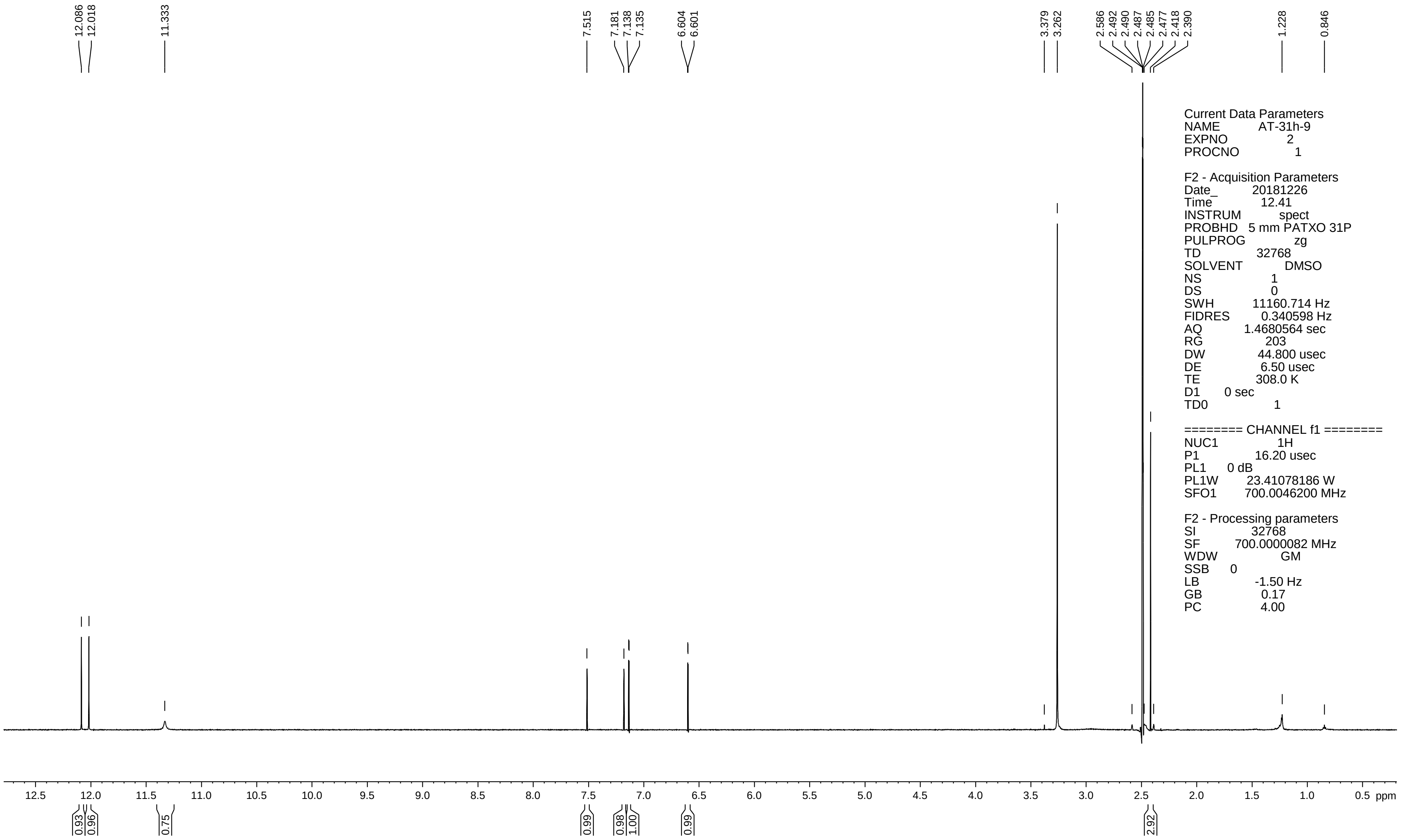

**Figure S31.** $^{13}$C NMR (176 MHz, DMSO-$d_6$) spectrum of 1,2,5-trihydroxy-7-methyl-9,10-anthraquinone (13)

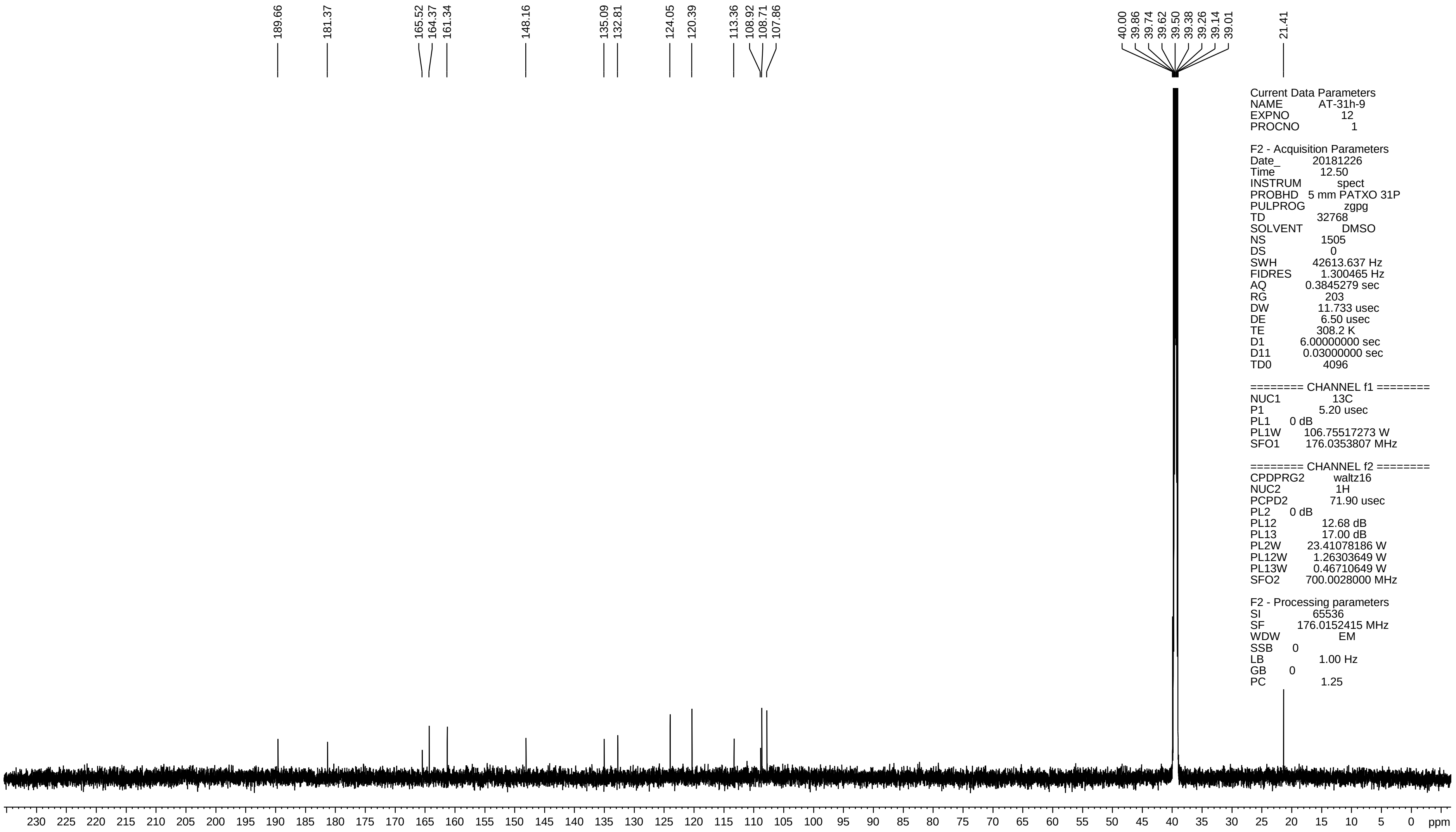

# Figure S32. $^{1}$H NMR (700 MHz, $CDCl_3$) spectrum of 4-hydroxy-3-(3-methylbut-2-enyl)benzaldehyde (14)

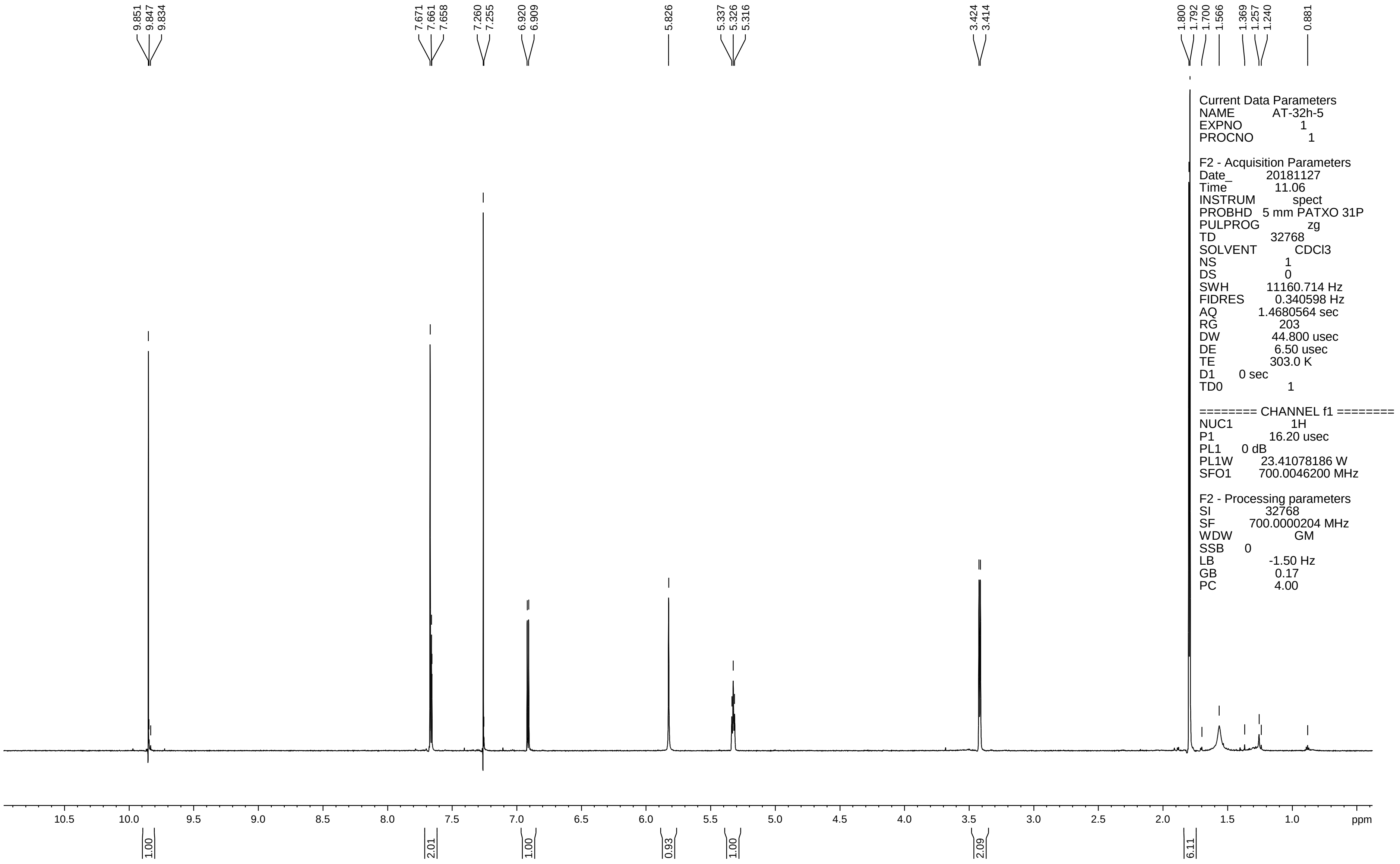

**Figure S33. $^{13}C$ NMR (176 MHz, $CDCl_3$) spectrum of 4-hydroxy-3-(3-methylbut-2-enyl)benzaldehyde (14)**

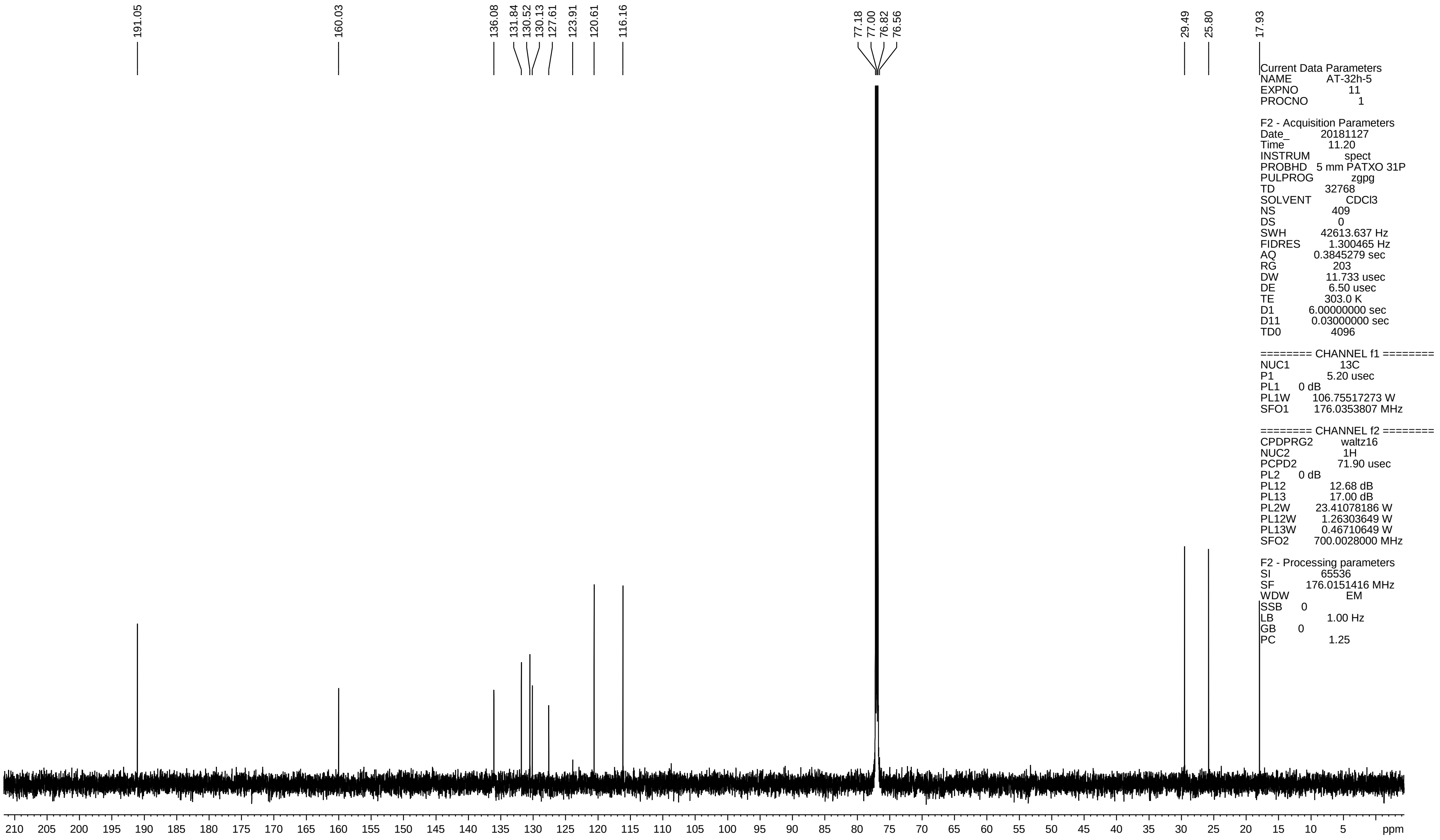

**Figure S34. $^{1}$H NMR (500 MHz, $CDCl_3$) spectrum of questin (12)**

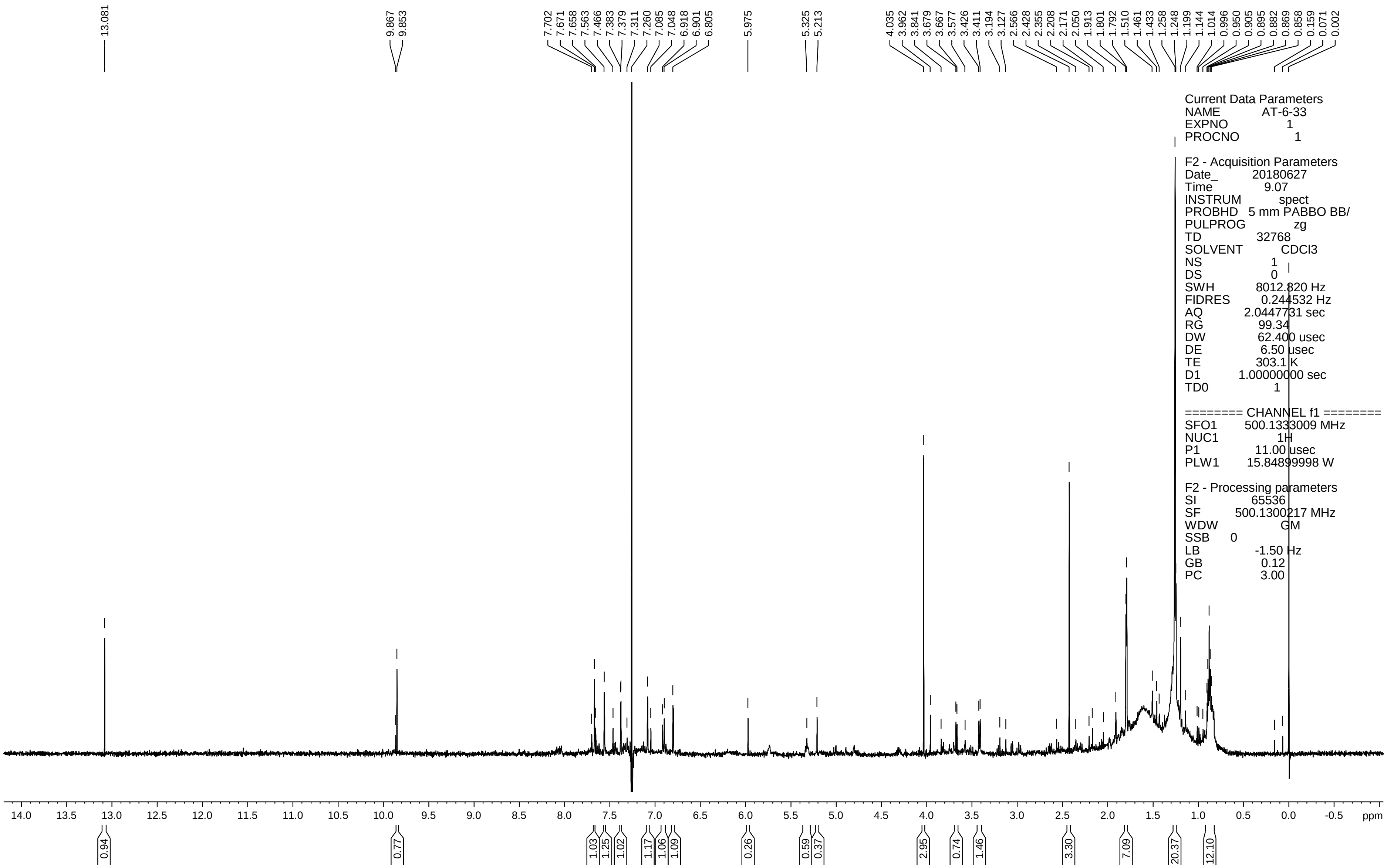

**Figure S35. $^{13}$C NMR (125 MHz, CDCl$_3$) spectrum of questin (12)**

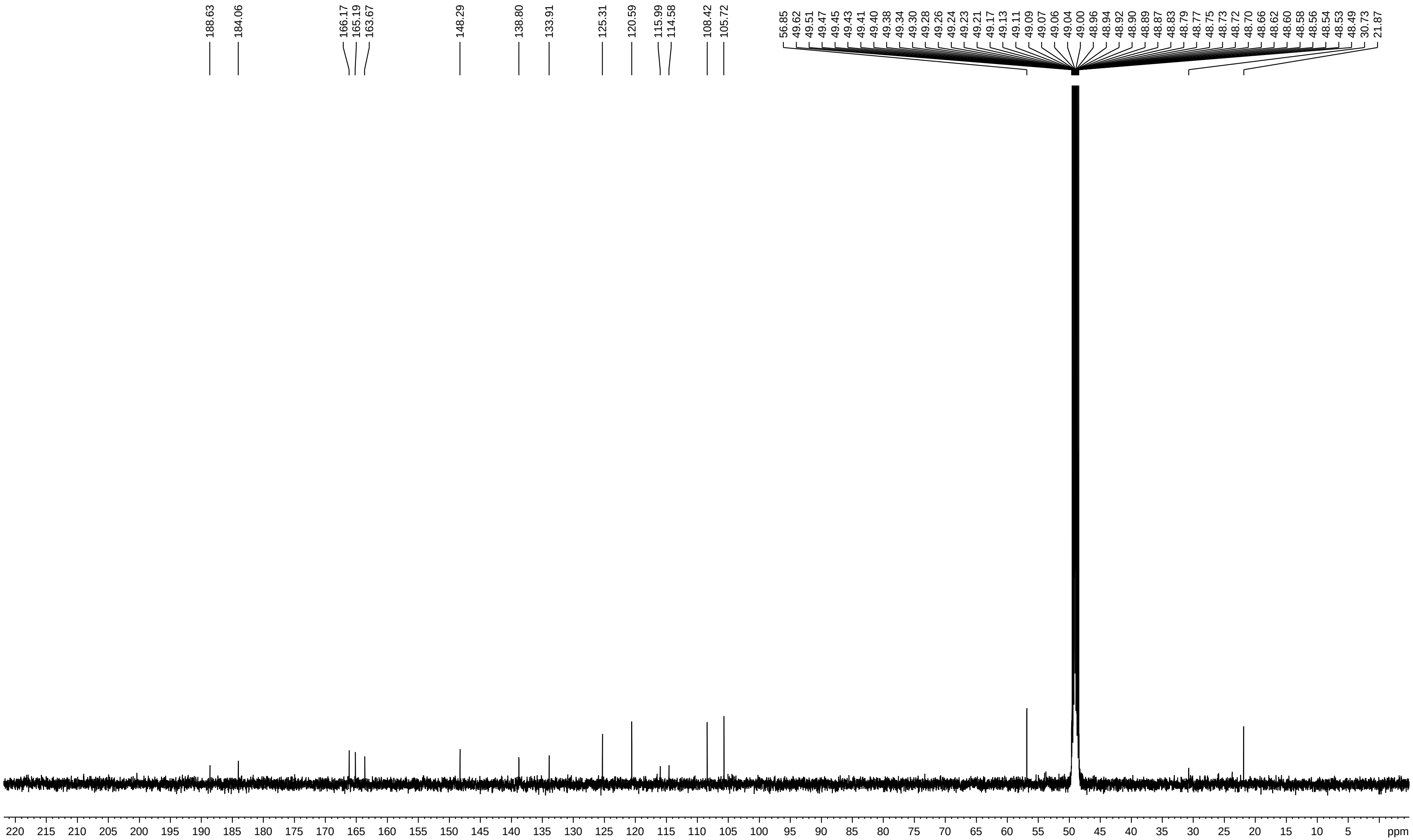

**Figure S36. $^{1}$H NMR (500 MHz, MeOD-$d_3$) spectrum of quadrone (15)**

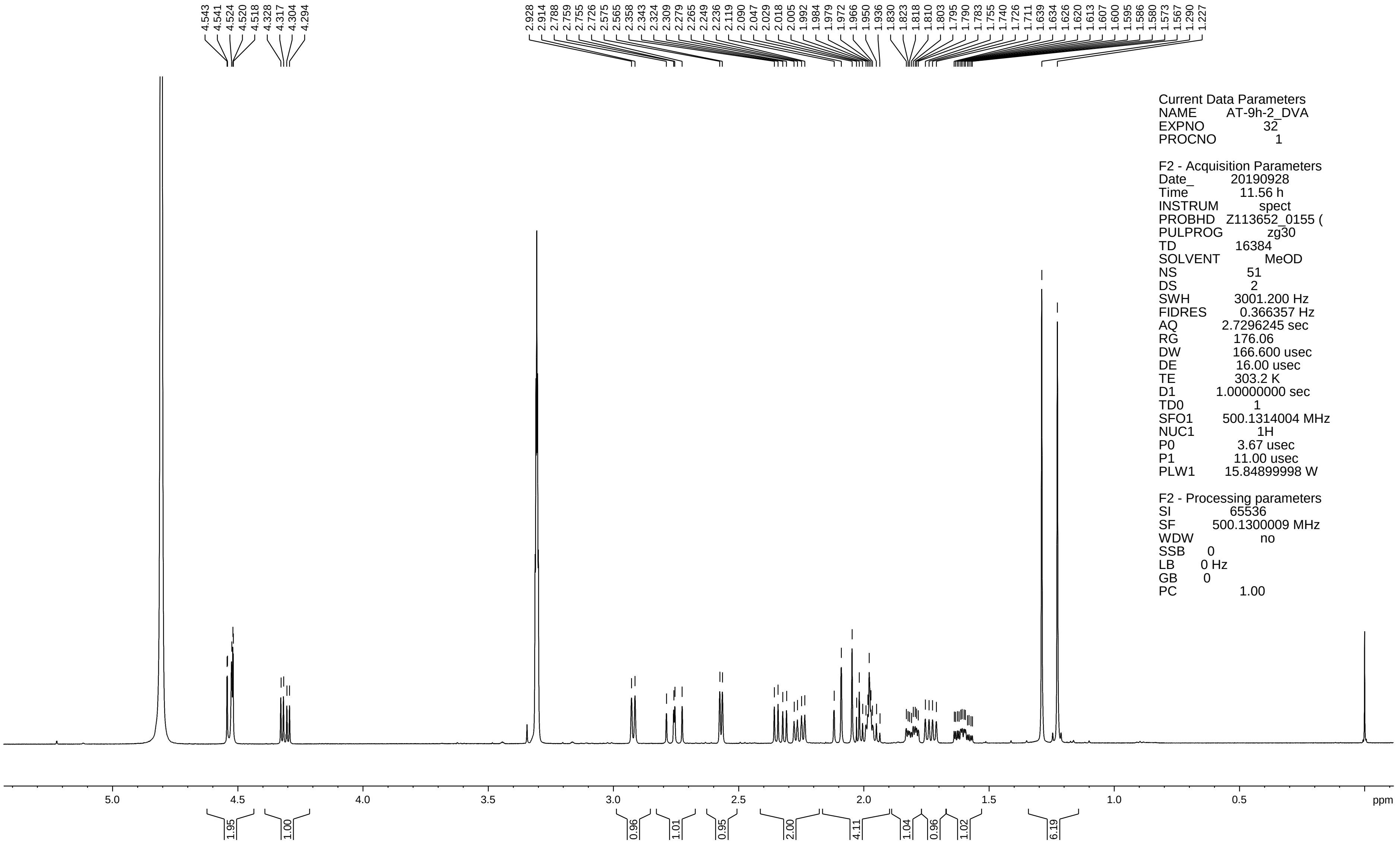

**Figure S37. $^{13}$C NMR (125 MHz, MeOD-$d_3$) spectrum of quadrone (15)**

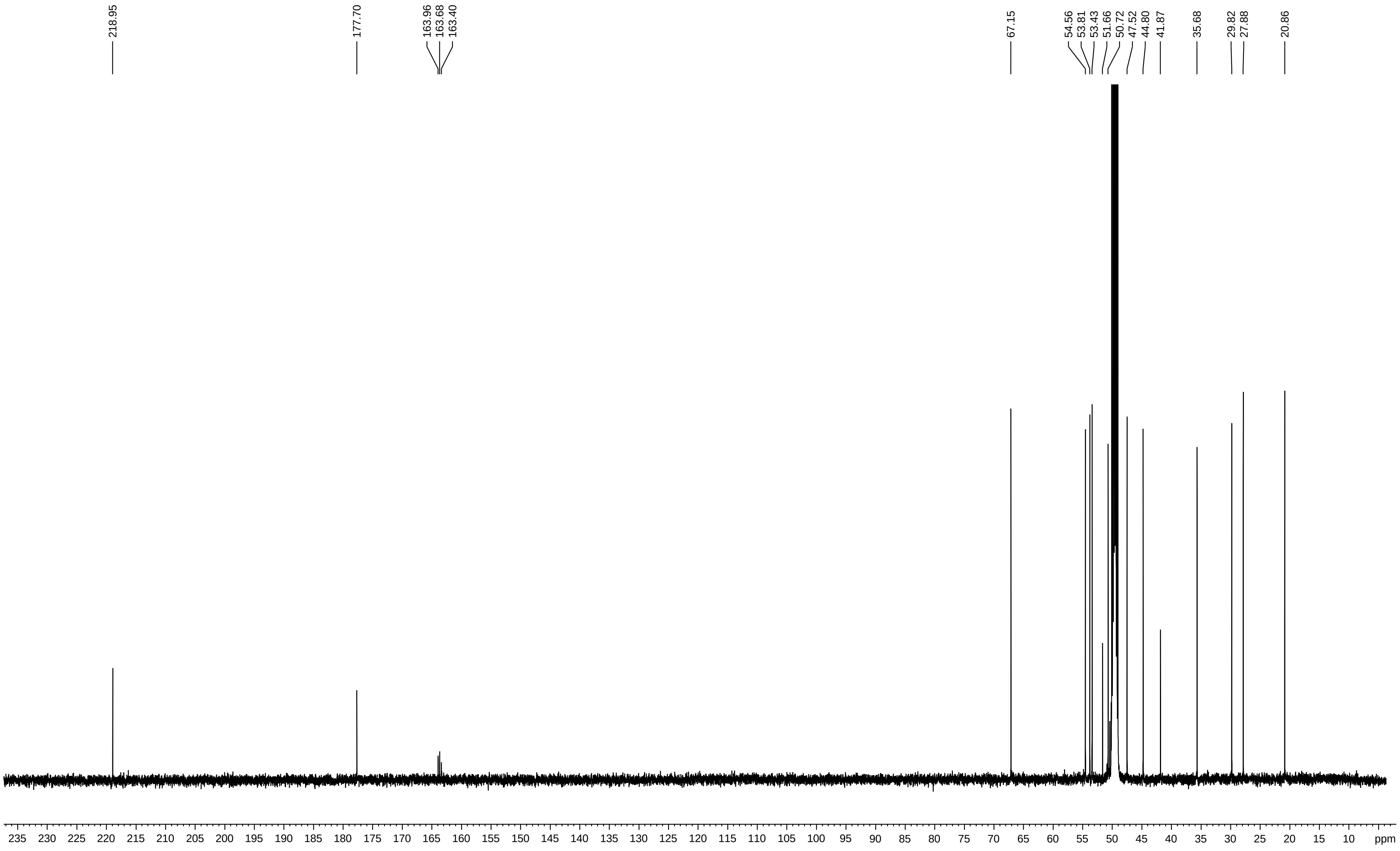

**Figure S38. $^{1}$H NMR (500 MHz, $CDCl_3$) spectrum of 6β-hydroxyergosta-4,7,22-trien-3-on (16)**

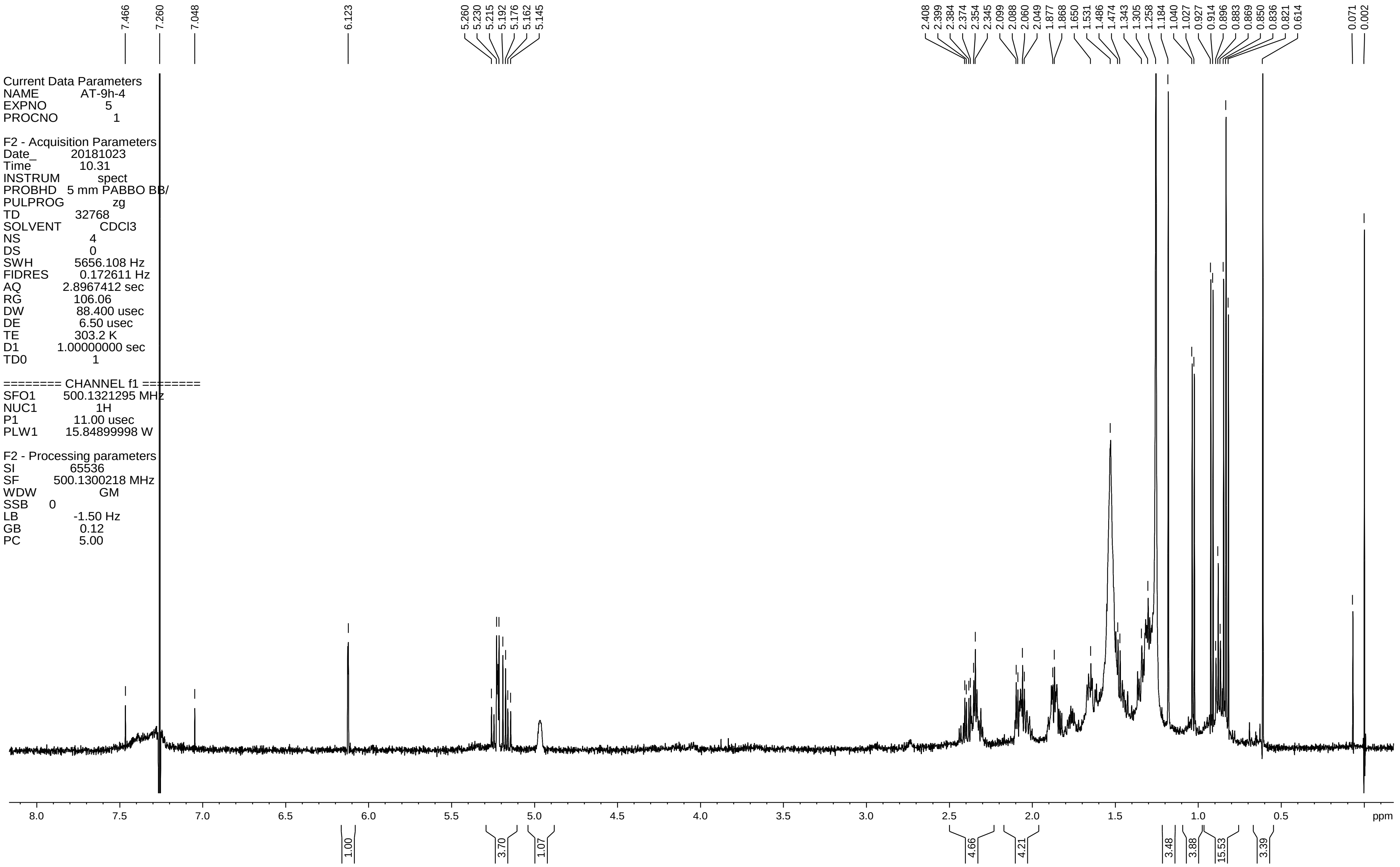

**Figure S39. $^{13}$C NMR (125 MHz, CDCl$_3$) spectrum of 6β-hydroxyergosta-4,7,22-trien-3-on (16)**

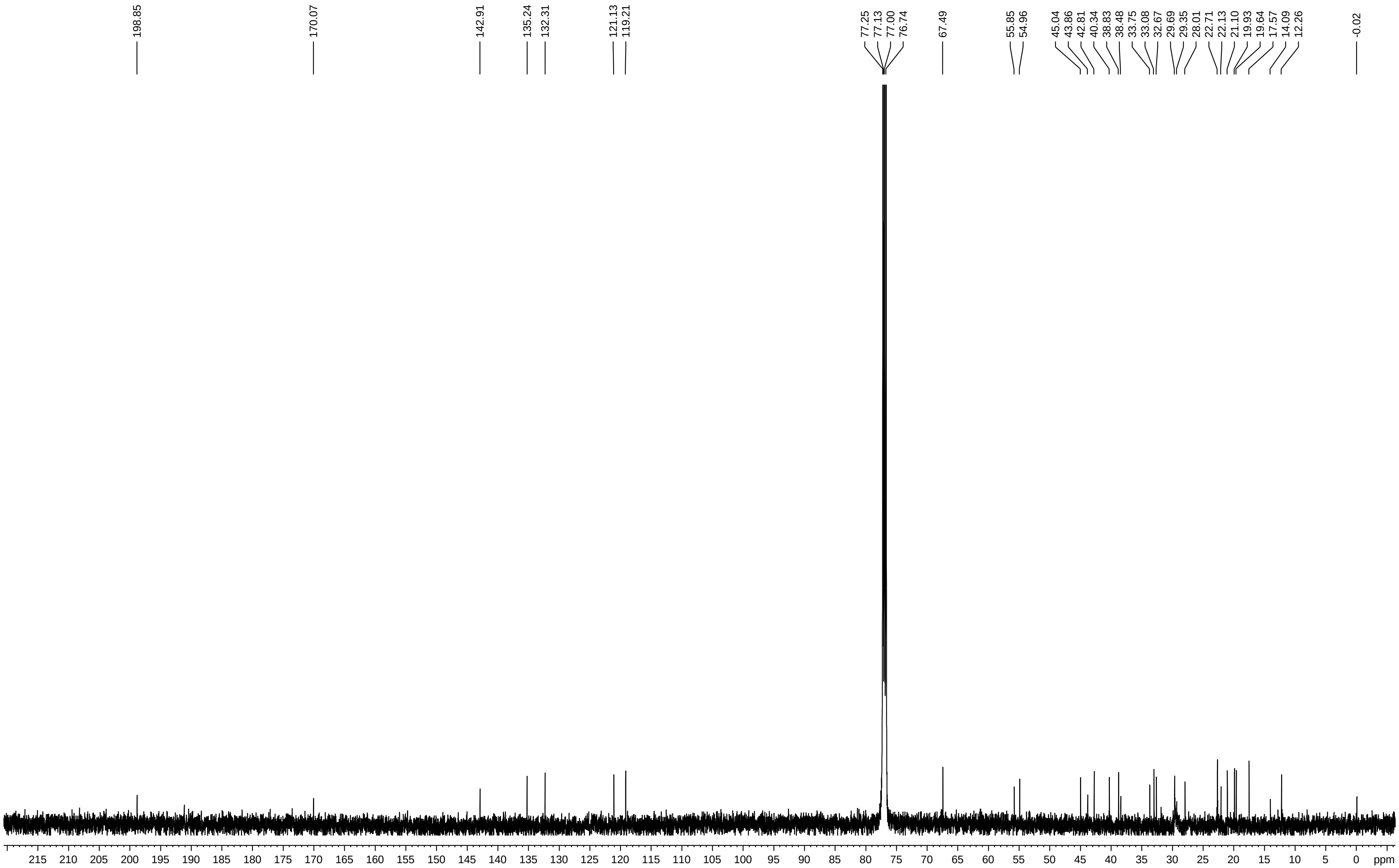

Supplement: Supplementary file 1 [file marinedrugs-18-00608-s001.pdf]
